# Mutation Status and Glucose Availability Affect the Response to Mitochondria-Targeted Quercetin Derivative in Breast Cancer Cells

**DOI:** 10.3390/cancers15235614

**Published:** 2023-11-28

**Authors:** Paweł Przybylski, Anna Lewińska, Iwona Rzeszutek, Dominika Błoniarz, Aleksandra Moskal, Gabriela Betlej, Anna Deręgowska, Martyna Cybularczyk-Cecotka, Tomasz Szmatoła, Grzegorz Litwinienko, Maciej Wnuk

**Affiliations:** 1 Faculty of Chemistry, University of Warsaw, Pasteura 1, 02-093 Warsaw, Poland; p.przybylski5@uw.edu.pl (P.P.); m.cybularczyk-cecotka@chem.uw.edu.pl (M.C.-C.); 2 Institute of Biotechnology, College of Natural Sciences, University of Rzeszow, Pigonia 1, 35-310 Rzeszow, Poland; alewinska@ur.edu.pl (A.L.); irzeszutek@ur.edu.pl (I.R.); dbloniarz@ur.edu.pl (D.B.); aleksandramoskal4@gmail.com (A.M.); gbetlej@ur.edu.pl (G.B.); aderegowska@ur.edu.pl (A.D.); 3 Center of Experimental and Innovative Medicine, University of Agriculture in Krakow, al. Mickiewicza 24/28, 30-059 Cracow, Poland; tomasz.szmatola@urk.edu.pl

**Keywords:** mito-quercetin, breast cancer, oxidative stress, AMPK, doxorubicin-induced senescence, senolysis

## Abstract

**Simple Summary:**

In the present study, we synthesized and characterized the physicochemical properties and anticancer activity of three non-polar, mitochondria-targeted derivatives of quercetin. Since all hydroxy groups are blocked, the compounds are not able to break the peroxidation of lipids; thus, high lipophilicity and strong interactions with lipid bilayers are principal factors affecting the bioactivity of the three derivatives. We focused on novel aspects of the bio-applications of mito-quercetin, which were never studied before. The novelty is based on the following: (a) cellular model—six different breast cancer cell lines (different mutation and receptor status); (b) different mito-quercetin derivatives with blocked “redox-active” groups that allow for a comparative analysis of previously published data on quercetin derivatives with free catechol moiety; (c) different experimental settings with high and low glucose concentrations to measure glucose availability and energetic stress; (d) the analysis of prosenescent and senolytic activity of mito-quercetin. For the first time, we show the importance of the genetic background, which, in this case, is the mutation status of breast cancer cells for the activity of quercetin derivatives, and we show that mito-quercetin is more effective than quercetin in the elimination of breast cancer cells with different mutation status.

**Abstract:**

Mitochondria, the main cellular power stations, are important modulators of redox-sensitive signaling pathways that may determine cell survival and cell death decisions. As mitochondrial function is essential for tumorigenesis and cancer progression, mitochondrial targeting has been proposed as an attractive anticancer strategy. In the present study, three mitochondria-targeted quercetin derivatives (mitQ3, 5, and 7) were synthesized and tested against six breast cancer cell lines with different mutation and receptor status, namely ER-positive MCF-7, HER2-positive SK-BR-3, and four triple-negative (TNBC) cells, i.e., MDA-MB-231, MDA-MB-468, BT-20, and Hs 578T cells. In general, the mito-quercetin response was modulated by the mutation status. In contrast to unmodified quercetin, 1 µM mitQ7 induced apoptosis in breast cancer cells. In MCF-7 cells, mitQ7-mediated apoptosis was potentiated under glucose-depleted conditions and was accompanied by elevated mitochondrial superoxide production, while AMPK activation-based energetic stress was associated with the alkalization of intracellular milieu and increased levels of NSUN4. Mito-quercetin also eliminated doxorubicin-induced senescent breast cancer cells, which was accompanied by the depolarization of mitochondrial transmembrane potential. Limited glucose availability also sensitized doxorubicin-induced senescent breast cancer cells to apoptosis. In conclusion, we show an increased cytotoxicity of mitochondria-targeted quercetin derivatives compared to unmodified quercetin against breast cancer cells with different mutation status that can be potentiated by modulating glucose availability.

## 1. Introduction

Functional mitochondria, hubs of energetics, metabolite production, redox homeostasis, and cellular signaling are essential for the maintenance of the tumorigenic potential of cancer cells [1,2,3,4]. For example, it has been shown that cancer cells in which the respiratory function was compromised due to mitochondrial DNA (mtDNA) deletion are capable of in vivo mitochondrial genome acquisition to re-establish respiration and tumor-initiating efficacy [4]. This may indicate that oxidative phosphorylation is essential for cancer progression; for this reason, oxidative phosphorylation was suggested as a potential target for cancer therapy [3,4,5]. Indeed, the pharmacological inhibition of oxidative phosphorylation may have anticancer effects [3], and one of the possible strategies is to design mitochondria-targeted agents: for example, cationic lipophilic molecules interfering with mitochondrial function by means of conjugation with triphenyl phosphonium cationic (TPP^+^) groups [6,7]. Positively charged and lipophilic TPP^+^-conjugated compounds can then be specifically accumulated in the mitochondrial matrix [6]. For example, the antiproliferative effects of two mitochondria-targeted drugs, namely 3-carboxyl proxyl nitroxide (mito-CP) and mito-metformin, were mediated by a decrease in intracellular ATP levels, inhibition of ATP-linked oxygen consumption, activation of AMP-activated protein kinase (AMPK), and phosphorylation-associated suppression of the mTOR target ribosomal protein S6 kinase B1 (RPS6KB1 or p70S6K) in colon cancer cells [8]. These mechanisms were accompanied by the induction of mitophagy, as judged by the activation of Unc-51-like autophagy-activating kinase 1 (ULK1), which affected the morphology of mitochondria and decreased the mitochondrial transmembrane potential (MMP) [8]. MitoTam, a mitochondria-targeted derivative of tamoxifen, inhibited complex I-driven respiration, disrupted respiratory supercomplexes, depolarized MMP, promoted reactive oxygen species (ROS) production, and in turn stimulated cell death in HER2^high^ breast cancer cells both in vitro and in vivo [9].

Quercetin, a natural redox-active polyphenol, may behave as a pro- or antioxidant depending on the cellular context [10,11] and interfere with mitochondrial function, as judged by the quercetin-mediated inhibition of the activity of ATP synthase [12], depolarization of MMP-driven apoptosis [13,14], and AMPK activation, leading to the inhibition of cell proliferation and cytotoxicity against different breast cancer cell lines [15]. In order to increase the mitochondrial accumulation of quercetin, its TPP^+^-conjugated derivatives were obtained [16], and such mitochondriotropic species inhibited the activity of ATPase and affected respiration in permeabilized rat liver mitochondria [16,17]. The same research group documented a cytotoxic potential of mito-quercetin derivatives against C-26 mouse colon cancer cells and human Jurkat T cell leukemia cells, as judged by MTT assay or Annexin V staining [16,18,19]. These pioneering studies were focused on mito-quercetins with TPP^+^ conjugated via n-butyl linked to an oxygen atom at position 3 or 7, whereas other hydroxy groups remained free or were completely acetylated to hinder metabolism and improve solubility (a slow enzymatic deacetylation in the rat liver mitochondria recovers them into redox-active groups) [16,17]. The presence of a free catechol moiety (3′,4′-dihydroxy group) is necessary for the antioxidant and pro-oxidant function of quercetin (and catechols in general) [20], depending on the concentration and the presence of metal cations and protonation/deprotonation. For example, 7*O*-mito-quercetin applied to a micromolar concentration produced hydrogen peroxide (H_2_O_2_) as an oxidant and selectively induced the necrosis of fast-growing cells (C-26 mouse colon cancer cells and mouse embryonic fibroblasts, MEFs) while slow-growing MEFs survived [19]. Nonetheless, an intriguing question arises: does the free catechol moiety (necessary for antioxidant/pro-oxidant activity) account for the chemotherapeutic effect of the mitochondriotropic quercetin? In order to obtain insights into this problem, we designed and prepared three mitochondria-targeted quercetin derivatives with a catechol moiety permanently blocked and resistant to enzymatic hydrolysis and with one TPP^+^ attached at position 3 (mitQ3), 5 (mitQ5), or 7 (mitQ7), while the two remaining hydroxy groups were acetylated.

Taking into account that quercetin is active against breast cancer cells [13,14,15] and that glucose deprivation potentiates the antiproliferative action of some anticancer drugs (such as paclitaxel and doxorubicin) in breast cancer cells [21], in the present study, we tested a combination of these two effects on six phenotypically different breast cancer cell lines, namely ER-positive MCF-7, HER2-positive SK-BR-3, and four triple-negative (TNBC) MDA-MB-231, MDA-MB-468, BT-20, and Hs 578T cells. Initially, mitQ3, mitQ5, and mitQ7 were used to analyze their effects on various glucose availability. With the knowledge of the activity of all three derivatives, in subsequent steps, the most active mitQ7 was selected to reveal the mechanisms of its anticancer action, namely redox imbalance and energetic stress. Furthermore, novel results on the pro-senescent (senescence-inducing potential) and senolytic (selective killing of senescent cells) activity of mitQ7 are presented.

## 2. Materials and Methods

### 2.1. Synthesis of Quercetin Derivatives

**Scheme 1:**
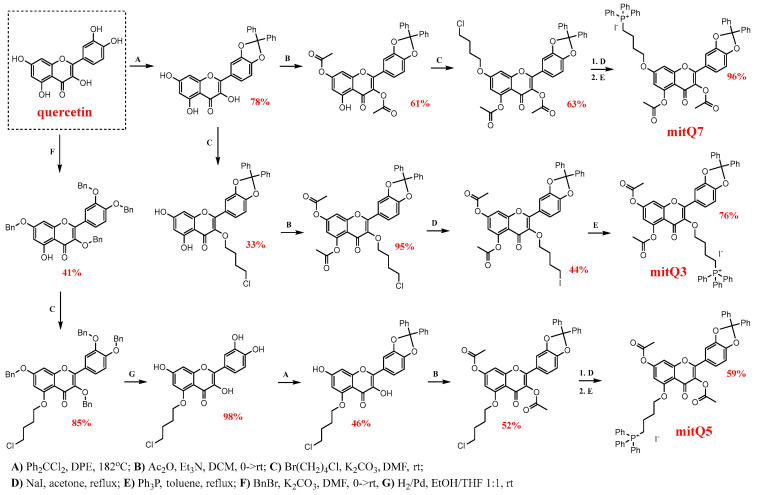
Multi-step pathways for synthesis of three derivatives of quercetin (mitQ3, mitQ5, and mitQ7) with butyl triphenylphosphonium cation attached at the positions O-3, O-5 and O-7. For brevity, for mitQ5 and mitQ7, the two last steps were marked with one arrow, but steps D and E were subsequently performed, as represented here for mitQ3. All abbreviations and reaction conditions, isolation and identification procedures are described in detail in the Supplementary Material S1.

The synthesis of three derivatives, namely mitQ3, mitQ5, and mitQ7, from quercetin as the starting material was designed based on the method described by Biasutto et al. [22]. A general scheme of synthesis is presented in Scheme 1. Detailed procedures and analytical techniques used for the identification of the products at each step of the synthesis are provided in Supplementary Material S1. All reagents and solvents were purchased from commercial suppliers and used without further purification. NMR spectra were recorded at room temperature using a Bruker 300 MHz spectrometer. High-resolution mass spectra were measured using an Agilent 6540 UHD Q-TOF, and the spectrum was recorded at the highest resolution of 4 GHz.

### 2.2. Preparation of Liposomes

Large unilamellar vesicles (LUVs) were obtained from multilamellar vesicles (MLVs) by a previously described extrusion procedure. A 65.3 mg portion of 1,2-dimyristoyl-*sn*-glycero-3-phosphocholine (DMPC) was dissolved in 1.5 mL of CHCl_3_ in a round-bottom flask. Then, 4 μL of methyl linoleate (MeLin) was added, the solvent was removed using a rotary evaporator, and the lipids were vacuum-dried overnight. The obtained lipid film was then suspended in buffers of pH 4.0, 6.0, 7.0, 8.0, and 10.0 (all components were inorganic: acetic, phosphate, and borate buffers were used); the final concentration of lipids was 2.74 mM MeLin and 20.2 mM DMPC. LUVs were obtained by multiple extrusions (at least 21 times) through polycarbonate membranes with a pore diameter of 100 nm in an Avanti Mini-Extruder (Avanti Polar Lipids Inc., Alabaster, AL, USA). Based on the DLS method, the size distribution of the LUVs was 170 ± 45 nm (in agreement with previously prepared LUVs [23]).

### 2.3. Methodology of Autoxidation Measurements

The reactivity of the peroxyl radicals was investigated during the peroxidation of LUVs. The rate of peroxidation under controlled conditions (37 °C, pH: 4.0, 6.0, 7.0, 8.0, and 10.0) was monitored as the oxygen uptake using a Biological Oxygen Monitor (Yellow Springs Instruments equipped with a oxygen electrode). Two milliliters of a liposomal suspension were transferred to the glass vessel equipped with a magnetic stirring bar, placed in a thermostatic bath and aerated (with stirring 480 rpm) for 10 min to obtain 100% saturation. The vessel was covered with an oxygen electrode. A Clark-type polarographic oxygen probe immersed in a Teflon plunger was placed in the vessel, and the oxygen content was continuously recorded. The peroxidation of MeLin/DMPC was initiated with the water-soluble initiator azo-bis-2-amidinepropane (ABAP) added with a microliter syringe (0.5 M aqueous stock solution) to obtain a final concentration of 10 mM ABAP in the vessel. After 10% oxygen was consumed, 10 μL of methanolic solution of a reference antioxidant (quercetin) or mito-quercetin derivative (mitQ) was added. The final concentration of each compound tested was 1 μM. A detailed description of the evaluation of the kinetic parameters is provided elsewhere [24,25].

### 2.4. Microcalorimetric Measurements

For calorimetric experiments, the method for the preparation of DMPC LUVs was the same as described above with the difference that methyl linoleate was not added and phosphate buffer (pH 7.4) was used. The thermal behavior of pure DMPC LUVs was compared with DMPC LUVs containing 50, 100, and 150 μM mitQ. DSC measurements were performed with a MicroCal PEAQ-DSC. The volume of the sample and the reference cells was 0.250 mL, and the reference cell was filled with PBS buffer of pH 7.4. Measurements were performed in the temperature range 15 to 30 °C at 3.8 bar excess pressure with a scanning rate of 0.05 °C/min. After preincubation of the sample at 30 °C for 10 min, at least 3–4 downscans and upscans (alternate cooling and heating scans) were performed to check the stability of the system and the reproducibility of the DMPC phase behavior [26]. The downscans and upscans were reproducible, and thus, the samples reached equilibrium during each experiment. The analysis of the DSC data was performed in Origin 7.0 software. The enthalpies were calculated from the peak areas using the template written in Origin 7.0.

### 2.5. In Silico Analysis of Putative Cellular Targets of Quercetin Derivatives

SwissTargetPrediction (http://www.swisstargetprediction.ch/) (accessed on 11 July 2023) [27] was used to determine macromolecular targets of quercetin derivatives compared to unmodified quercetin.

### 2.6. Cell Lines and Culture Conditions

Six breast cancer cell lines characterized by different receptor status were analyzed, namely ER-positive MCF-7 (HTB-22™), HER2-positive SK-BR-3 (HTB-30™), as well as four triple-negative (TNBC) MDA-MB-231 (HTB-26™), MDA-MB-468 (HTB-132™), BT-20 (HTB-19™), and Hs 578T (HTB-126™) (ATCC, Manassas, VA, USA). For comparative studies of mitochondria-targeted quercetin derivatives (mitQ3, 5, and 7), human normal proliferatively active fibroblasts were used (BJ cells with population doubling levels between 30 and 40, CRL-2522™, ATCC, Manassas, VA, USA) [28]. Cells were grown in DMEM medium containing 10% (*v/v*) FBS and 100 U/mL penicillin, 0.1 mg/mL streptomycin, and 0.25 μg/mL amphotericin B (Corning, Tewksbury, MA, USA) in a 5% CO_2_ atmosphere at 37 °C. To study the effect of glucose availability, two glucose concentrations in DMEM medium were used, namely 4.5 g/L (high-glucose DMEM) and 1 g/L (low-glucose DMEM) (Corning, Tewksbury, MA, USA). All cell lines were cultured in DMEM medium using the same concentrations of supplements as stated above to study the effect of a single variable, namely glucose concentration. A metabolic activity MTT test was applied to select the most effective mitochondria-targeted quercetin derivative. The quercetin derivatives obtained from two independent syntheses were used. Briefly, 5 × 10^3^ and 1 × 10^4^ of cancer and normal cells per well in 96-well plates were treated for 24 h with unmodified quercetin (Q, Merck KGaA, Darmstadt, Germany) and mitQ3, 5 and 7 (at concentrations of 0.1, 1, 5 and 10 µM). The MTT test was performed using a dedicated absorbance microplate reader. The effect of solvent (DMSO) was also analyzed. Under control conditions (CTR), metabolic activity is considered as 100%. Based on the MTT results, 1 µM mitQ7 was selected for further analysis. Typically, cells were treated with mitQ7 at 10^4^ cells/cm^2^ of a culture vessel (25 cm^2^ culture flask or 6-well plate).

### 2.7. MitQ7 Uptake

MitQ7 uptake was analyzed using imaging flow cytometry (Amnis^®^ FlowSight^®^ imaging flow cytometer) and IDEAS software (version 6.2.187.0, Luminex Corporation, Austin, TX, USA). The samples were treated with 1 µM mitQ7 for 24 h, and two parameters were applied, namely Normalized Frequency and Intensity_MC_Ch02 (excitation—488 nm, filter 505–560 nm). Results are presented as histograms.

### 2.8. Cell Cycle

Upon stimulation with 1 µM mitQ7 for 24 h, the DNA content-based analysis of cell cycle was performed using a Muse^®^ Cell Analyzer and a Muse^®^ Cell Cycle Assay Kit according to the manufacturer’s instructions (Luminex Corporation, Austin, TX, USA) [28]. Results are presented as histograms.

### 2.9. Apoptosis

Upon stimulation with 1 µM mitQ7 for 24 h, the externalization of phosphatidylserine (a marker of apoptotic cell death) was analyzed using the Muse^®^ Cell Analyzer and Muse^®^ Annexin V and Dead Cell Assay Kit (Luminex Corporation, Austin, TX, USA) [28]. Dual staining was performed, namely Annexin V and 7-AAD staining, and four subpopulations were identified: live cells (negative for both stainings), early apoptotic cells (Annexin V-positive), late apoptotic cells (positive for both stainings) and necrotic cells (7-AAD-positive). Results are presented as dot plots.

### 2.10. Intracellular pH

For the cells stimulated for 24 h with 1 µM mitQ7, intracellular pH was assayed using pHrodo™ Green AM Intracellular pH Indicator (P35373, Thermo Fisher Scientific, Waltham, MA, USA) as previously described [29]. Intracellular alkalization was analyzed as a decrease in fluorescent signals (relative florescence units, RFU) using a fluorescence microplate reader.

### 2.11. Imaging Cytometry

Upon stimulation with 1 µM mitQ7 for 24 h, cells were fixed and immunostained as previously described [30]. The following primary antibodies were used: anti-LDHA (1:100, PA5-27406), anti-phospho-AMPK (Thr172) (1:100, 2535), anti-NSUN4 (1:250, 720212), anti-NSUN6 (1:600, PA5-61119), anti-FOXO3a (1:200, MA5-14932), and anti-SOD1 (1:100, PA5-23245) (at 4 °C for 24 h) (Thermo Fisher Scientific, Waltham, MA, USA, Cell Signaling Technology, Danvers, MA, USA). Next, the samples were incubated with dedicated secondary antibodies conjugated with fluorochromes (for example, anti-rabbit secondary antibody conjugated with Texas Red, 1:1000, T2767, and anti-rabbit secondary antibody conjugated with FITC, 1:1000, T2765, Thermo Fisher Scientific, Waltham, MA, USA) at room temperature for 1 h. Nuclei were visualized using Hoechst 33342 staining. Lipid peroxidation-derived protein modifications were also investigated in fixed cells using a Click-iT^®^ Lipid Peroxidation Imaging Kit—Alexa Fluor^®^ 488 (C10446, Thermo Fisher Scientific, Waltham, MA, USA) as previously described [30]. Moreover, mitQ7-treated live cells were used to evaluate total ROS levels (CellROX™ Green Reagent, C10444, Thermo Fisher Scientific, Waltham, MA, USA) and the pools of mitochondrial superoxide (MitoSox^TM^ Red Indicator, M36008, Thermo Fisher Scientific, Waltham, MA, USA). Fluorescence signals were collected using a confocal imaging system IN Cell Analyzer 6500 HS (Cytiva, Marlborough, MA, USA). Quantitative analysis was performed using IN Carta software 1.14 (Cytiva, Marlborough, MA, USA). The levels of proteins and oxidative stress parameters are presented in relative fluorescence units (RFU). For some samples, cytosolic and/or nuclear fractions of proteins were studied.

### 2.12. The Analysis of mitQ7-Induced Senescence

Cancer cells were treated with 1 µM mitQ7 for 24 h. Then, the medium containing mitQ7 was removed and replaced with the fresh one and cells were left for 7 days to allow the development of the mitQ7-mediated senescence phenotype. Cell culture medium was exchanged every 2 days with a fresh portion. After 7 days, selected senescence biomarkers were analyzed in fixed cells, namely senescence-associated beta-galactosidase (SA-beta-gal) activity, the levels of cell cycle inhibitors p21 and p27, and the pools of pro-inflammatory cytokines IL-6 and IL-8 as a part of senescence-associated secretory phenotype (SASP). SA-beta-gal was assayed using the CellEvent™ Senescence Green Detection Kit (Thermo Fisher Scientific, Waltham, MA, USA) as previously described [31]. For immunofluorescence analysis, primary antibodies anti-p21 (1:800, MA5-14949), anti-p27 (1:200, PA5-27188), anti-IL-6 (1:500, ab6672) anti-IL-8 (1:500, ab154390) and dedicated secondary antibodies conjugated with fluorochromes were used (for example, anti-rabbit secondary antibody conjugated with Texas Red, 1:1000, T2767, and anti-rabbit secondary antibody conjugated with FITC, 1:1000, T2765, Thermo Fisher Scientific, Waltham, MA, USA). Fluorescence signals were analyzed using a confocal imaging system IN Cell Analyzer 6500 HS and IN Carta software 1.14 (Cytiva, Marlborough, MA, USA). The SA-beta-gal activity and the levels of p21, p27, IL-6, and IL-8 are presented in relative fluorescence units (RFU).

### 2.13. The Analysis of mitQ7-Mediated Senolytic Activity

Cancer cells were stimulated with 35 nM doxorubicin (DOX, 44583, Merck KGaA, Darmstadt, Germany) for 24 h to promote stress-induced premature senescence (SIPS). After drug removal, the cell culture was continued for 7 days with medium exchange every 48 h to exclude the effect of nutrient limitations. After activation of the doxorubicin-induced senescence program, senescent cancer cells were treated with 1 µM mitQ7 for 24 h to document whether mitQ7 may decrease the viability of senescent cancer cells (a senolytic effect). As already described, mitQ7-induced apoptosis in senescent cancer cells was analyzed using a flow cytometry and dual staining (Annexin V and 7-AAD staining, Muse^®^ Annexin V and Dead Cell Assay Kit, Luminex Corporation, Austin, TX, USA). Furthermore, the depolarization of mitochondrial transmembrane potential was also investigated using a Muse^®^ Cell Analyzer and Muse^®^ Mitopotential Assay Kit [30]. Briefly, four cell subpopulations were revealed, namely cells with intact mitochondrial membrane and 7-AAD-negative, cells with depolarized mitochondrial membrane and 7-AAD-negative, cells with depolarized mitochondrial membrane and 7-AAD-positive, and cells with intact mitochondrial membrane and 7-AAD-positive. The results are presented as dot plots.

### 2.14. The Analysis of Gene Mutation

Datasets of gene mutation status in six breast cancer cell lines were downloaded from the Dependency Map (DepMap) portal (https://depmap.org/portal/, accessed on 18 August 2023) (raw data are presented in Supplementary Material S2). The selected variants per each breast cancer cell line were then evaluated for gene enrichment in four databases—Reactome, KEGG, Panther and GO Pathways with the use of Kobas 3.0 standalone software [32]. Only pathways with a corrected *p*-value under 0.05 (FDR correction method of Benjamini and Hochberg [33]) were chosen for further graphical analysis using UpSetR software 1.4.0 [34] (https://cran.r-project.org/web/packages/UpSetR/index.html, accessed on 8 September 2023). The levels of gene mutations (total, mitochondria-related function mutation, kinase mutation) were then correlated with the MTT-based results on metabolic activity after treatment with 5 µM mitQ7. Data correlation analysis was performed using a linear correlation test (Spearman’s r). The 95% confidence intervals and the *r* and *p* values were used. Gene mutation types were also included and presented with the use of https://www.bioinformatics.com.cn as a chord diagram (accessed on 20 October 2023). For this purpose, genes associated with pathways related to kinase mitochondrial related processes were considered.

### 2.15. Data Integration and Clustering Analysis (Heat Map)

Raw data based on imaging cytometry were used and calculated using log2 transformation and hierarchical clustering average linkage WPGMA (Genesis software, version 1.8.1) [35]. Data are presented as heat maps.

### 2.16. Statistical Analysis

The results are presented as the arithmetic mean ± standard deviation (SD) based on three independent biological replicates. For data presentation of selected experiments, box and whisker plots with median, lowest, and highest values are also used. MitQ7-treated and non-treated cell cultures were compared using one-way analysis of variance (ANOVA) and Dunnett’s multiple comparison test. *p* values of less than 0.05 were considered as statistically significant. Statistical analysis was conducted using the GraphPad Prism 8 software.

## 3. Results and Discussion

Mitochondrial molecular machinery involves hundreds of proteins that participate in cellular metabolism, with cellular respiration and energy production as the most recognized function of mitochondria, but other fundamental processes are also facilitated, such as calcium homeostasis, glucogenesis, thermogenesis, urea and TCA cycle, heme biosynthesis, and apoptosis. Specific sites of electron transport chain complexes in mitochondria are the main intracellular source of reactive oxygen species (ROS); therefore, the redox status of these organelles is critical to controlling cell life and death, and redox imbalance has been implicated in a wide range of human pathologies, including cancer, neurological and cardiovascular diseases [36,37,38]. In this context, mitochondrial therapeutics, particularly those involving mitochondria-targeted drugs (and antioxidants), were intensively explored [7,39,40]. After the rapid development of mitochondria-targeted antioxidants, expected research goes beyond the direct protection of mitochondria against ROS and extends to the exploration of the activity and role of mito-drugs in mitochondria-controlled processes. This goal is becoming a next front-line area, and in our research, we selected three mitochondriotropic derivatives of quercetin, which are expected to have limited or even blocked antioxidant/pro-oxidant activity, in order to reveal their ability to modulate processes other than those mediated by ROS.

### 3.1. Synthesis of Quercetin Derivatives

Based on our previous observations that blocking all hydroxy groups in quercetin greatly enhances its senolytic activity during etoposide-induced senescence in MDA-MB-231 cells [29], we decided to additionally increase the lipophilicity of quercetin and convert it into a mitochondrial-targeted molecule. The development and functional testing of molecules that specifically target mitochondria involve several strategies, including molecules conjugated with lipophilic cations (e.g., triphenylphosphonium) or rhodamine, conjugates of plant alkaloids, amino-acid- and peptide-based compounds, and liposomes [40,41]. Among all these strategies, a covalent link of a lipophilic cation such as an alkyltriphenylphosphonium (TPP^+^) moiety is suggested to be the most effective way of delivering the small molecular drug to mitochondria, and depending on the cell and transmembrane potential, the final accumulation of this mito-compound can be 1000-fold higher than the concentration of the non-derived compound [7]. The attachment of the TPP^+^ moiety to polyhydroxyphenols significantly improves their bioavailability and enhances therapeutic effects; see the Introduction section. Thus, in the present study, three derivatives were obtained with all blocked hydroxy groups. As can be seen in Scheme 1, the regioisomeric derivatives mitQ3, mitQ5, and mitQ7 can be obtained with the same procedures, but the site of covalent attachment of TPP^+^ in the final product depends on the order of the operations. Each synthesis started with the selective protection/deprotection of selected hydroxy groups, which was followed by alkylation of the remaining free OH group with 1-bromo-4-chlorobutane and subsequent substitution of chlorine with iodine. Finally, the reaction of the iodo-derivative with Ph_3_P allowed us to introduce a triphenylphosphonium cation. Satisfactory yields were obtained in all steps; see Scheme 1. Products of each step and final products were identified with ^1^H and ^13^C NMR spectroscopy and mass spectrometry; see Supplementary Material S1 for details (Schemes S1–S3, and Figures S1–S25). Importantly, each derivative obtained was prepared in two independent multi-step syntheses, which allowed us to have two batches of mitQ derivatives to be used in the biological evaluation.

### 3.2. Antioxidant Activity of Quercetin Derivatives and Their Interactions with DMPC Liposomes

**Figure 1:**
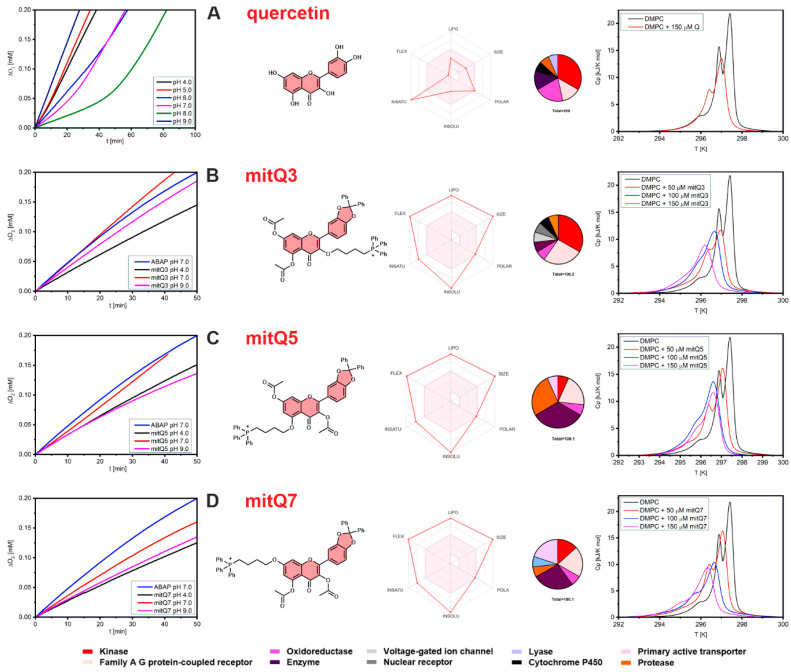
(**Left column**): The plots of oxygen uptake during peroxidation of 2.7 mM methyl linoleate in 10 mM DMPC liposomes initiated with 10 mM ABAP; see the Experimental section for details. Lines designed as ABAP represent peroxidation without additives, at pH = 7.0. (**Central**): Structural formulas of the studied compounds accompanied by ‘*Bioavailability Radar*’ plots presenting the six physicochemical parameters for each compound, namely lipophilicity (LIPO), molecular weight (SIZE), polarity (POLAR), solubility (INSOLU), rotatable bond flexibility (FLEX) and saturation (INSATU), and with circle charts displaying the affinity of each compound to enzymes and proteins as indicated in the legend at the bottom. (**Right column**): DSC curves obtained for DMPC liposomes containing 50, 100, and 150 μM of quercetin, mitQ3, mitQ5, and mitQ7. Black line represents pure DMPC liposomes without additives. Each row (**A**–**D**) represents the data for quercetin, mitQ3, mitQ5, and mitQ7, respectively.

In contrast to the series of mitochondria-targeted polyphenolic antioxidants (ubiquinone Q_10_, resveratrol, curcumin, quercetin, caffeic acid) with free hydroxy groups (unblocked) and capable of reacting directly to reduce ROS via hydrogen atom transfer within mitochondria [7,40], our goal was to obtain mito-targeted compounds without free hydroxy groups. Therefore, we did not expect a significant antioxidant effect for any mitQ in the liposomal system at pH 4–9. The kinetic plots presented in Figure 1A (left column) and Figure S26 confirm our expectations: unsubstituted quercetin (Q) exhibits no activity at pH < 6.0, weak antioxidant /retardation activity at pH 6.0, and for increasing pH, it becomes a relatively good inhibitor of peroxidation with induction times (i.e., the period when the chain reaction mediated by peroxyl radicals is suppressed) τ = 16.9 ± 1.2 min at pH 7.0 and τ = 46.8 ± 3.2 min at 8.0, which corresponds to rate constants for reaction of Q with alkylperoxyl radicals k_inh_ = (0.8 ± 0.3) × 10^4^ M^−1^s^−1^ (pH 7.0) and (0.4 ± 0.1) × 10^4^ M^−1^s^−1^ (at pH 7.0), see Table S1, with the rates of inhibited peroxidation and calculated *k*_inh_ values. Within the entire pH range, none of the three quercetin derivatives can trap peroxyl radicals with a kinetically significant rate. Thus, an induction period was detected; see Figure 1 (left column, B–D). The observed fluctuation of the peroxidation rates in the presence of mitQ is caused by a small variation of rates of non-inhibited peroxidation, which is slightly dependent on pH (non-inhibited process is presented for pH 7.0 for clarity). The observed results also provide a useful information that regardless of pH, in the liposomal system, the mitochondrial derivatives are stable and their blocked OH groups do not decompose into free hydroxy groups, which would convert mitQ into a more active radical trapping agent. We hope the results obtained in the artificial liposomal system can be extended to cellular systems, because deacetylation catalyzed by subcellular esterases is relatively slow, as reported for 3′,4′,5,7-tetra-acetyl-3-(4-*O*-triphenylphosphoniumbutyl) quercetin iodide (TAPQ) incubated with HCT116 human colon tumor cancer cells [16]. Much faster deacetylation was documented when TAPQ was incubated with rat liver mitochondria [17]; however, these results are partially relevant for our three derivatives, because esterases are not able to remove diphenylmethane from oxygen atoms at positions 3′ and 4′ in mitoQ3, 5, 7. Thus, the catechol moiety (a group responsible for the overall antioxidant activity of quercetin [42,43]) is still not redox-active. Thus, any substantial activity of mitQ in biological systems, which will be described in more detail, cannot be assigned to the presence of a free catechol moiety or antioxidant activity of mitQ.

The loss of the ability to trap peroxyl radicals is one of many other features expected to be changed when all polar OH groups of quercetin are transformed into non-polar aryl or acyl moieties. We use SwissADME software [44] (http://www.swissadme.ch/termsofuse.php, accessed on 11 July 2023) to predict several descriptors relevant to the pharmacological profile of small molecules. Figure 1 (central panel) presents *Bioavailability Radar* for each studied compound, i.e., a graphical representation of six physicochemical properties: lipophilicity, size, polarity, solubility, flexibility, and saturation calculated for quercetin and its derivatives. Taken together, these plots provide a clear picture of the dramatically changed character of quercetin after transformation into mitQ (the calculated values of these parameters and the pharmacokinetic parameters are listed in Figures S27–S30). The calculations of the partition coefficient log*P*_o/w_ (as an average value obtained from five predictive models) gave a reasonable value of 1.23 for quercetin (in agreement with experimental 1.82 [45]) but completely failed for mitQ derivatives, because unreasonably high values were obtained (9.03 for mitQ5 and mitQ7 and 9.49 for mitQ3), while the values expected for such compounds should be approximately 2 to 3 [46,47]. We include this information to warn other researchers that current versions of software, such as SwissADME (this work) or pkCSM, also used for log*P* calculations [48], could be useless for species containing large lipophilic cations, although they can be used to compare properties within the series of similar compounds. The problem we noticed here needs to be studied more carefully, and the experimental values of broader series of mitochondrial derivatives will be the subject of another publication. Other predicted parameters, which are helpful for assessing ADME parameters: absorption, distribution, metabolism, and excretion, are presented in Figures S27–S30; however, these should also be considered with careful criticism.

The pharmacokinetic data modeled with SwissADME are consistent with the experimental observations of phase behavior of the liposomal systems loaded with quercetin or with mito-derivatives. The effect of these compounds on the thermotropic behavior of the LUV suspension of DMPC was monitored with DSC and presented in Figure 1 (right column) as temperature-dependent isobaric molar heat capacities, C_p_, recorded for buffered suspensions of DMPC. The peaks indicate thermal effects during the phase transition from an ordered (gel) to disordered (fluid) phase. A careful comparison of the thermal behavior of LUVs indicates that all four compounds interact with DMPC and affect the transition parameters (the width and position of the peak). Quercetin (even at a concentration as high as 150 μM) did not substantially change the shape and position of the transition peaks. On the contrary, DMPC vesicles loaded with mitQ3, 5, and 7 are clearly different from pure DMPC with stronger broadening of the peak (Figure 1) and with a slight decrease in transition temperatures and molar enthalpies of phase transition (data collected in Table S2). An asymmetric broadening of transition peaks observed in the presence of mitQ could be caused by different affinity for DMPC before and after the phase transition with the fluid phase attracting more mitQ than the gel phase [49]. Another interesting effect is that the peak broadening and thermal effect recorded for mitQ3 at a concentration of 50 μM are significantly higher than for mitQ5 and mitQ7 (see Figure 1 and Table S2), indicating a stronger affinity of mitQ3 to DMPC membranes than for two other mitQ. This different behavior of mitQ3 agrees with the higher lipophilicity predicted by SwissADME (regardless of the critics expressed above, the software can be used for a comparison of compounds within the family of structurally relevant compounds), and it can also be explained by the slightly different shape of the molecule (mitQ5 and mitQ7 are more linear, whereas mitQ3 has a T-shaped structure).

Both kinetic and calorimetric results lead to the conclusion that the absence of free hydroxy groups makes the mitochondria-targeted derivatives of quercetin not active toward peroxyl radicals, although all three derivatives exhibit much stronger interactions with DMPC lipid bilayers compared to the interaction of DMPC with unsubstituted quercetin.

SwissTargetPrediction-based in silico analysis (http://www.swisstargetprediction.ch/, accessed on 11 July 2023) revealed putative protein targets for quercetin and its three derivatives (Figure 1, central column). Several protein categories were considered such as enzymes, kinases, proteases, oxidoreductases, etc. Three modifications affected the pattern of protein interactions compared to quercetin (Figure 1, central column). MitQ3 is able to interact with more proteins of family A G protein-coupled receptors, mitQ5 with proteases, and mitQ7 with primary active transporters compared to quercetin, respectively.

### 3.3. MitQ7-Mediated Cytotoxic and Cytostatic Effects in Breast Cancer Cells with Different Mutation and Receptor Status

The initial research on the impact of mitochondriotropic derivatives of quercetin on rat liver mitochondria, C-26 mouse colon cancer cells and human Jurkat T cell leukemia cells [16,17,18,19] reviewed recently by Biasutto et al. [22] inspired us to analyze the anticancer effects of mitQ3, 5, and 7 in six phenotypically diverse breast cancer cell lines, namely ER-positive MCF-7, HER2-positive SK-BR-3 and four triple-negative (TNBC) MDA-MB-231, MDA-MB-468, BT-20, and Hs 578T cells.

First, the concentration and quercetin derivative were selected for further studies based on the MTT test (Figure S31). This is an initial assay that allows us to monitor changes in metabolic activity of the cells upon stimulation with test agents without providing direct evidence on cytotoxicity (cell death) or cytostatic effects (inhibition of cell proliferation). We used two cell densities (5 × 10^4^ and 1 × 10^5^ cells per mL) and two glucose concentrations in the culture medium—4.5 g/L (≈25 mM) ”high glucose” (HG) which is optimal for most cancer cells and is used routinely in cancer cell culture in vitro experiments, and 1.0 g/L (≈5.5 mM) ”low glucose” (LG); the latter can be considered suboptimal and promotes energetic stress in cancer cells in vitro but reflects the situation in vivo: that is, healthy (normal) fasting blood glucose level (Figure S31). Recent claims of limited cell proliferation and motility following glucose deprivation resulted in the hypothesis that this kind of energetic stress might increase the antiproliferative activity of anticancer drugs, such as paclitaxel and doxorubicin, in breast cancer cells [21]. Here, the effects of mitQ3, 5, and 7 were compared with the action of unmodified Q at concentrations of 0.1, 1, 5, and 10 µM (Figure S31). Proliferatively active human fibroblasts were also used to document the action against normal cells (Figure S31). Within the whole concentration range, quercetin was relatively ineffective against normal and cancer cells compared to the effects of mitochondriotropic derivatives (Figure S31). For further analysis, we selected mitQ7 as the most promising, because at a concentration of 1.0 µM concentration, this compound was active against most breast cancer cell lines (this concentration was approximately the concentration of IC50 when MCF-7 and MDA-MB-231 cells were cultured at 5 × 10^4^ cells per mL in a “high-glucose” medium). Furthermore, mitQ7 was significantly less effective against normal cells (a decrease of approximately 10% of metabolic activity compared to untreated conditions) (Figure S31). In general, for lower cell and glucose concentrations, cancer cells were more sensitive to mitochondria-targeted quercetin derivatives (Figure S31). However, for further analysis, we selected a higher concentration of cells, as it is more relevant to in vivo conditions under which mammary epithelial cells are tightly packed together.

**Figure 2:**
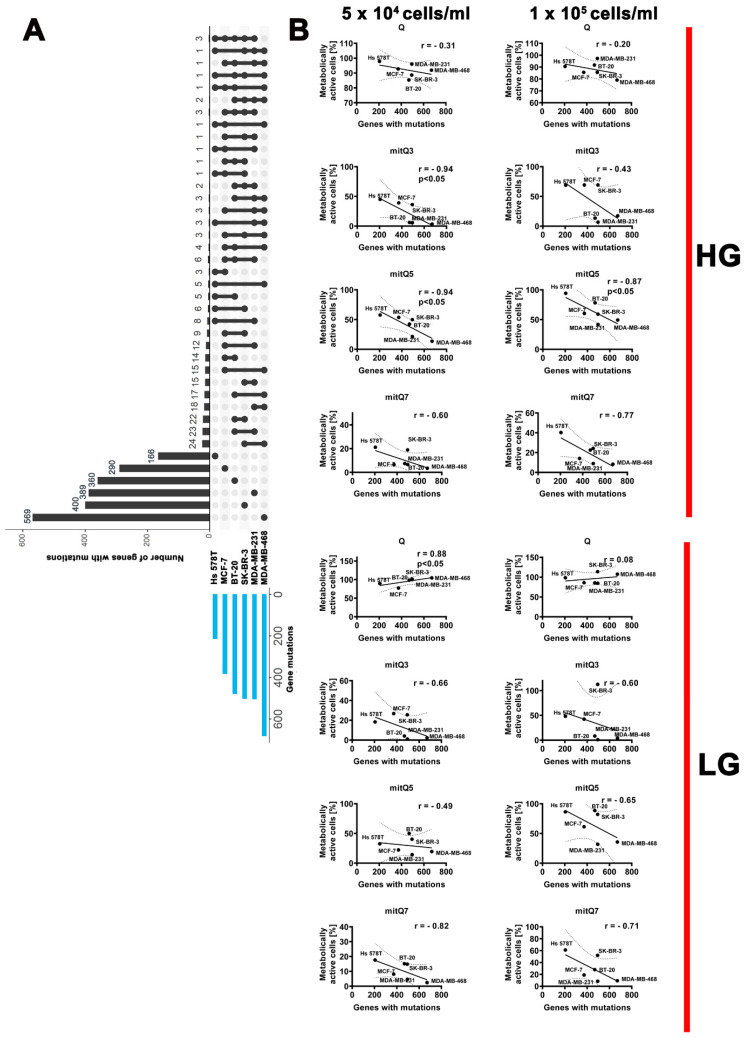
Gene mutation status (**A**) and correlation analysis between the metabolic activity (MTT-based data) and the number of total gene mutations (**B**) in six breast cancer cell lines used in the study. (**A**) Gene mutation raw data were acquired from DepMap portal (https://depmap.org/portal/, accessed on 18 August 2023). Set intersections in a matrix layout were visualized using the UpSet plot. Total, shared and unique gene mutations across six breast cancer cell lines are shown. Blue bars in the *y*-axis represent the total number of gene mutations in each cell line. Black bars in the *x*-axis represent the number of mutations shared across cell lines connected by the black dots in the body of the plot. (**B**) Correlation analysis of the data was performed using a linear correlation (Spearman’s r) test. The 95% confidence interval, *r* and *p* values are shown. HG, high-glucose DMEM (4.5 g/L); LG, low-glucose DMEM (1 g/L).

Furthermore, we have correlated MTT-based results on changes in metabolic activity after stimulation with 5 µM Q and mitQ3, 5 and 7 under different cell densities and glucose concentrations with the number of gene mutations per breast cancer cell line (Figure 2). Data on gene mutations in six breast cancer cell lines used in this study were acquired from the Dependency Map (DepMap) portal (https://depmap.org/portal/, accessed on 18 August 2023; raw data are also presented in Supplementary Material S2). An increased sensitivity to mito-quercetin, especially mitQ3 and 5, was observed in breast cancer cell lines with more total gene mutations compared to other cell lines: for example, in MDA-MB-468 cells (the highest number of gene mutations and unique gene mutations) compared to Hs 578T cells (the lowest number of gene mutations and unique gene mutations) (Figure 2, Supplementary Material S3, Table S3). The sensitivity to mitQ7 was more pronounced in breast cancer cells with more gene mutations in genes involved in mitochondrial function, for example in MDA-MB-468 cells (Figure S32 and Supplementary Material S3, Table S4). Surprisingly, glucose-limited conditions lowered the sensitivity to the treatment with 5 µM Q, but not mito-quercetin, of breast cancer cells with higher level of gene mutations (e.g., MDA-MB-468 cells) compared to breast cancer cells with lower level of gene mutations (e.g., Hs 578T cells) (Figure S32 and Supplementary Material S3, Table S4). This observation was the most pronounced when considering gene mutations in genes that encode protein kinases (Figure S33 and Supplementary Material S3, Table S5).

**Figure 3:**
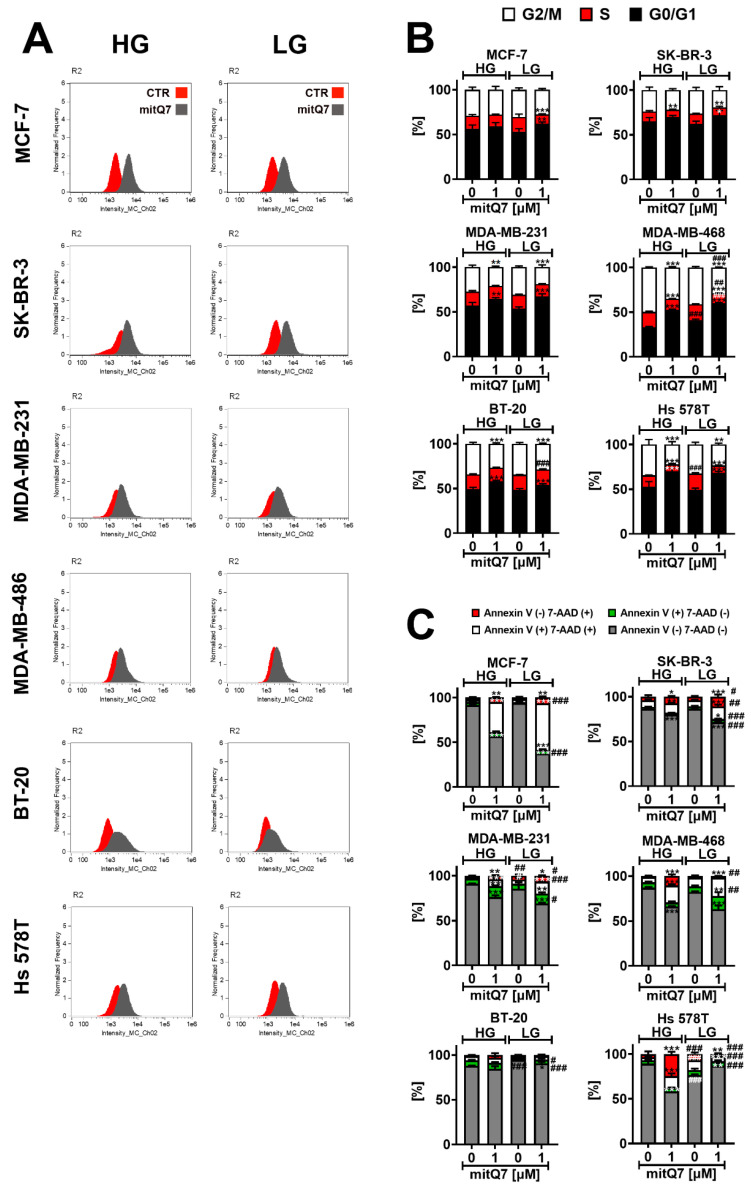
MitQ7 uptake (**A**), mitQ7-mediated changes in the phases of cell cycle (**B**) and mitQ7-induced apoptosis (**C**) in six breast cancer cell lines: namely, ER-positive MCF-7, HER2-positive SK-BR-3 and four triple-negative (TNBC) MDA-MB-231, MDA-MB-468, BT-20, and Hs 578T cells. Breast cancer cells were treated with 1 µM mitQ7 for 24 h. (**A**) MitQ7 uptake was analyzed using imaging flow cytometry and dedicated software. Two parameters were considered, namely Normalized Frequency and Intensity_MC_Ch02. Representative histograms are presented. (**B**) DNA content-based analysis of cell cycle using flow cytometry and dedicated DNA staining. Bars indicate SD, n = 3, *** *p* < 0.001, ** *p* < 0.01, * *p* < 0.05 compared to HG or LG untreated control (ANOVA and Dunnett’s a posteriori test), ^###^ *p* < 0.001, ^##^ *p* < 0.01 compared to HG corresponding conditions (ANOVA and Dunnett’s a posteriori test). (**C**) Apoptosis was assayed using flow cytometry and Annexin V staining (apoptotic marker) and 7-AAD staining (necrotic marker). Four subpopulations were revealed, namely live cells (dual staining-negative), early apoptotic cells (Annexin V-positive), late apoptotic cells (dual staining-positive), and necrotic cells (7-AAD-positive). Bars indicate SD, n = 3, *** *p* < 0.001, ** *p* < 0.01, * *p* < 0.05 compared to HG or LG untreated control (ANOVA and Dunnett’s a posteriori test), ^###^ *p* < 0.001, ^##^ *p* < 0.01, ^#^ *p* < 0.05 compared to HG corresponding conditions (ANOVA and Dunnett’s a posteriori test). HG, high-glucose DMEM (4.5 g/L); LG, low-glucose DMEM (1 g/L).

Second, the uptake of mitQ7 was analyzed by the means of imaging flow cytometry (Figure 3A). MitQ7 was taken up by six breast cancer cell lines; however, the most efficient uptake was revealed in the case of MCF-7 cells (Figure 3A). In SK-BR-3 cells, LG conditions enhanced mitQ7 uptake (Figure 3A).

Third, we were interested in whether mitQ7-mediated changes in metabolic activity (MTT test, Figure S31) were associated with changes in cell cycle progression or cytotoxic effects (Figure 3B,C and Figures S34 and S35). The DNA content-based analysis of the cell cycle revealed that except for MCF-7 cells cultured under HG conditions, mitQ7 treatment resulted in G1 phase cell cycle arrest in breast cancer cells (Figure 3B). In general, LG conditions did not enhance mitQ7-mediated effects on cell cycle inhibition (Figure 3B). Except of BT-20 cells, mitQ7 promoted apoptosis in breast cancer cells, as judged by the analysis of phosphatidylserine externalization (Annexin V staining, Figure 3C). The differences in the response to mitQ7 stimulation might be due to the genetic background of the individual cell lines. In the case of the TNBC cell lines, the bioinformatics analysis used in this study showed that BT-20 cells have missense type mutations in the following genes, which are not present in the other TNBCs lines used in the present study: *MT-ATP6*, *PPARGC1A*, *RARS2*, *CBLB*, *SOD1*, *ALDH1L2*, *MPST*, *PTPN1*, *NCSTN*, *NLRX1*, *NAPG*, *HEMK1*, *ATPAF2*, *RAP1GDS1*, *ESR2*, *DDX21*, *TTC19*, *NEFL*, *INF2* (Supplementary Material S3, Table S4). These genes are involved in the mitochondrial function; thus, it is likely that corresponding mutations may modulate the response to mitQ7 stimulation. Of course, more mechanistic studies are needed to validate this suggestion. Furthermore, in mitQ7-treated Hs 578T cells, a necrosis-based cell death morphotype was observed (Figure 3C). This observation may be due to missense gene mutations in the *NLRC4* gene (NLR family CARD domain containing 4) and the *CARD8* gene (caspase recruitment domain family member 8) in Hs 578T cells (Supplementary Material S2) that may affect apoptotic cell death. More studies are needed to reveal whether mitQ7-induced necrosis in Hs 578T cells is of an unregulated (classical necrosis) or regulated nature (necroptosis or other regulated cell death modes with morphological features of necrosis). MitQ7-induced apoptosis was correlated with intracellular alkalization in five breast cancer cell lines, as presented in Figure S36. The most susceptible to mitQ7 treatment were MCF-7 cells, and LG conditions potentiated the apoptotic activity of mitQ7 in MCF-7, SK-BR-3, MDA-MB-231 and MDA-MB-468 cells (Figure 3C). On the contrary, LG conditions limited mitQ7-induced cytotoxicity in Hs 578T cells, here necrotic cell death (Figure 3C). Surprisingly, the low glucose level is able to promote apoptosis in Hs 578T cells compared to the HG conditions, as judged by an increase in the levels of Annexin V-positive cells (Figure 3C). The most pronounced cytotoxicity of mitQ7 in MCF-7 cells can be correlated with the most efficient uptake of mitQ7 in MCF-7 cells compared to other breast cancer cells (Figure 3B,C).

### 3.4. MitQ7 Treatment is Accompanied by Oxidative Stress in Breast Cancer Cells

**Figure 4:**
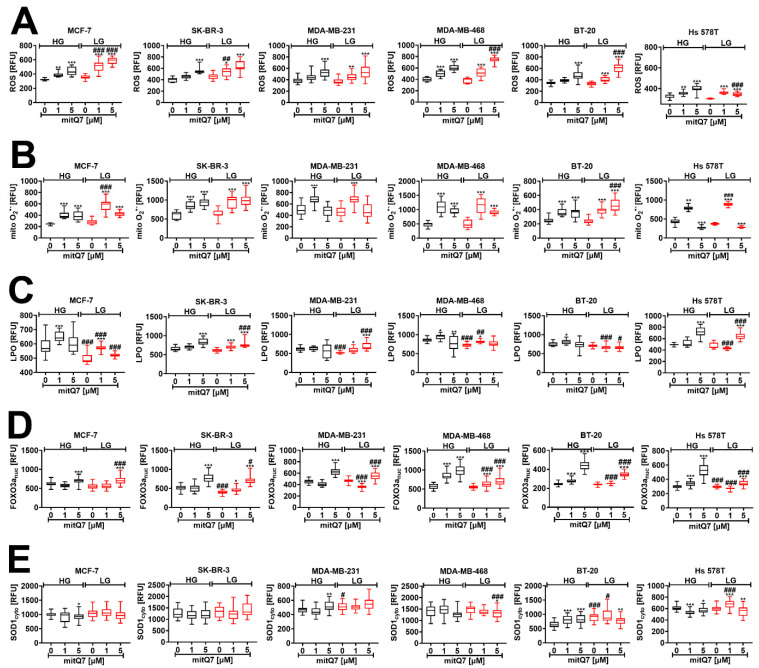
MitQ7-stimulated oxidative stress and related responses in six breast cancer cell lines, namely, ER-positive MCF-7, HER2-positive SK-BR-3 and four triple-negative (TNBC) MDA-MB-231, MDA-MB-468, BT-20, and Hs 578T cells. Breast cancer cells were treated with 1 µM or 5 µM mitQ7 for 24 h. (**A**–**E**) Imaging cytometry-based analysis was applied. (**A**) Total ROS levels were assessed using CellROX™ Green Reagent. (**B**) Mitochondrial superoxide levels were analyzed using MitoSox^TM^ Red Indicator. (**C**) Lipid peroxidation-derived protein modifications were revealed using a Click-iT^®^ Lipid Peroxidation Imaging Kit. (**D**) FOXO3a activation (increased levels of nuclear FOXO3a) was analyzed using dedicated anti-FOXO3a antibody. (**E**) The levels of superoxide dismutase SOD1 were investigated using dedicated anti-SOD1 antibody. (**A**–**E**). Data are presented as relative fluorescence units (RFU). Box and whisker plots are shown, n = 3, *** *p* < 0.001, ** *p* < 0.01, * *p* < 0.05 compared to HG or LG untreated control (ANOVA and Dunnett’s a posteriori test), ^###^ *p* < 0.001, ^##^ *p* < 0.01, ^#^ *p* < 0.05 compared to HG corresponding conditions (ANOVA and Dunnett’s a posteriori test). HG, high-glucose DMEM (4.5 g/L); LG, low-glucose DMEM (1 g/L).

To analyze mitQ7-mediated redox imbalance, we have considered an apoptosis-inducing concentration of mitQ7, namely 1 µM (Figure 3C) and also 5 µM concentration that stimulated massive changes in metabolic activity (MTT test, Figure S31). This approach would allow documenting concentration-dependent changes in oxidative stress parameters (Figure 4). Indeed, in most cases, 5 µM mitQ7 promoted a higher production of total ROS and mitochondrial superoxide compared to 1 µM mitQ7 (Figure 4A,B). For MCF-7 cells treated with 1 µM mitQ7, LG conditions increased ROS and mitochondrial superoxide levels, suggesting increased mitQ7 cytotoxicity (Figure 3C and Figure 4A,B). MitQ7-induced lipid peroxidation was relatively less evident in treated cells; however, in 1 µM mitQ7-stimulated MCF-7 cells at HG conditions, an increase in lipid peroxidation was noticed (Figure 4C). An activation of the forkhead box protein O3 (FOXO3a, elevated levels of the nuclear fraction of FOXO3a), a transcription factor that regulates the response to oxidative stress [50], was observed mainly when 5 µM mitQ7 was used (Figure 4D). Except for BT-20 cells, 1 µM mitQ7 treatment did not change the levels of superoxide dismutase SOD1 in breast cancer cells (Figure 4E). Perhaps SOD1-mediated adaptive response after mitQ7 stimulation in BT-20 cells may contribute to cytoprotective effects in mitQ7-treated BT-20, as evidenced by attenuated apoptotic cell death (Figure 3C and Figure 4E). Mitochondriotropic quercetin derivatives also promoted the increase in superoxide levels in HepG2 human hepatoma cells [17] and Jurkat leukemia cells [18]. Pegylated superoxide dismutase (PEG-SOD) and pegylated catalase (PEG-CAT) protected against mito-quercetin-induced apoptosis in Jurkat cells, suggesting that oxidative stress might participate in the mechanism of mito-quercetin-mediated cell death [19]. Of course, we are aware that in our experimental settings, mitQ7 promoted oxidative stress in an indirect manner, as the catechol moiety responsible for redox activity was blocked in mitQ7 in contrast to previously obtained Q derivatives [17,18]. Perhaps mitQ7 may interact with intracellular antioxidants, lowering their activity and proteins involved in the regulation of metabolic pathways or modulating the activity of redox-sensitive signaling pathways. However, such assumptions require further elucidation. We also found that the rate of mitochondrial superoxide production upon mitQ7 stimulation can be correlated with the levels of gene mutations in genes involved in mitochondrial function (Figure S32 and Table S4). Hs 578T and MDA-MB-231 cells with lower levels of gene mutations in genes involved in mitochondrial function were characterized by limited mitochondrial superoxide production after 5 µM mitQ7 treatment compared to MDA-MB-468 cells with elevated mitochondrial superoxide production after 5 µM mitQ7 treatment and higher levels of gene mutations in genes involved in mitochondrial function that also differentially modulated metabolic activity (MTT-based results) in these two breast cancer cell lines (Figure S32 and Table S4). Perhaps a lower rate of mitochondrial superoxide production after mitQ7 stimulation in Hs 578T and MDA-MB-231 cells might be associated with gene mutations in the genes *ANK2* (ankyrin-2 that regulates the localization and stabilization of the membrane of ion transporters and ion channels) and *SYNE2* (nesperin-2 that maintains the structural integrity of the nucleus) genes that were not noticed in other breast cancer cell lines (Table S4). More studies are needed to validate these suggestions.

### 3.5. MitQ7 Promoted Energetic Stress in Breast Cancer Cells

**Figure 5:**
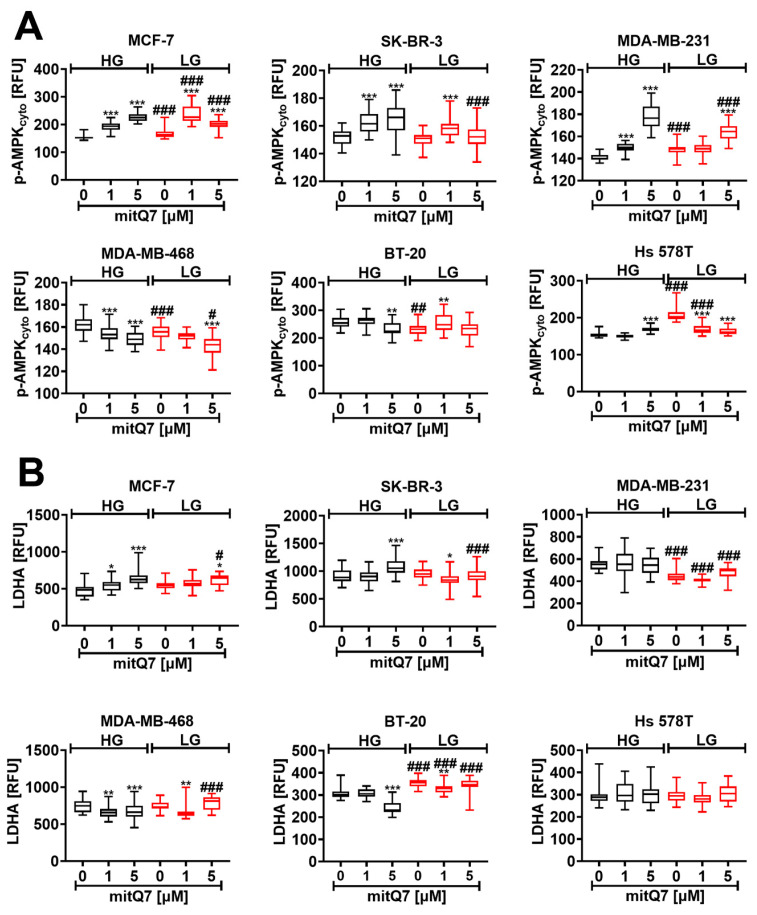
MitQ7-mediated activation of AMPK (**A**) and changes in lactate dehydrogenase (LDHA) levels (**B**) in six breast cancer cell lines, namely, ER-positive MCF-7, HER2-positive SK-BR-3 and four triple-negative (TNBC) MDA-MB-231, MDA-MB-468, BT-20, and Hs 578T cells. Breast cancer cells were treated with 1 µM or 5 µM mitQ7 for 24 h. (**A**,**B**) Imaging cytometry-based analysis was applied. (**A**) Phosphorylation status of AMPK was studied using dedicated anti-phospho-AMPK antibody. (**B**) The levels of LDHA were analyzed using dedicated anti-LDHA antibody. (**A**,**B**) Data are presented as relative fluorescence units (RFU). Box and whisker plots are shown, n = 3, *** *p* < 0.001, ** *p* < 0.01, * *p* < 0.05 compared to HG or LG untreated control (ANOVA and Dunnett’s a posteriori test), ^###^ *p* < 0.001, ^##^ *p* < 0.01, ^#^ *p* < 0.05 compared to HG corresponding conditions (ANOVA and Dunnett’s a posteriori test). HG, high-glucose DMEM (4.5 g/L); LG, low-glucose DMEM (1 g/L).

Next, we tested whether mitQ7-mediated cytotoxicity may be associated with energetic stress in breast cancer cells. The activation of AMPK was applied as a marker of energetic stress, and as expected, increased levels of phosphorylated AMPK were observed at LG conditions in MCF-7, MDA-MB-231 and Hs 578T cells (Figure 5A). We speculate that the massive induction of necrotic cell death in Hs 578T cells at LG conditions (Figure 3C) may be due to elevated energetic stress in these cells compared to other breast cancer cell lines (Figure 5A). Treatment with 1 µM mitQ7 resulted in the activation of AMPK in MCF-7, SK-BR-3, and MDA-MB-231 cells (Figure 5A), and activation was much stronger in MCF-7 cells under LG conditions, which may explain the increased cytotoxicity of mitQ7 against these cells at low glucose (Figure 3C and Figure 5A). In general, AMPK activation is an adaptive response to glucose deprivation based on metabolic reprograming to support catabolic pathways (glucose uptake, glycolysis, fatty acid oxidation, autophagy) and limit anabolic pathways (protein, fatty acid, and glycogen synthesis) [51,52]. On the other hand, AMPK was reported to be a negative regulator of aerobic glycolysis, so called the Warburg effect—the preferential fermentation of glucose-derived pyruvate to lactate despite available oxygen, suppressing tumor growth in vivo [53]. Thus, we were then interested if the mitQ7-mediated activation of AMPK may be accompanied by the changes in the levels of lactate dehydrogenase (LDHA) (Figure 5B). Indeed, elevated levels of LDHA were observed in MCF-7 cells treated with mitQ7, but this effect was not cytoprotective, because massive apoptosis was induced under such experimental conditions (Figure 3C). Alkaline intracellular pH has previously been reported to contribute to AMPK activation and adaptive stress responses [54]. In our experimental settings, alkalization (Figure S36) was also associated with energetic stress mediated by AMPK activation in four breast cancer cell lines (Figure 5A). On the contrary, in mitQ7-treated BT-20 cells, no alkalization was observed, and no subsequent activation of AMPK was noticed (Figure S36 and Figure 5A). To our knowledge, there is no information on mito-quercetin-mediated energetic stress, changes in AMPK activity, and related cytotoxicity against breast cancer cells. In contrast, quercetin was previously established to be an inhibitor of mitochondrial F_1_F_0_-ATPase/ATP synthase that may result in decreased mitochondrial ATP production and in turn AMPK activation [12,55,56,57]. Furthermore, the anticancer activity of quercetin against cervical and breast cancer cells may be mediated by the activation of AMPK [15,58]. We have also found that mitQ7 did not promote AMPK activation in MDA-MB-468 and BT-20 cells that may be associated with gene mutations in the *NUP88*, *WDR83,* and *ADCY2* genes involved in the regulation of the activity of kinase-based pathways (Table S5).

### 3.6. MitQ7-Mediated Elevation in NSUN4 and NSUN6 Levels

**Figure 6:**
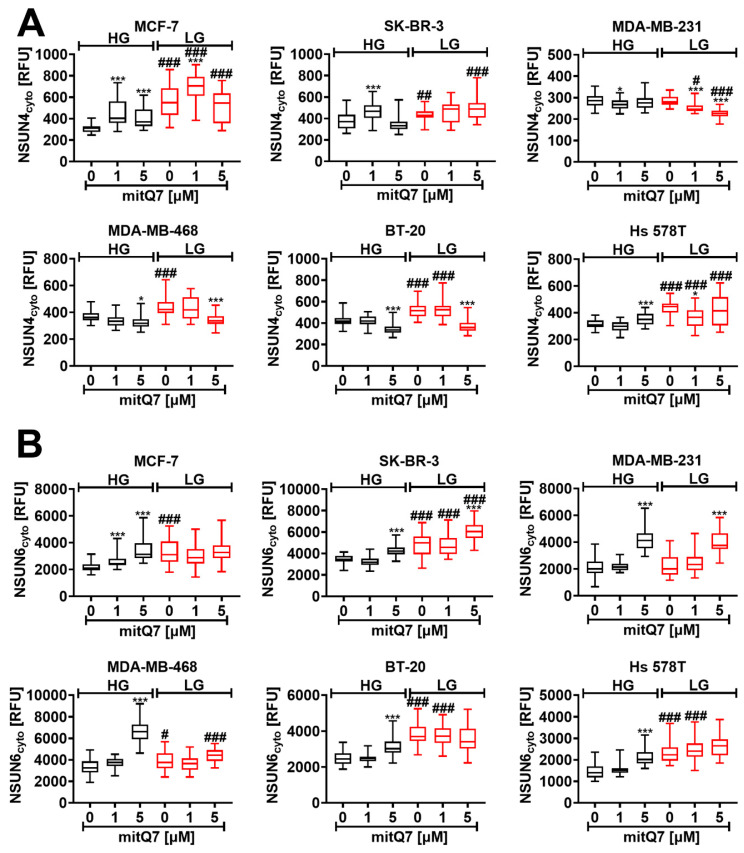
MitQ7-mediated changes in the levels of cytosolic NSUN4 (**A**) and NSUN6 (**B**) in six breast cancer cell lines, namely, ER-positive MCF-7, HER2-positive SK-BR-3 and four triple-negative (TNBC) MDA-MB-231, MDA-MB-468, BT-20, and Hs 578T cells. Breast cancer cells were treated with 1 µM or 5 µM mitQ7 for 24 h. (**A**,**B**) Imaging cytometry-based analysis was applied. (**A**) The levels of NSUN4 were investigated using dedicated anti-NSUN4 antibody. (**B**) The levels of NSUN6 were analyzed using dedicated anti-NSUN6 antibody. (**A**,**B**) Data are presented as relative fluorescence units (RFU). Box and whisker plots are shown, n = 3, *** *p* < 0.001, * *p* < 0.05 compared to HG or LG untreated control (ANOVA and Dunnett’s a posteriori test), ^###^ *p* < 0.001, ^##^ *p* < 0.01, ^#^ *p* < 0.05 compared to HG corresponding conditions (ANOVA and Dunnett’s a posteriori test). HG, high-glucose DMEM (4.5 g/L); LG, low-glucose DMEM (1 g/L).

The NOL1/NOP2/SUN domain (NSUN) family proteins NSUN1, NSUN2, NSUN3, NSUN4, NSUN5, and NSUN6 are 5-methylcytosine RNA methyltransferases with complex role(s) during tumorigenesis, cancer therapy, and related responses such as chemotherapy-induced senescence [31,59,60,61,62]. More recently, we have documented that the levels of most NSUN proteins increased during doxorubicin- and etoposide-induced senescence in four cellular models of cancer in vitro [31]. As NSUN4 is essential during mitochondrial ribosomal biogenesis [63] and NSUN6 can suppress cancer proliferation in vitro and in vivo [61], we decided to analyze the NSUN4 and NSUN6 pools after mitQ7 stimulation in the context of glucose availability (Figure 6A,B). In MCF-7 cells treated with 1 and 5 µM mitQ7, NSUN4 and NSUN6 levels were elevated, and an additional increase in the level of NSUN4 was observed when the amount of glucose was limited (Figure 6A,B). Perhaps this observation may be associated with an increase in the cytotoxicity of mitQ7 against MCF-7 cells under LG conditions. However, more studies are needed to better document such assumptions. Furthermore, when mitQ7 was used at the concentration of 5 µM, the levels of NSUN6 were increased in all six breast cancer cell lines at HG conditions (Figure 6B).

### 3.7. Limited Glucose Availability Is a Driver of Senescence in the Subpopulation of BT-20 and Hs 578T Breast Cancer Cell Survivals upon mitQ7 Stimulation

**Figure 7:**
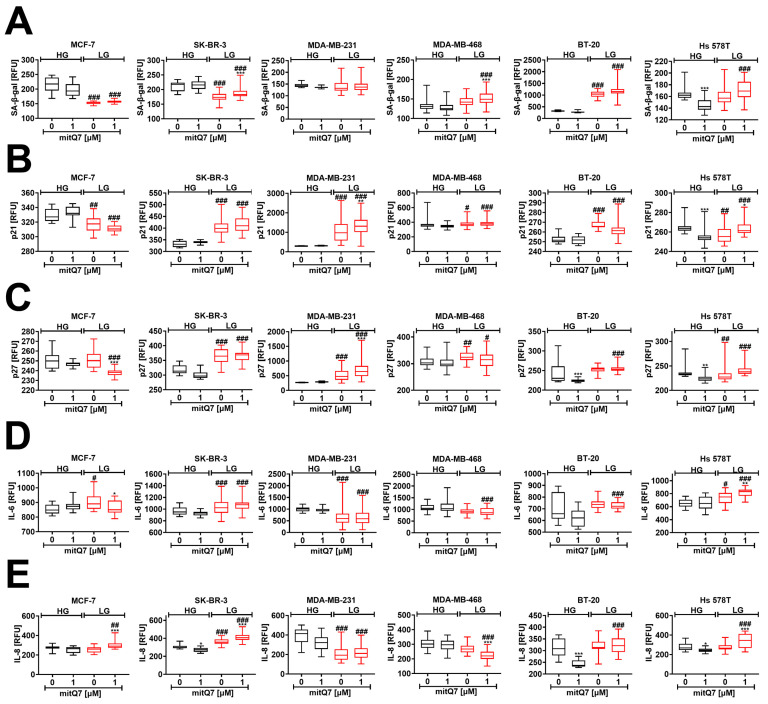
MitQ7-induced cellular senescence in six breast cancer cell lines, namely, ER-positive MCF-7, HER2-positive SK-BR-3 and four triple-negative (TNBC) MDA-MB-231, MDA-MB-468, BT-20, and Hs 578T cells. Breast cancer cells were treated with 1 µM mitQ7 for 24 h, test compound was removed and cells were cultured for 7 days after drug removal with medium exchange every 48 h. Imaging cytometry and several markers of cellular senescence were considered, namely (**A**) senescence-associated beta-galactosidase (SA-beta-gal) activity using a dedicated staining kit, (**B**) the levels of p21 using a dedicated anti-p21 antibody, (**C**) the levels of p27 using a dedicated anti-p27 antibody, (**D**) the levels of IL-6 using a dedicated anti-IL-6 antibody, (**E**) the levels of IL-8 using a dedicated anti-IL-8 antibody. (**A**–**E**) Data are presented as relative fluorescence units (RFU). Box and whisker plots are shown, n = 3, *** *p* < 0.001, ** *p* < 0.01, * *p* < 0.05 compared to HG or LG untreated control (ANOVA and Dunnett’s a posteriori test), ^###^ *p* < 0.001, ^##^ *p* < 0.01, ^#^ *p* < 0.05 compared to HG corresponding conditions (ANOVA and Dunnett’s a posteriori test). HG, high-glucose DMEM (4.5 g/L); LG, low-glucose DMEM (1 g/L).

As different breast cancer cells were characterized by different sensitivity to mitQ7 (Figure 3C), we were then interested in whether mitQ7 can promote responses other than cell death, such as cell senescence, which is a state of permanent cell cycle arrest accompanied by a number of morphological, biochemical, and molecular features such as the accumulation of cell cycle inhibitors, resistance to apoptosis and secretion of pro-inflammatory cytokines [64] (see Materials and Methods section for more details). Various senescence biomarkers were considered, namely senescence-associated-beta-galactosidase (SA-β-gal) activity, levels of cell cycle inhibitors p21 and p27, and levels of pro-inflammatory cytokines IL-6 and IL-8 as a part of the secretory phenotype associated with senescence (SASP) (Figure 7). MitQ7 did not promote SA-β-gal activity in breast cancer cells under HG conditions (Figure 7A). p21 and p27 levels also did not increase in MitQ7-treated breast cancer cells under HG conditions (Figure 7B,C). The undersupply of glucose (LG conditions) caused a decrease and an increase in SA-β-gal activity in MCF-7 and BT-20 cells, respectively (Figure 7A). Levels of SA-β-gal-positive cells were increased under LG conditions in MitQ7-treated MDA-MB-468, BT-20 and Hs 578T cells compared to the corresponding HG conditions (Figure 7A), which were also correlated with elevated levels of p21 and p27 (Figure 7B,C). Furthermore, breast cancer cell lines were characterized by different basal SA-β-gal activity at HG conditions (Figure 7A). For example, SA-β-gal activity was at least two times higher in BT-20 cells than in other cells under HG conditions (Figure 7A). It is widely accepted that some cancer cells may have high SA-β-gal activity in a non-senescence state, and this marker may have limited applications in senescence research. However, SA-β-gal activity increased approximately three times in BT-20 cells under LG conditions compared to HG conditions, which may also reflect the highest increase in SA-β-gal activity after mitQ7 stimulation under LG conditions compared to MDA-MB-468 and Hs 578T cells (Figure 7A). The susceptibility of BT-20 cells to activate the senescence program under LG conditions may be due to the different genetic background in the breast cancer cell lines used in this study. However, more studies are needed to provide an explanation of high basal SA-β-gal activity in BT-20 cells at HG conditions and LG-induced elevation in SA-β-gal activity. MitQ7 did not stimulate the production of IL-6 and IL-8 in breast cancer cells at HG conditions (Figure 7D,E). In some experimental settings, a low concentration of glucose promoted the levels of pro-inflammatory cytokines (Figure 7D,E). The production of IL-6 and IL-8 was also elevated in mitQ7-treated BT-20 and Hs 578T cells at LG conditions compared to the corresponding HG conditions (Figure 7D,E). One can conclude that mitQ7 did not activate the senescence program in breast cancer cells under HG conditions (Figure 7). In contrast, at LG conditions, five biomarkers of cellular senescence were positive in BT-20 and Hs 578T cells treated with mitQ7 (namely, SA-β-gal activity, elevated levels of p21 and p27, and increased production of IL-6 and IL-8). Based on these results, we can regard limited glucose availability as a driver of senescence in the remaining fraction of BT-20 and Hs 578T cell survival after mitQ7 treatment (Figure 7). Furthermore, low-glucose conditions (without mitQ7 stimulation) also resulted in a statistically significant decrease in the levels of SA-β-gal positive MCF-7 cells, and a similar, but insignificant, trend in SK-BR-3 cells (Figure 7A) that may be associated with gene mutations in the *LAMC2*, *ULK3* and *ACE* genes (Table S3). High-glucose conditions upon mitQ7 stimulation also caused a decrease in the levels of SA-β-gal-positive Hs 578T cells that was accompanied by a decrease in the levels of cell cycle inhibitors p21 and p27 and was correlated with the lowest level of total gene mutations in these cells (Figure 2). A decrease in the levels of pro-inflammatory cytokines, namely IL-6 and IL-8, was also observed in mitQ7-treated MDA-MB-231 and MDA-MB-468 cells under low-glucose conditions that may be related to gene mutations in the *PER1* gene, which is a regulator of the pro-inflammatory response (Table S3).

### 3.8. MitQ7-Mediated Senolytic Activity and Depolarization of Mitochondrial Transmembrane Potential in Drug-Induced Senescent Breast Cancer Cells

**Figure 8:**
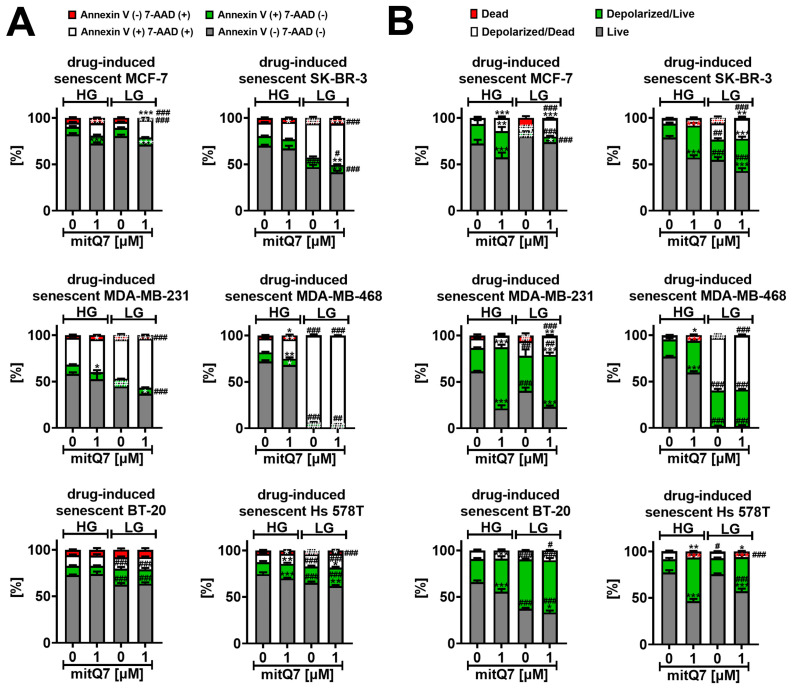
MitQ7-mediated senolytic activity (**A**) and changes in mitochondrial transmembrane potential (**B**) in doxorubicin-induced senescent breast cancer cells. MCF-7, SK-BR-3, MDA-MB-231, MDA-MB-468, BT-20, and Hs 578T cells were treated with 35 nM doxorubicin for 24 h and left for growth for 7 days after drug removal to induce senescence program. Doxorubicin-induced senescent breast cancer cells were then treated with 1 µM mitQ7 for 24 h, and apoptosis was assayed using flow cytometry and Annexin V staining (**A**), and changes in mitochondrial transmembrane potential (**B**) were revealed using flow cytometry and dedicated mitopotential probe. Bars indicate SD, n = 3, *** *p* < 0.001, ** *p* < 0.01, * *p* < 0.05 compared to HG or LG untreated control (ANOVA and Dunnett’s a posteriori test), ^###^ *p* < 0.001, ^##^ *p* < 0.01, ^#^ *p* < 0.05 compared to HG corresponding conditions (ANOVA and Dunnett’s a posteriori test). HG, high-glucose DMEM (4.5 g/L); LG, low-glucose DMEM (1 g/L).

Drug-induced senescence in normal and cancer cells is a side effect of chemotherapy that limits its effectiveness [65,66]. Combined treatment with quercetin and dasatinib (an anticancer drug and tyrosine kinase inhibitor) may result in the selective killing of senescent cells (senolysis) in vitro and in vivo [67]. Our recent discovery that the quercetin derivative with blocked all hydroxy groups (3,5,7-triacetyloxy-2-(2,2-diphenylbenzo[d][1,3]dioxol-5-yl)-4H-chromen-4-one) exhibits a potent senolytic activity in etoposide-induced senescent MDA-MB-231 cells [29] prompted us to study the senolytic potential of mitQ7 in a cellular model of drug-induced senescence (Figure 8A).

To activate a senescence program in six breast cancer cell lines, cells were stimulated with 35 nM doxorubicin for 24 h, and then the anticancer drug was removed and the cells were cultured for an additional 7 days with medium exchange every 2 days. Doxorubicin-induced senescent breast cancer cells were then stimulated with 1 µM mitQ7 for 24 h to assess mitQ7-mediated senolytic activity. A common pattern of mitQ7-related response was observed in cells that harbor the following gene mutations *CCL4L2* (C-C motif chemokine ligand 4 like 2, a member of a family of secreted proteins that function in inflammatory and immunoregulatory processes), *MT-ND5* (mitochondrially encoded NADH: ubiquinone oxidoreductase core subunit 5, involved in mitochondrial electron transport, NADH to ubiquinone and mitochondrial respiratory chain complex I assembly), *MUC5AC* (predicted to be an extracellular matrix structural constituent, which is involved in phosphatidylinositol-mediated signaling) (Table S4).

Except of BT-20 cells, mitQ7 treatment at HG conditions in doxorubicin-induced senescent breast cancer cells resulted in the induced apoptosis compared to untreated doxorubicin-induced senescent breast cancer cells (Figure 8A and Figure S37). However, this effect was mild (Figure 8A and Figure S37). Except for BT-20 cells and MDA-MB-468 cells, similar results were obtained for the system with limited glucose availability; see Figure 8A and Figure S37. Surprisingly, except for MCF-7 cells, doxorubicin-induced senescent breast cancer cells with a glucose deficit (LG conditions) were more prone to apoptosis (Figure 8A and Figure S37). LG conditions in doxorubicin-induced senescent MDA-MB-468 cells resulted in massive apoptosis induction, as judged by Annexin V staining, which is clearly presented in Figure 8A and Figure S37. The same figures demonstrate that the limited availability of glucose enforces mitQ7-mediated senolytic activity in doxorubicin-induced senescent SK-BR-3 and MDA-MB-231 cells. Increased susceptibility to the apoptosis of drug-induced senescent breast cancer cells may be associated with gene mutations in the *HLA-DRB5* and *MT-ND4* genes in MDA-MB-231, BT-20, SK-BR-3 and MDA-MB-468 cells (Table S4). The loss of mitochondrial inner transmembrane potential (ΔΨm) is a severe mitochondrial dysfunction that affects ATP production and may be accompanied by the opening of mitochondrial permeability transition pores and mitochondria-mediated apoptosis [68]. However, perturbations in mitochondrial transmembrane potential (MMP) may also be associated with necrotic cell death and caspase-independent cell death [69]. Therefore, we assessed whether MMP could depend on mitQ7 (Figure 8B and Figure S38). Changes in the MMP of drug-induced senescent breast cancer cells were analyzed in both live cells with intact cell membranes (7-AAD-negative cells) and dying ones (7-AAD-positive cells); see Figure 8B and Figure S38. When glucose concentration was not a limiting factor (HG), mitQ7 promoted MMP depolarization primarily in live doxorubicin-induced senescent breast cancer cells (Figure 8B and Figure S38). In some experimental settings, limited glucose availability also caused a decrease in MMP in live cells; however, for MDA-MB-468 cells, a massive increase of about 50% of depolarized dead cells was also observed in untreated LG conditions compared to untreated HG conditions (Figure 8B and Figure S38). This observation was correlated with late apoptosis in almost the total population of MDA-MB-468 cells (Figure 8A and Figure S37). MitQ7 also induced the depolarization of MMP under LG conditions compared to the untreated LG control (Figure 8B and Figure S38). However, mitQ7-mediated effects were less pronounced due to a limited decrease associated with glucose availability in MMP (Figure 8B and Figure S38). The senolytic activity of mitQ7 under low-glucose conditions in BT-20, MDA-MB-231 and SK-BR-3 may be associated with gene mutations in the *KLF14* and *NBPF11* genes in these cells (Table S3). On the contrary, under high-glucose conditions, mitQ7-mediated senolytic activity was perhaps independent of genetic background (Figure 8). To the best of our knowledge, the effects of mitochondriotropic quercetin derivatives on MMP in senescent cancer cells were not analyzed. On the contrary, there are some data on mitochondriotropic quercetin derivative-mediated changes in MMP in normal and cancer cells as well as in isolated mitochondria [17,19]. The effects of two mitochondriotropic quercetin derivatives, 3-(4-O-triphenylphosphoniumbutyl) quercetin iodide (Q3BTPI) and its tetracetylated analogue (QTA3BTPI), were documented in isolated rat liver mitochondria [17]. These compounds promoted mitochondrial permeability transition (MPT), and Q3BTPI stimulated MMP depolarization [17]. Furthermore, 7-O-(4-triphenylphosphoniumbutyl)quercetin iodide (Q-7BTPI) induced oxidative stress-mediated necrosis in cultured cells that was not accompanied by significant perturbations of MMP [19]. Q-7BTPI was active against mouse colon cancer C-26 cells and fast-growing (doubling time 16 h) SV-40 immortalized mouse embryo fibroblast (MEF) cells, but it was not active against slow-growing (doubling time 3 days) MEF cells [19].

**Figure 9:**
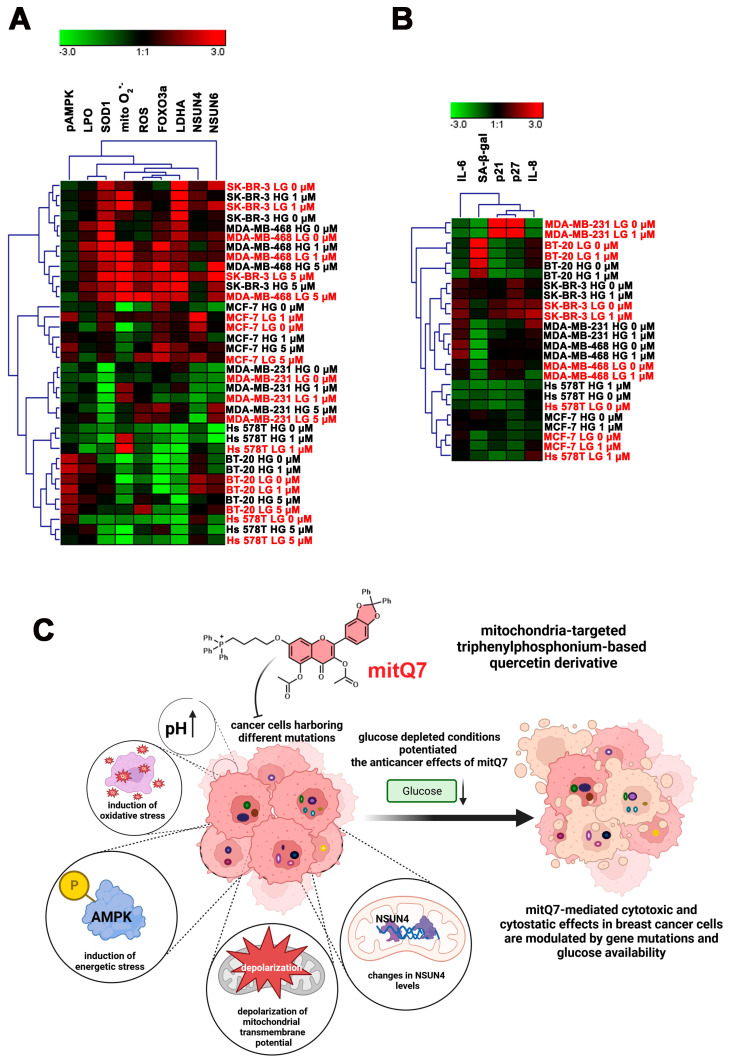
Clustering analysis of selected stress parameters ((**A**), pAMPK, LPO, SOD1, mitochondrial superoxide, ROS, FOXO3a, LDHA, NSUN4, and NSUN6) and senescence biomarkers ((**B**), SA-beta-gal, p21, p27, IL-6, and IL-8) as a function of glucose concertation and treatment with mitQ7 (1 or 5 µM) in six breast cancer cell lines. (**A**,**B**) Heat maps were generated from imaging flow cytometry-based data. Hierarchical clustering was performed using Genesis software, version 1.8.1. HG (in black), high-glucose DMEM (4.5 g/L); LG (in red), low-glucose DMEM (1 g/L). (**C**) A summarizing scheme. Mitochondria-targeted derivative of quercetin (mitQ7) promoted apoptotic cell death, oxidative stress conditions, energetic stress associated with the alkalization of intracellular pH and changes in the levels of mitochondrial protein NSUN4 in breast cancer cells. The anticancer effects of mitQ7 against breast cancer cells may be modulated by gene mutation status and glucose availability.

Since six breast cancer cell lines with different mutations and receptor status were used in the present study, we have also performed a data integration and clustering analysis to reveal some common responses after mitQ7 stimulation before and after activation of the drug-induced senescence program (Figure 9). The analysis of selected stress markers showed that mitQ7-treated SK-BR-3 and MDA-MB-468 cells (1 and 5 µM) responded similarly and were grouped together, and glucose concentration did not significantly affect the response (Figure 9A). This may rely on similar gene mutation pattern in these cells, namely gene mutations in *CFAP57*, *SPTA1*, *IGFN1*, *CLASP1*, *VIL1*, *RBM44*, *PASK*, *FARP2*, *MUC22*, *TULP4*, *PKD1L1*, *KCP*, *MGAM*, *CNTRL*, *STAT2*, *USP30*, *ABCC11*, *KCNH4*, *FASN*, *CACTIN*, *TMEM161A*, *LILRA1*, *MZF1* and *DGKK* (Table S3). Several gene mutations seem particularly interesting for mitochondria-related function, membrane transport and signaling pathways, namely gene mutations in *USP30* (deubiquitinating enzyme tethered to the mitochondrial outer membrane that acts as a key inhibitor of mitophagy), *ABCC11* (ATP-dependent transporter of the ATP-binding cassette (ABC) family that actively extrudes physiological compounds and xenobiotics from cells), *PASK* (serine/threonine-protein kinase involved in energy homeostasis and protein translation) and *DGKK* (diacylglycerol kinase that converts diacylglycerol/DAG into phosphatidic acid/phosphatidate/PA and regulates the respective levels of these two bioactive lipids). The MCF-7 cell line was grouped as a separate category, and 5 µM mitQ7 strongly affected the parameters of cellular stress, while the effect of 1 µM mitQ7 was more affected by glucose concentration (Figure 9A). In MDA-MB-231 and BT-20 cells, the effects of mitQ7 were also evident and modulated by glucose levels (Figure 9A). In Hs 578T cells, changes in cellular stress parameters were more pronounced at low-glucose concentration (Figure 9A). Cluster analysis of senescence markers revealed that in MDA-MB-231, MDA-MB-468, BT-20, and SK-BR-3 cells, the levels of senescence-related biomarkers were affected mainly by glucose concentration (Figure 9B). The most pronounced changes were observed in MDA-MB-231 cells cultured under high versus low-glucose conditions (Figure 9B). Glucose levels also modulated the response to mitQ7 in MCF-7 cells in terms of the expression of senescence markers (Figure 9B). MitQ7-mediated changes were the most evident in Hs 578T cells with limited effect of glucose concentration (Figure 9B).

## 4. Conclusions

We synthetized and characterized the physicochemical properties of three non-polar, mitochondria-targeted derivatives of quercetin. Since all hydroxy groups are blocked, the compounds are not able to break the peroxidation of lipids; thus, high lipophilicity and strong interactions with lipid bilayers are principal factors affecting the bioactivity of the three derivatives. We documented for the first time that mito-quercetin (mitQ7), in contrast to unmodified quercetin, when used at low micromolar concentration (1 µM), may induce apoptosis in a number of breast cancer cells. Furthermore, we show for the first time the importance of the genetic background, here the mutation status of breast cancer cells for the activity of quercetin derivatives, and we show that mito-quercetin is more effective in the elimination of breast cancer cells with different mutation status than quercetin (Figure 9C). We also found genes related to mitochondrial functions, which can modulate the response of breast cancer cells to quercetin derivatives. In addition, we show the significance of glucose levels for the anticancer activity of quercetin derivatives. We found that in MCF-7 cells, the cytotoxicity can be potentiated in glucose-depleted conditions, which is accompanied by mitochondrial oxidative stress, AMPK activation-mediated energetic stress associated with the alkalization of intracellular pH and elevated levels of NSUN4 (Figure 9C). Glucose-depleted conditions may also promote cellular senescence in mito-quercetin-treated BT-20 and Hs 578T cells. Mito-quercetin also eliminated doxorubicin-induced senescent breast cancer cells that was mediated by the depolarization of MMP. Doxorubicin-induced senescent MDA-MB-468 cells were also extremely sensitive to limited glucose availability compared to other doxorubicin-induced senescent breast cancer cells. Our results suggest that mitochondria-targeted quercetin may be used for effective elimination of cancer cells with different mutation status that can be also augmented by the modulation of glucose availability (Figure 9C).

## Data Availability

The data presented in this study are available in the supplementary material.

## Supplementary Materials

The following supporting information can be downloaded at: https://www.mdpi.com/article/10.3390/cancers15235614/s1. Supplementary Material 1: S1. Synthesis of mitochondrial derivatives: S1.1. General information, S1.2. Synthesis and identification of mitQ7, S1.3. Synthesis and identification of mitQ3, S1.4. Synthesis and identification of mitQ5. S2. NMR spectral data. S3. Kinetic measurements for quercetin. SCHEMES: Scheme S1. Synthetic pathway for mitQ7, Scheme S2. Synthetic pathway for mitQ3, Scheme S3. Synthetic pathway for mitQ5. TABLES: Table S1. Kinetic parameters for autoxidation of methyl linoleate DMPC liposomes in the presence of 1 µM quercetin at pH range from 4 to 9. Table S2. Thermodynamic parameters determined from DSC experiments. FIGURES: Figures S1–S25. The ^1^H and ^13^C NMR spectral data for quercetin derivatives. Figure S26. Plots of oxygen uptake during peroxidation inhibited by quercetin. Figure S27. ADME parameters calculated for quercetin. Figure S28. ADME parameters calculated for mitQ3. Figure S29. ADME parameters calculated for mitQ5. Figure S30. ADME parameters calculated for mitQ7. Figure S31. The effects of quercetin (Q) and mito-quercetin derivatives (mitQ3, 5, and 7) on metabolic activity of six breast cancer cell lines. Figure S32. Mitochondria-related gene mutation status and correlation analysis between the metabolic activity and the number of gene mutations in genes involved in mitochondrial functions in breast cancer cells. Figure S33. Kinase gene mutation status and correlation analysis between the metabolic activity and the number of kinase gene mutations in breast cancer cells. Figure S34. MitQ7-mediated changes in the phases of cell cycle in breast cancer cells. Figure S35. MitQ7-induced apoptosis in breast cancer cells. Figure S36. MitQ7-mediated changes in intracellular pH in breast cancer cells. Figure S37. MitQ7-mediated senolytic activity in doxorubicin-induced senescent breast cancer cells. Figure S38. MitQ7-mediated changes in mitochondrial transmembrane potential in doxorubicin-induced senescent breast cancer cells. Supplementary Material 2: Kobas_raw_data. Supplementary Material 3: Table S3. Intersection of gene mutations set in six breast cancer cell lines used in this study and presented in Figure 3 with the use of Kobas standalone software. Table S4. Intersection of gene mutations set linked with mitochondrial functions in six breast cancer cell lines used in this study and presented in Figure S32 with the use of Kobas standalone software. Table S5. Intersection of gene mutations set linked with kinases pathways in six breast cancer cell lines used in this study and presented in Figure S33 with the use of Kobas standalone software.
