# Supplementary material for: Mutation Status and Glucose Availability Affect the Response to Mitochondria-Targeted Quercetin Derivative in Breast Cancer Cells
Source: Cancers (Basel). 2023 Nov 28;15(23):5614. doi: 10.3390/cancers15235614 (PMC10705313; doi:10.3390/cancers15235614)
Supplement: Supplementary file 1 [file cancers-15-05614-s001.zip › mitQ_Supplementary Material 1.pdf]

SUPPLEMENTARY MATERIAL 1

Mutation status and glucose availability affect the response to mitochondria-targeted quercetin derivative in breast cancer cells

Paweł Przybylski[1#], Anna Lewińska[2#], Iwona Rzeszutek[2], Dominika Błoniarz[2], Aleksandra Moskal[2], Gabriela Betlej[2], Anna Deręgowska[2], Martyna Cybularczyk-Cecotka[1], Tomasz Szmatoła[3], Grzegorz Litwinienko[1*], Maciej Wnuk[2*]

[1]*Faculty of Chemistry, University of Warsaw, Pasteura 1, 02-093 Warsaw, Poland*

[2]*Institute of Biotechnology, College of Natural Sciences, University of Rzeszow, Pigonia 1, 35-310 Rzeszow, Poland*

[3]*Center of Experimental and Innovative Medicine, University of Agriculture in Krakow, al. Mickiewicza 24/28, 30-059 Cracow, Poland*

[#]These authors have contributed equally as first authors

*Correspondence: Grzegorz Litwinienko (litwin@chem.uw.edu.pl), Maciej Wnuk (mwnuk@ur.edu.pl).

TABLE OF CONTENTS

**S1.Synthesis of mitochondrial derivatives**	**S-3**
S1.1. General Information	S-3
S1.2. Synthesis and identification of mitQ7	S-3
S1.3. Synthesis and identification of mitQ3	S-7
S1.4. Synthesis and identification of mitQ5	S-10
**S2. NMR spectral data**	**S-14**
**S3. Kinetic measurements for quercetin**	**S-27**
**SCHEMES**	
**Scheme S1.** Synthetic pathway for mitQ7.	S-3
**Scheme S2.** Synthetic pathway for mitQ3.	S-7
**Scheme S3.** Synthetic pathway for mitQ5.	S-10
**TABLES**	
**Table S1.** Kinetic parameters for autoxidation of methyl linoleate DMPC liposomes in the presence of 1µM quercetin at pH range from 4 to 9.	S-27
**Table S2.** Thermodynamic parameters determined from DSC experiments.	S-27
**FIGURES**	
**Figure S1-25**. ^{1}H and ^{13}C NMR spectral data for quercetin derivatives.	S-14
**Figure S26.** Plots of oxygen uptake during peroxidation inhibited by quercetin.	S-27
**Figure S27**. ADME parameters calculated for quercetin.	S-28
**Figure S28**. ADME parameters calculated for mitQ3.	S-29
**Figure S29**. ADME parameters calculated for mitQ5.	S-30
**Figure S30**. ADME parameters calculated for mitQ7.	S-31
**Figure S31**. The effects of quercetin (Q) and mito-quercetin derivatives (mitQ3, 5, and 7) on metabolic activity of six breast cancer cell lines.	S-32
**Figure S32.** Mitochondria-related gene mutation status and correlation analysis between the metabolic activity and the number of gene mutations in genes involved in mitochondrial functions in breast cancer cells.	S-34
**Figure S33.** Kinase gene mutation status and correlation analysis between the metabolic activity and the number of kinase gene mutations in breast cancer cells.	S-37
**Figure S34**. MitQ7-mediated changes in the phases of cell cycle in breast cancer cells.	S-40
**Figure S35**. MitQ7-induced apoptosis in breast cancer cells.	S-41
**Figure S36**. MitQ7-mediated changes in intracellular pH in breast cancer cells.	S-43
**Figure S37**. MitQ7-mediated senolytic activity in doxorubicin-induced senescent breast cancer cells.	S-44
**Figure S38**. MitQ7-mediated changes in mitochondrial transmembrane potential in doxorubicin-induced senescent breast cancer cells.	S-46

1. Synthesis of mitochondrial derivatives

1.1. General Information

Synthetic pathways are based on protocols described by Biasutto et al.[1] All reagents and solvents were purchased from commercial suppliers and used without further purification. Thin layer chromatography (TLC) was performed using Merck Silica Gel F254, 0.20 mm thickness and the visualization was accomplished by irradiation at 254 nm. All aqueous solutions were prepared using distilled water. Saturated brine refers to an aqueous saturated sodium chloride solution. All products were purified by column chromatography using silica gel 60 M (40-63 µm, 230-440 mesh). NMR spectra were recorded at room temperature using Bruker 300 MHz spectrometer. Chemical shifts are reported relatively in δ-scale as parts per million (ppm) referenced to the residual solvent peak. Coupling constants *J* are given in Hertz (Hz) and the following abbreviations were used for indicating signal multiplicity: ^{1}H NMR: s = singlet, d = doublet, t = triplet, q = quartet, hept = heptet, m = multiplet and the respective combinations. High resolution mass spectra were measured with Agilent 6540 UHD Q-TOF, spectrum recorded at the highest resolution of 4 GH.

1.2. Synthesis and identification of mitQ7

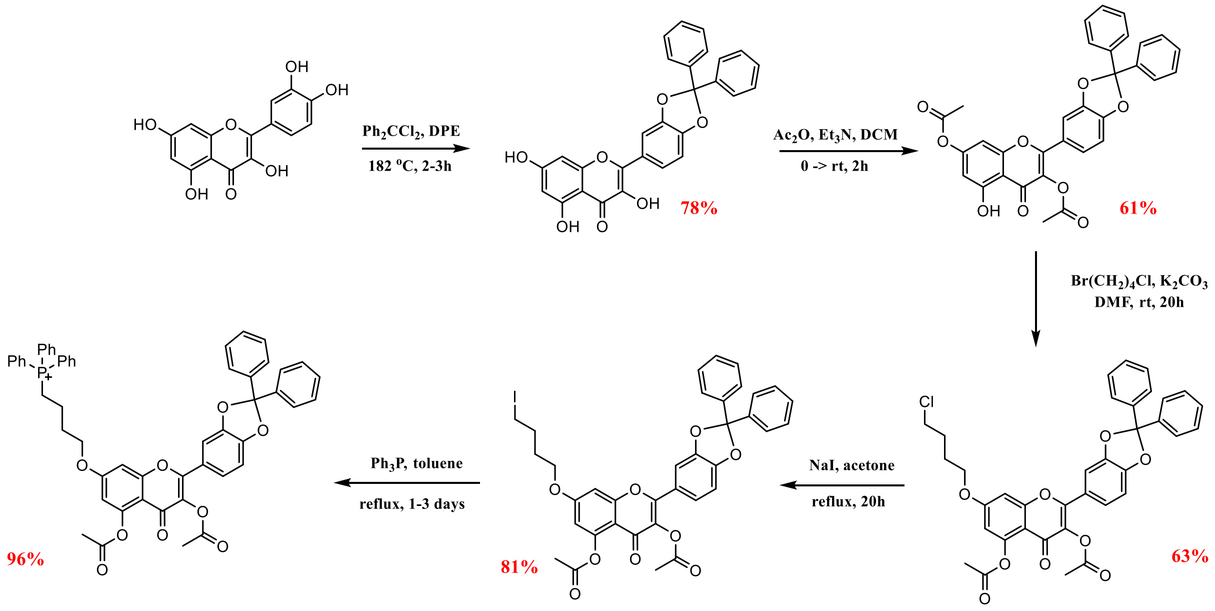

Scheme S1. Synthetic pathway for mitQ7.

[1] Biasutto, L., Mattarei, A., Paradisi, C. (2021). Synthesis and Testing of Novel Isomeric Mitochondriotropic Derivatives of Resveratrol and Quercetin . In: Weissig, V., Edeas, M. (eds) Mitochondrial Medicine . Methods in Molecular Biology, vol 2275. Humana, New York, NY. https://doi.org/10.1007/978-1-0716-1262-0_9.

3′,4′-O-diphenylmethane quercetin

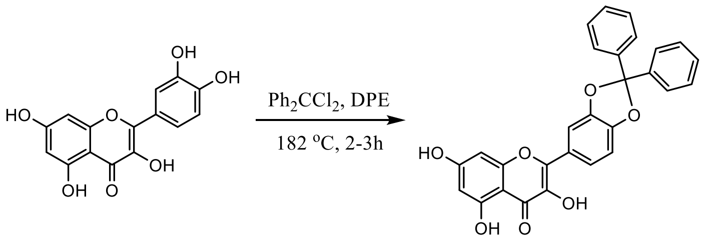

Quercetin (1.00 g, 3.31 mmol) and 1,1-dichlorodiphenylmethane (1.18 g, 4.96 mmol) were dissolved in diphenyl ether (40 ml) and the reaction mixture was heated to 182 °C with stirring. After 2 h, the mixture was cooled to room temperature, petroleum ether (50 ml) was added. The dark yellow crude product was obtained by filtration and purified by silica gel column chromatography (20-25% ethyl acetate in hexane as eluent) to give a product as a light yellow solid with isolated yield: 78.0% (1.20 g, 2.58 mmol). ^{1}H NMR (300 MHz, $CDCl_3$) δ 11.77 (s, 1H), 7.83 – 7.75 (m, 2H), 7.65 – 7.55 (m, 4H), 7.47 – 7.36 (m, 6H), 7.02 (d, *J* = 8.6 Hz, 1H), 6.59 (s, 1H), 6.43 (d, *J* = 2.1 Hz, 1H), 6.30 (d, *J* = 2.1 Hz, 1H). The obtained spectral data are consistent with the literature reports.[2]

3′,4′-O-diphenylmethane-3,7-diacetyl quercetin

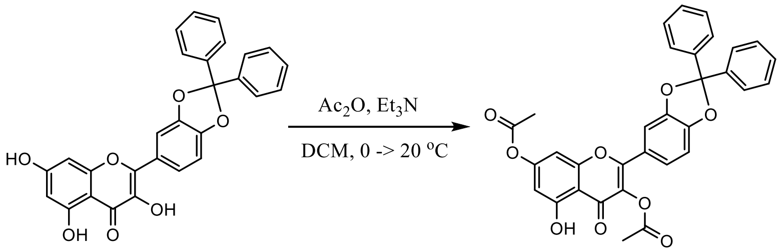

3′,4′-*O*-diphenylmethane quercetin (1.20 g, 2.58 mmol) was dissolved in 15 ml DCM and Et_3N (1.79 ml, 12.9 mmol) was added. The solution was cooled to 0°C and acetic anhydride (0.49 ml, 5.16 mmol) was added dropwise. The reaction mixture was allowed to warm up to room temperature and it was stirred until starting material disappeared completely (monitored by TLC, with petroleum ether/ethyl acetate 7:1 as eluent). After finishing, the reaction mixture was diluted by DCM and extracted 3 times with 1N HCl. Organic phase was dried over anhydrous $MgSO_4$. After solvent evaporation, the crude product was recrystallized from ethyl acetate/DCM/petroleum ether mixture to give pure 3′,4′-*O*-diphenylmethane-3,7-diacetyl quercetin as pale yellow solid with isolated yield 61% (0.87 g, 1.57 mmol). ^{1}H NMR (300 MHz, $CDCl_3$) δ 12.21 (s, 1H), 7.65 – 7.54 (m, 4H), 7.49 – 7.37 (m, 8H), 7.00 (d, *J* = 8.3 Hz, 1H), 6.82 (d, *J* = 2.0 Hz, 1H), 6.58 (d, *J* = 2.0 Hz, 1H), 2.36 (s, 3H), 2.33 (s, 3H). The obtained spectral data are consistent with the literature reports.[3]

[2] Cao, Z., Chen, J., Zhu, D., Yang, Z., Teng, W., Liu, G., Liu, B., Tao, C. *J. Chem. Res.* **2018**, Vol. 42, Issue 4, 189-193. https://doi.org/10.3184/174751918X15232706115112

[3] **CN104557891B** *Quercetin derivative and preparation method and application thereof.* https://worldwide.espacenet.com/patent/search/family/053075061/publication/CN104557891B?q=pn%3DCN104557891B Current Patent Assignee: NANJING HUITELAI PHARMACEUTICAL - CN104557891, 2017, B.

7-(4-O-chlorobutyl)-3′,4′-O-diphenylmethane-3,5-diacetyl quercetin

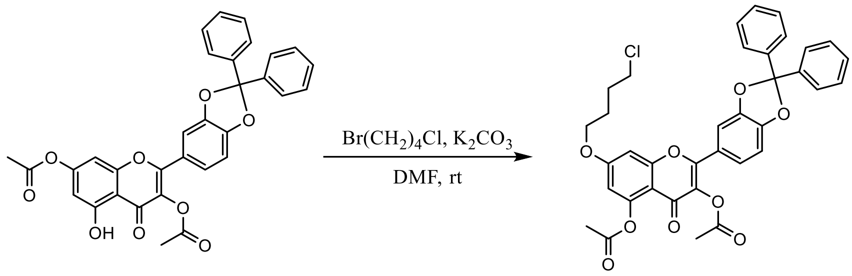

3′,4′-*O*-diphenylmethane-3,7-diacetyl quercetin (0.87 g, 1.57 mmol) and K_2CO_3 (0.24 g, 1.73 mmol) were dissolved in DMF (5 ml) and 1-bromo-4-chlorobutane (0.40 g, 2.36 mmol) was added. The reaction mixture was stirred overnight in room temperature. After confirming by TLC that all substrate had reacted, the reaction mixture was diluted with ethyl acetate (50 ml), transfer into the separating funnel and washed 3 times with 25 ml of 1N HCl. Organic layer was dried with anhydrous $MgSO_4$. The crude product was purified by column chromatography with elucidation by hexane:DCM:ethyl acetate (38:10:2) mixture to give 7-(4-*O*-chlorobutyl)-3′,4′-*O*-diphenylmethane-3,5-diacetyl quercetin as off-white solid with isolated yield 63% (0.64 g, 0.99 mmol). ^1H NMR (300 MHz, $CDCl_3$) δ 7.58 (ddd, *J* = 8.9, 4.0, 2.3 Hz, 4H), 7.47 – 7.34 (m, 8H), 6.98 (d, *J* = 8.2 Hz, 1H), 6.80 (d, *J* = 2.4 Hz, 1H), 6.61 (d, *J* = 2.4 Hz, 1H), 4.08 (t, *J* = 5.5 Hz, 2H), 3.63 (t, *J* = 6.0 Hz, 2H), 2.42 (s, 3H), 2.32 (s, 3H), 1.99 (dd, *J* = 4.0, 1.8 Hz, 4H). ^{13}C NMR (75 MHz, $CDCl_3$) δ 170.2, 169.8, 168.2, 163.0, 158.2, 154.7, 150.8, 149.7, 147.8, 139.8, 133.3, 129.6, 128.5, 126.4, 123.5, 123.5, 118.3, 111.1, 109.0, 108.8, 108.4, 99.4, 68.1, 44.6, 29.2, 26.4, 21.3, 20.9.

7-(4-O-iodobutyl)-3′,4′-O-diphenylmethane-3,5-diacetyl quercetin

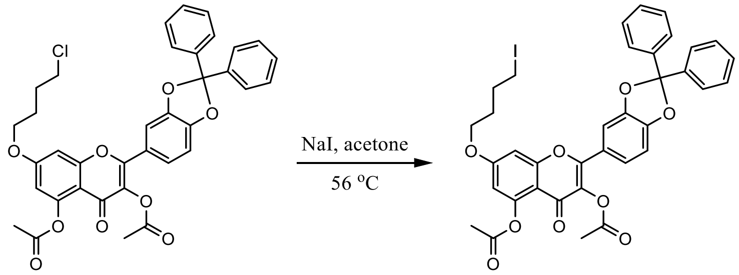

7-(4-*O*-chlorobutyl)-3′,4′-*O*-diphenylmethane-3,5-diacetyl quercetin (0.64 g, 0.99 mmol) and NaI (4.45 g, 29.7 mmol) were dissolved in acetone (11.5 ml) and heat at reflux overnight. After cooling, the reaction mixture was diluted with 100 ml of ethyl acetate, filtered through paper filter directly into the separating funnel and washed 3 times with 50 ml of water. Organic layer was dried with anhydrous $MgSO_4$. The crude product was purified by column chromatography with elucidation by hexane:ethyl acetate (8:2) mixture to give 7-(4-*O*-iodobutyl)-3′,4′-*O*-diphenylmethane-3,5-diacetyl quercetin as white solid with isolated yield 81% (0.59 g, 0.80 mmol). ^1H NMR (300 MHz, $CDCl_3$) δ 7.64 – 7.54 (m, 4H), 7.47 – 7.34 (m, 8H), 6.98 (d, J = 8.2 Hz, 1H), 6.79 (d, J = 2.4 Hz, 1H), 6.60 (d, J = 2.4 Hz, 1H), 4.07 (s, 2H), 3.26 (t, J = 6.5 Hz, 2H), 2.42 (s, 3H), 2.32 (s, 3H), 2.12 – 1.89 (m, 4H). ^{13}C NMR (75 MHz, $CDCl_3$) δ 170.2, 169.7,

168.2, 163.0, 158.2, 154.7, 150.8, 149.7, 147.8, 139.8, 133.3, 129.6, 128.5, 126.4, 123.5, 123.5, 118.3, 111.1, 108.9, 108.8, 108.4, 99.4, 67.8, 30.0, 29.9, 21.3, 20.9, 6.0.

7-(4-O-butyl triphenylphosphonium)-3′,4′-O-diphenylmethane-3,5-diacetyl quercetin iodide

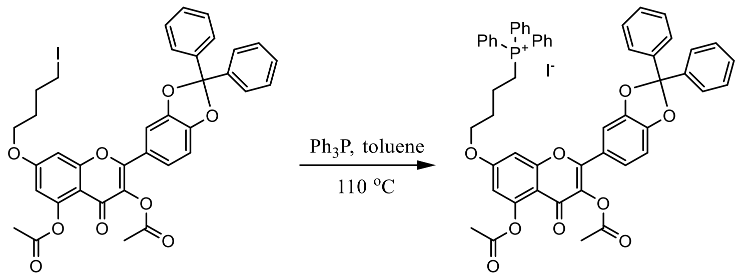

7-(4-*O*-iodobutyl)-3′,4′-*O*-diphenylmethane-3,5-diacetyl quercetin (0.59 g, 0.80 mmol) and Ph_3P (1.05 g, 4.0 mmol) were dissolved in toluene (15 ml) and heated at reflux for 2-3 days with monitoring the reaction course by TLC. When all the substrate had reacted, the reaction mixture was cooled and the solvent was evaporated under reduced pressure. The crude product was purified by column chromatography with elucidation by DCM:methanol (96:4) mixture to give 7-(4-*O*-butyl triphenylphosphonium)-3′,4′-*O*-diphenylmethane-3,5-diacetyl quercetin iodide as yellow solid with isolated yield 96% (0.77 g, 0.77 mmol). ^{1}H NMR (300 MHz, $CDCl_3$) δ 7.89 – 7.70 (m, 10H), 7.66 (dd, *J* = 7.2, 2.9 Hz, 6H), 7.56 (dd, *J* = 6.3, 2.6 Hz, 4H), 7.46 – 7.31 (m, 8H), 6.96 (d, *J* = 8.3 Hz, 1H), 6.83 (d, *J* = 2.4 Hz, 1H), 6.45 (d, *J* = 2.4 Hz, 1H), 4.16 (t, *J* = 5.3 Hz, 2H), 3.79 (m, 2H), 2.38 (s, 3H), 2.33 – 2.21 (m, 5H), 1.92 – 1.76 (m, 2H). ^{13}C NMR (75 MHz, $CDCl_3$) δ 170.0, 169.6, 168.0, 162.7, 158.0, 154.5, 150.4, 149.6, 147.6, 139.6, 135.2, 135.2, 133.7, 133.6, 133.0, 130.6, 130.5, 129.4, 128.4, 126.2, 123.5, 123.2, 118.4, 118.1, 117.3, 110.8, 108.9, 108.8, 108.2, 99.4, 67.7, 29.6, 29.1, 28.9, 22.7, 22.0, 21.1, 20.7, 19.2. HRMS (ESI) calcd for [M-I^-] $C_{54}H_{44}O_9P$ 867.2723, found 867.2731.

1.3. Synthesis and identification of mitQ3

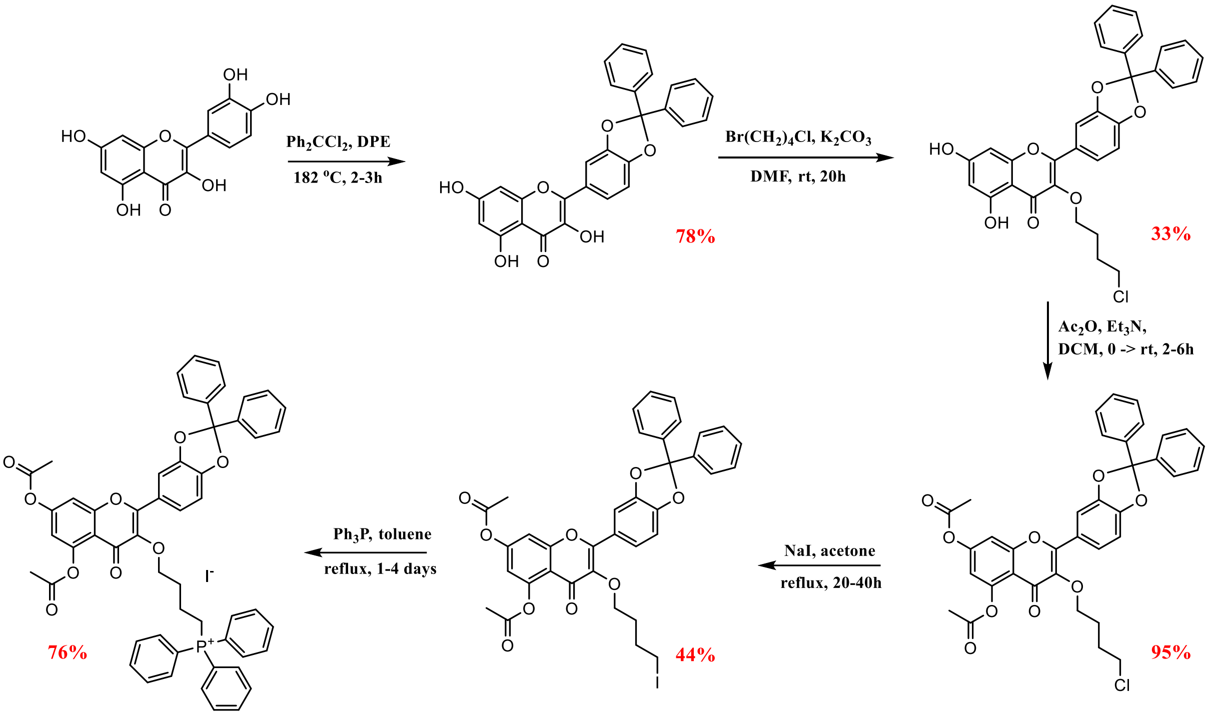

Scheme S2. Synthetic pathway for mitQ3.

3-(4-O-chlorobutyl)-3′,4′-O-diphenylmethane quercetin

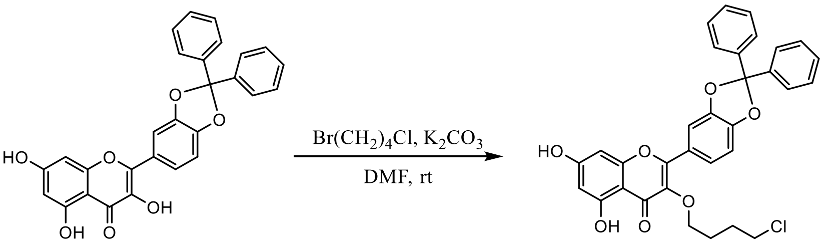

3′,4′-*O*-diphenylmethane-3,7-diacetyl quercetin (1.20 g, 2.58 mmol) and K_2CO_3 (0.39 g, 2.84 mmol) were dissolved in DMF (8 ml) and 1-bromo-4-chlorobutane (0.66 g, 3.87 mmol) was added. The reaction mixture was stirred overnight in room temperature. After confirming by TLC that all substrate had reacted, the reaction mixture was diluted with ethyl acetate (100 ml), transfer into the separating funnel and washed 3 times with 50 ml of 1N HCl. Organic layer was dried with anhydrous $MgSO_4$. The crude product was purified by column chromatography with elucidation by hexane:ethyl acetate (8:2) mixture to give 3-(4-*O*-chlorobutyl)-3′,4′-*O*-diphenylmethane quercetin as light yellow solid with isolated yield 33% (0.47 g, 0.85 mmol). ^{1}H NMR (300 MHz, $CDCl_3$) δ 12.60 (s, 1H), 7.82 – 7.51 (m, 6H), 7.51 – 7.30 (m, 7H), 6.99 (d, *J* = 8.3 Hz, 1H), 6.43 (d, *J* = 1.6 Hz, 1H), 6.33 (d, *J* = 1.8 Hz, 1H), 3.94 (t, *J* = 5.5 Hz, 2H), 3.46

(t, J = 6.0 Hz, 2H), 1.94 – 1.71 (m, 4H). The obtained spectral data are consistent with the literature reports.[4]

3-(4-O-chlorobutyl)-3',4'-O-diphenylmethane-5,7-diacetyl quercetin

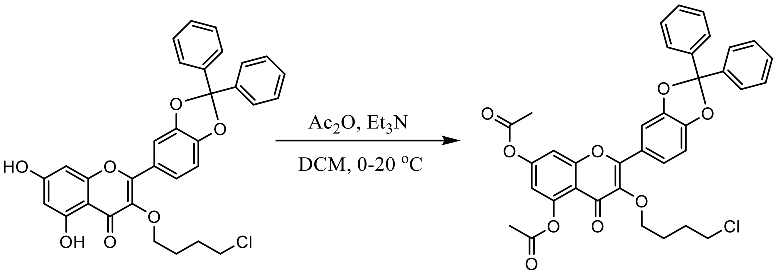

3-(4-*O*-chlorobutyl)-3′,4′-*O*-diphenylmethane quercetin (0.47 g, 0.85 mmol) was dissolved in 15 ml DCM and Et_3N (7.07 ml, 51 mmol) was added. The solution was cooled to 0°C and acetic anhydride (2.57 ml, 27.2 mmol) was added dropwise. The reaction mixture was allowed to warm up to room temperature and it was stirred until starting material disappeared completely (monitored by TLC, with hexane:ethyl acetate 8:2 as eluent; the fully reacted reaction mixture turned colorless). After finishing, the reaction mixture was diluted by DCM and extracted 3 times with 1N HCl. Organic phase was dried over anhydrous $MgSO_4$. After solvent evaporation, the crude product in the form of colorless oil turned out to be sufficiently pure to be used in next synthesis stage without further purification. Isolated yield: 95% (0.52 g, 0.81 mmol). ^{1}H NMR (300 MHz, $CDCl_3$) δ 7.67 – 7.56 (m, 6H), 7.47 – 7.35 (m, 6H), 7.27 (d, J = 2.2 Hz, 1H), 7.00 (dd, J = 8.1, 0.5 Hz, 1H), 6.82 (d, J = 2.2 Hz, 1H), 3.94 (t, J = 5.9 Hz, 2H), 3.48 (t, J = 6.3 Hz, 2H), 2.46 (s, 3H), 2.33 (s, 3H), 1.96 – 1.74 (m, 4H). ^{13}C NMR (75 MHz, $CDCl_3$) δ 173.2, 169.5, 168.1, 156.6, 155.1, 153.7, 150.3, 149.4, 147.5, 140.3, 139.8, 139.5, 129.6, 129.5, 128.5, 128.5, 126.3, 126.3, 124.1, 123.8, 118.1, 115.2, 113.2, 108.9, 108.6, 71.7, 64.8, 44.8, 29.1, 27.4, 21.3, 21.2.

3-(4-O-iodobutyl)-3',4'-O-diphenylmethane-5,7-diacetyl quercetin

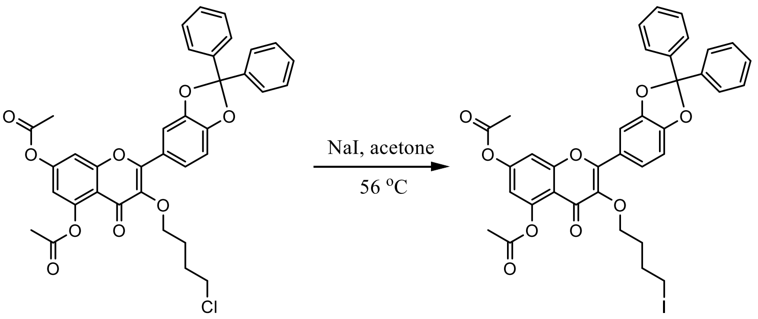

3-(4-*O*-chlorobutyl)-3′,4′-*O*-diphenylmethane-5,7-diacetyl quercetin (0.52 g, 0.81 mmol) and NaI (3.65 g, 24.3 mmol) were dissolved in acetone (9.5 ml) and heat at reflux overnight. Next day, small amount of solution was taken, evaporated under reduced pressure and measured by ^{1}H NMR to calculate the reaction conversion (Rf of substrate and product are the same). If there

[4] Mattarei, A., Biasutto, L., Marotta, E., De Marchi, U., Sassi, N., Garbisa, S., Zoratti, M., Paradisi, C. A mitochondriotropic derivative of quercetin: a strategy to increase the effectiveness of polyphenols. *ChemBioChem* **2008**, 9, 2633–2642 (10.1002/cbic.200800162)

was no substrate traces on ^{1}H NMR spectrum, the reaction mixture was cooled, diluted with 100 ml of ethyl acetate, filtered through paper filter directly into the separating funnel and washed 3 times with 50 ml of water. Organic layer was dried with anhydrous $MgSO_4$. The crude product was purified by column chromatography with elucidation by hexane:ethyl acetate (8:2) mixture to give 3-(4-*O*-iodobutyl)-3′,4′-*O*-diphenylmethane-5,7-diacetyl quercetin as white solid with isolated yield 44% (0.26 g, 0.36 mmol). ^{1}H NMR (300 MHz, $CDCl_3$) δ 7.69 – 7.55 (m, 6H), 7.47 – 7.36 (m, 6H), 7.27 (d, *J* = 2.2 Hz, 1H), 7.01 (d, *J* = 8.2 Hz, 1H), 6.81 (d, *J* = 2.2 Hz, 1H), 3.93 (t, *J* = 6.1 Hz, 2H), 3.11 (t, *J* = 6.7 Hz, 2H), 2.46 (s, 3H), 2.34 (s, 3H), 1.97 – 1.83 (m, 2H), 1.82 – 1.71 (m, 2H). ^{13}C NMR (75 MHz, $CDCl_3$) δ 173.2, 169.6, 168.2, 156.7, 155.1, 153.8, 150.3, 149.5, 147.6, 140.3, 139.9, 129.5, 128.5, 126.4, 124.1, 123.9, 118.1, 115.3, 113.3, 109.0, 108.9, 108.7, 71.4, 30.9, 30.0, 21.3, 21.3, 6.8.

3-(4-O-butyl triphenylphosphonium)-3′,4′-O-diphenylmethane-5,7-diacetyl quercetin iodide

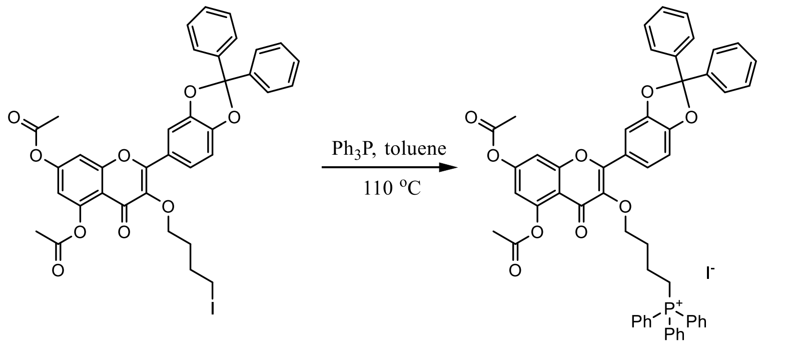

3-(4-*O*-iodobutyl)-3′,4′-*O*-diphenylmethane-5,7-diacetyl quercetin (0.26 g, 0.36 mmol) and Ph_3P (0.47 g, 1.80 mmol) were dissolved in toluene (10 ml) and heated at reflux for 1-3 days with monitoring the reaction course by TLC. When all the substrate had reacted, the reaction mixture was cooled and the solvent was evaporated under reduced pressure. The crude product was purified by column chromatography with elucidation by DCM:methanol (96:4) mixture to give 3-(4-*O*-butyl triphenylphosphonium)-3′,4′-*O*-diphenylmethane-5,7-diacetyl quercetin iodide as yellow solid with isolated yield 76% (0.27 g, 0.27 mmol). ^{1}H NMR (300 MHz, $CDCl_3$; mixture of isomers) δ 7.92 – 7.82 (m, 6H), 7.82 – 7.74 (m, 3H), 7.73 – 7.64 (m, 7H), 7.64 – 7.51 (m, 5H), 7.45 – 7.34 (m, 6H), 7.27 (d, *J* = 2.2 Hz, 0.5H), 6.95 (t, *J* = 8.2 Hz, 1H), 6.78 (d, *J* = 2.2 Hz, 0.5H), 6.76 (d, *J* = 2.0 Hz, 0.4H), 6.50 (d, *J* = 2.0 Hz, 0.4H), 4.11 – 3.88 (m, 4H), 2.32 (s, 1.7H), 2.31 (s, 1.3H), 2.26 – 2.07 (m, 2H), 2.07 – 1.95 (m, 2H), 1.90 (s, 3H). ^{13}C NMR (75 MHz, $CDCl_3$) δ 179.2, 173.2, 169.1, 168.4, 168.1, 161.6, 157.2, 156.5, 155.9, 155.6, 155.2, 153.9, 150.0, 149.9, 149.6, 147.7, 147.7, 139.7, 139.6, 139.6, 138.1, 135.1, 133.9, 133.7, 130.7, 130.6, 130.5, 130.4, 129.4, 128.6, 128.4, 126.2, 124.2, 123.8, 123.7, 123.6, 118.9, 118.8, 118.2, 118.1, 117.8, 117.7, 114.9, 113.3, 109.1, 109.0, 108.8, 108.2, 104.9, 101.0, 70.7, 69.3, 29.7, 29.5, 29.3, 29.1, 21.3, 20.7, 19.2, 18.7, 14.2. HRMS (ESI) calcd for [M-I$^-$] $C_{54}H_{44}O_9P$ 867.2723, found 867.2727.

1.4. Synthesis and identification of mitQ5

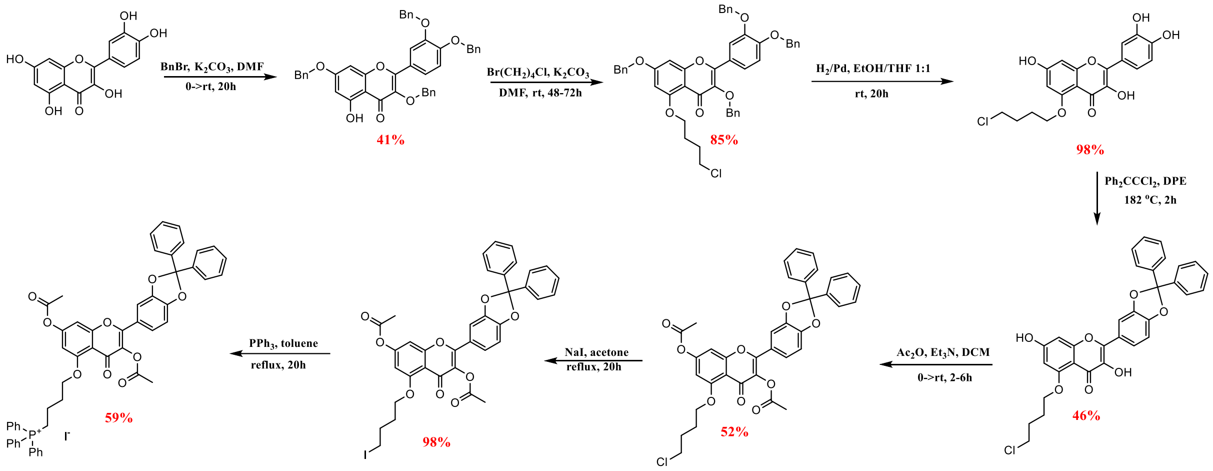

Scheme S3. Synthetic pathway for mitQ5.

3,3′,4′,7-tetrabenzyl quercetin

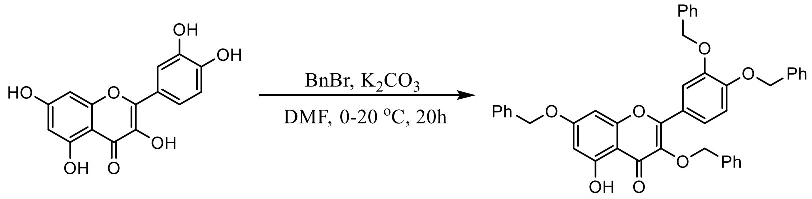

Quercetin (1.00 g, 3.31 mmol) and K_2CO_3 (1.60 g, 11.6 mmol) were dissolved in DMF (20 ml), cooled to 0 °C and BnBr (1.38 ml, 11.6 mmol) was added dropwise with vigorous stirring. After 2 hours, the reaction mixture was allowed to warm to room temperature and the stirring was continued for night. Then, after TLC confirmation, that all the substrate had reacted, the mixture was diluted with 100 ml of ethyl acetate and washed with 1N HCl (3x50 ml). Organic phase was dried with anhydrous $MgSO_4$ and the solvent was evaporated under reduced pressure. Purification by column chromatography using gradient mixture of hexane/DCM (1:1 to 1:2) as eluent led to obtain 3,3′,4′,7-tetrabenzyl quercetin as yellow solid with isolated yield: 41% (0.90 g, 1.36 mmol) The obtained spectral data are consistent with the literature reports:[5] ^{1}H NMR (300 MHz, $CDCl_3$) δ 7.71 (d, *J* = 2.1 Hz, 1H), 7.56 (dd, *J* = 8.6, 2.1 Hz, 1H), 7.50 – 7.19 (m, 21H), 6.97 (d, *J* = 8.7 Hz, 1H), 6.47 (d, *J* = 2.2 Hz, 1H), 6.44 (d, *J* = 2.2 Hz, 1H), 5.25 (s, 2H), 5.13 (s, 2H), 5.05 (s, 2H), 5.00 (s, 2H).

[5] **CN104557891B** *Quercetin derivative and preparation method and application thereof.* https://worldwide.espacenet.com/patent/search/family/053075061/publication/CN104557891B?q=pn%3DCN104557891B Current Patent Assignee: NANJING HUITELAI PHARMACEUTICAL - CN104557891, 2017, B.

3,3′,4′,7-tetrabenzyl-5-(4-O-chlorobutyl) quercetin

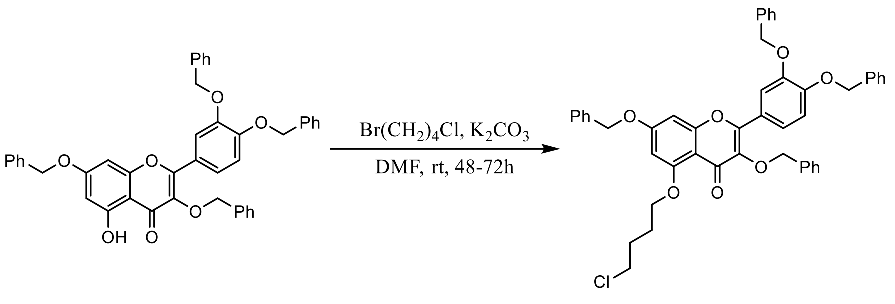

3,3′,4′,7-tetrabenzyl quercetin (0.90 g, 1.36 mmol) and K_2CO_3 (0.28 g, 2.04 mmol) were dissolved in DMF (5 ml) and 1-bromo-4-chlorobutane (0.47 g, 2.72 mmol) was added. The reaction mixture was stirred overnight in room temperature. After confirming by TLC that all substrate had reacted, the reaction mixture was diluted with ethyl acetate (100 ml), transfer into the separating funnel and washed 3 times with 50 ml of 1N HCl. Organic layer was dried with anhydrous $MgSO_4$. The solvent was evaporated and crude product was recrystallized from ethyl acetate/hexane mixture. Isolated yield: 85% (0.87 g, 1.15 mmol). ^{1}H NMR (300 MHz, $CDCl_3$) δ 7.72 (d, *J* = 2.1 Hz, 1H), 7.53 (dd, *J* = 8.6, 2.1 Hz, 1H), 7.50 – 7.28 (m, 17H), 7.25 – 7.16 (m, 3H), 6.95 (d, *J* = 8.7 Hz, 1H), 6.52 (d, *J* = 2.2 Hz, 1H), 6.41 (d, *J* = 2.2 Hz, 1H), 5.23 (s, 2H), 5.13 (s, 2H), 5.07 (s, 2H), 4.95 (s, 2H), 4.11 (t, *J* = 5.2 Hz, 2H), 3.70 (t, *J* = 6.0 Hz, 2H), 2.27 – 2.05 (m, 4H). ^{13}C NMR (75 MHz, $CDCl_3$) δ 174.0, 162.9, 160.4, 158.8, 153.3, 150.6, 148.3, 139.9, 137.2, 136.9, 135.9, 129.0, 128.9, 128.7, 128.6, 128.3, 128.1, 127.9, 127.7, 127.5, 127.3, 124.1, 122.2, 115.3, 113.9, 109.9, 97.4, 93.6, 74.3, 71.1, 71.0, 70.6, 68.6, 45.2, 29.3, 26.3.

5-(4-O-chlorobutyl) quercetin

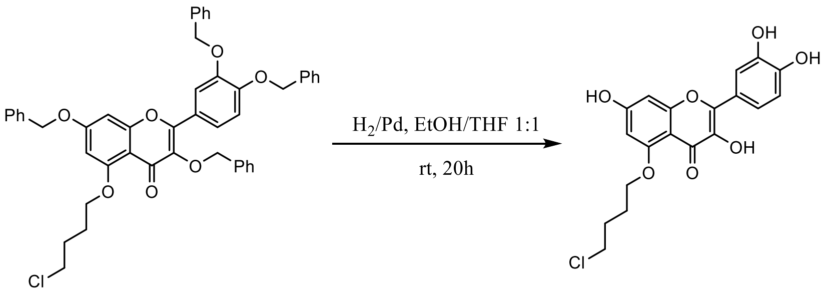

3,3′,4′,7-tetrabenzyl-5-(4-*O*-chlorobutyl) quercetin (0.87 g, 1.15 mmol) was dissolved in a mixture of ethanol : tetrahydrofuran 1:1 (60 ml). The mixture was deoxygenated with the use of nitrogen and catalytic amount of palladium on carbon 10 wt % was added. The reaction was stirred at room temperature under hydrogen atmosphere through night. Then, the reaction mixture was filtered on Celite® and washed with ethanol (100 ml). The filtrate was concentrated under reduced pressure to give crude product (confirmed by ^{1}H NMR), which was used in next step without further purification. Isolated yield: 98% (0.44 g, 1.13 mmol).

^{1}H NMR (300 MHz, DMSO-d_6) δ 9.47 (br s, 2H), 8.77 (br s, 1H), 7.64 (d, *J* = 2.2 Hz, 1H), 7.49 (dd, *J* = 8.5, 2.2 Hz, 1H), 6.87 (d, *J* = 8.5 Hz, 1H), 6.43 (t, *J* = 7.3 Hz, 1H), 6.34 (d, *J* = 2.0 Hz, 1H), 4.06 (t, *J* = 5.7 Hz, 2H), 3.79 (t, *J* = 6.6 Hz, 2H), 2.10 (ddd, *J* = 21.0, 14.9, 7.1 Hz, 2H), 2.00 – 1.83 (m, 2H).

5-(4-O-chlorobutyl)-3′,4′-O-diphenylmethane quercetin

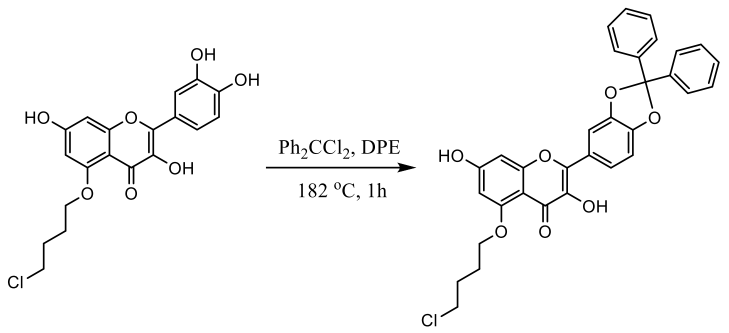

5-(4-*O*-chlorobutyl) quercetin (0.44 g, 1.13 mmol) and 1,1-dichlorodiphenylmethane (0.40 g, 1.70 mmol) were dissolved in diphenyl ether (15 ml) and the reaction mixture was heated to 182 °C with stirring. After 2 h, the mixture was cooled to room temperature, petroleum ether (100 ml) was added. The dark brown crude product was obtained by filtration and purified by silica gel column chromatography (20-50% ethyl acetate in petroleum ether as eluent) to give a product as a light brown solid with isolated yield: 46% (0.29 g, 0.52 mmol). ^{1}H NMR (300 MHz, DMSO-d_6) δ 10.75 (s, 1H), 9.06 (s, 1H), 7.83 – 7.74 (m, 2H), 7.58 (ddd, *J* = 7.8, 4.5, 2.8 Hz, 4H), 7.53 – 7.41 (m, 6H), 7.21 (d, *J* = 8.4 Hz, 1H), 6.52 (d, *J* = 2.1 Hz, 1H), 6.35 (d, *J* = 2.1 Hz, 1H), 4.06 (t, *J* = 5.7 Hz, 2H), 3.78 (t, *J* = 6.6 Hz, 2H), 2.15 – 1.98 (m, 2H), 1.98 – 1.84 (m, 2H).

5-(4-O-chlorobutyl)-3,7-diacetyl-3′,4′-O-diphenylmethane quercetin

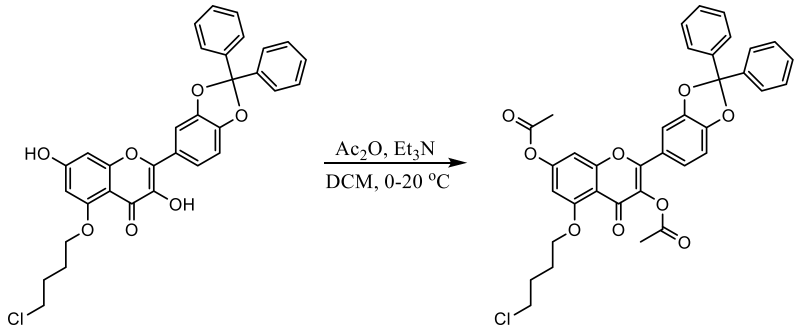

5-(4-*O*-chlorobutyl)-3′,4′-*O*-diphenylmethane quercetin (0.29 g, 0.52 mmol) was dissolved in 20 ml of DCM and Et_3N (1.08 ml, 7.80 mmol) was added. The solution was cooled to 0°C and acetic anhydride (0.29 ml, 3.12 mmol) was added dropwise. The reaction mixture was allowed to warm up to room temperature and it was stirred until starting material disappeared completely (monitored by TLC, with hexane:ethyl acetate 8:2 as eluent). After finishing, the reaction mixture was diluted by DCM and extracted 3 times with 1N HCl. Organic phase was dried over anhydrous $MgSO_4$. After solvent evaporation, crude product was purified by column chromatography with the use of mixture of hexane/ethyl acetate/DCM 19:4:1 as eluent. Pure 5-(4-*O*-chlorobutyl)-3,7-diacetyl-3′,4′-*O*-diphenylmethane quercetin is a white solid. Isolated yield: 52% (0.17 g, 0.27 mmol). ^{1}H NMR (300 MHz, $CDCl_3$) δ 7.65 – 7.54 (m, 4H), 7.47 – 7.35 (m, 8H), 7.02 – 6.95 (m, 1H), 6.90 (d, *J* = 2.1 Hz, 1H), 6.55 (d, *J* = 2.1 Hz, 1H), 4.10 (d, *J* = 4.5 Hz, 2H), 3.66 (t, *J* = 6.0 Hz, 2H), 2.34 (s, 3H), 2.33 (s, 3H), 2.20 – 1.99 (m, 4H). ^{13}C NMR (75 MHz, $CDCl_3$) δ 170.7, 168.5, 168.2, 160.4, 157.9, 154.9, 153.9, 149.7, 147.8, 139.7, 133.8,

129.5, 128.5, 126.4, 123.5, 123.4, 118.2, 112.4, 108.8, 108.4, 103.1, 101.9, 68.8, 45.1, 29.1, 26.3, 21.3, 20.9.

5-(4-O-iodobutyl)-3′,4′-O-diphenylmethane-3,7-diacetyl quercetin

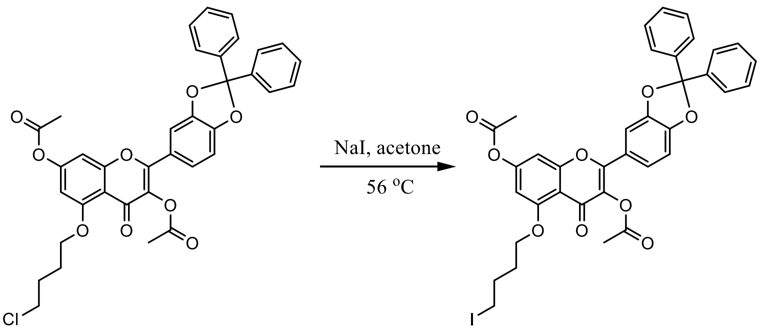

5-(4-*O*-chlorobutyl)-3,7-diacetyl-3′,4′-*O*-diphenylmethane quercetin (0.17 g, 0.27 mmol) and NaI (1.21 g, 8.1 mmol) were dissolved in acetone (5 ml) and heat at reflux overnight. After cooling, the reaction mixture was diluted with 50 ml of ethyl acetate, filtered through paper filter directly into the separating funnel and washed 3 times with 30 ml of water. Organic layer was dried with anhydrous $MgSO_4$. Solvent was evaporated under reduced pressure to get the crude product (confirmed by ^1H NMR) as a white solid, which was used in next step without further purification. Isolated yield: 98% (0.19 g, 0.26 mmol). ^1H NMR (300 MHz, $CDCl_3$) δ 7.63 – 7.53 (m, 4H), 7.46 – 7.35 (m, 9H), 7.01 – 6.94 (m, 1H), 6.90 (d, *J* = 2.1 Hz, 1H), 6.54 (d, *J* = 2.1 Hz, 1H), 4.08 (t, *J* = 5.8 Hz, 2H), 3.31 (t, *J* = 6.5 Hz, 2H), 2.34 (s, 3H), 2.34 (s, 3H), 2.15 – 1.99 (m, 4H).

5-(4-O-butyl triphenylphosphonium)-3′,4′-O-diphenylmethane-3,7-diacetyl quercetin iodide

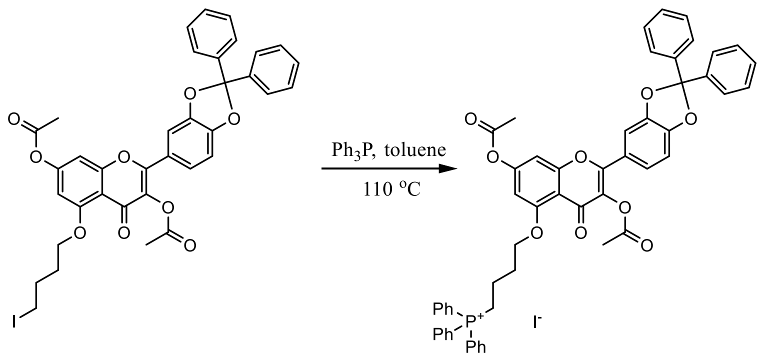

5-(4-*O*-iodobutyl)-3′,4′-*O*-diphenylmethane-3,7-diacetyl quercetin (0.19 g, 0.26 mmol) and Ph_3P (0.34 g, 1.30 mmol) were dissolved in toluene (8 ml) and heated at reflux for 1-3 days with monitoring the reaction course by TLC. When all the substrate had reacted, the reaction mixture was cooled and the solvent was evaporated under reduced pressure. The crude product was purified by column chromatography with elucidation by DCM:methanol (96:4) mixture to give 5-(4-*O*-butyl triphenylphosphonium)-3′,4′-*O*-diphenylmethane-3,7-diacetyl quercetin iodide as yellow solid with isolated yield: 59% (0.16 g, 0.16 mmol). ^1H NMR (300 MHz, $CDCl_3$) δ 7.88 – 7.67 (m, 9H), 7.63 (dd, *J* = 7.1, 2.9 Hz, 6H), 7.57 – 7.47 (m, 4H), 7.45 – 7.28 (m, 8H), 6.95 (d, *J* = 8.3 Hz, 1H), 6.87 (s, 1H), 6.58 (s, 1H), 4.15 (m, 2H), 3.89 (m, 2H), 2.31 (s, 3H), 2.22 (m, 2H), 2.12 (s, 3H), 2.01 (m, 2H). ^{13}C NMR (75 MHz, $CDCl_3$) δ 170.4, 168.4,

167.6, 159.7, 157.5, 154.9, 153.9, 149.6, 147.6, 139.4, 134.9, 134.8, 133.7, 133.5, 133.3, 130.5, 130.3, 129.4, 128.3, 126.1, 123.3, 123.0, 118.7, 118.1, 117.6, 111.7, 108.7, 108.0, 103.2, 102.1, 69.3, 30.9, 29.6, 28.6, 28.3, 22.4, 21.7, 21.2, 20.6, 20.1. HRMS (ESI) calcd for $[M-I^-]$ $C_{54}H_{44}O_9P$ 867.2723, found 867.2734.

2. NMR spectral data

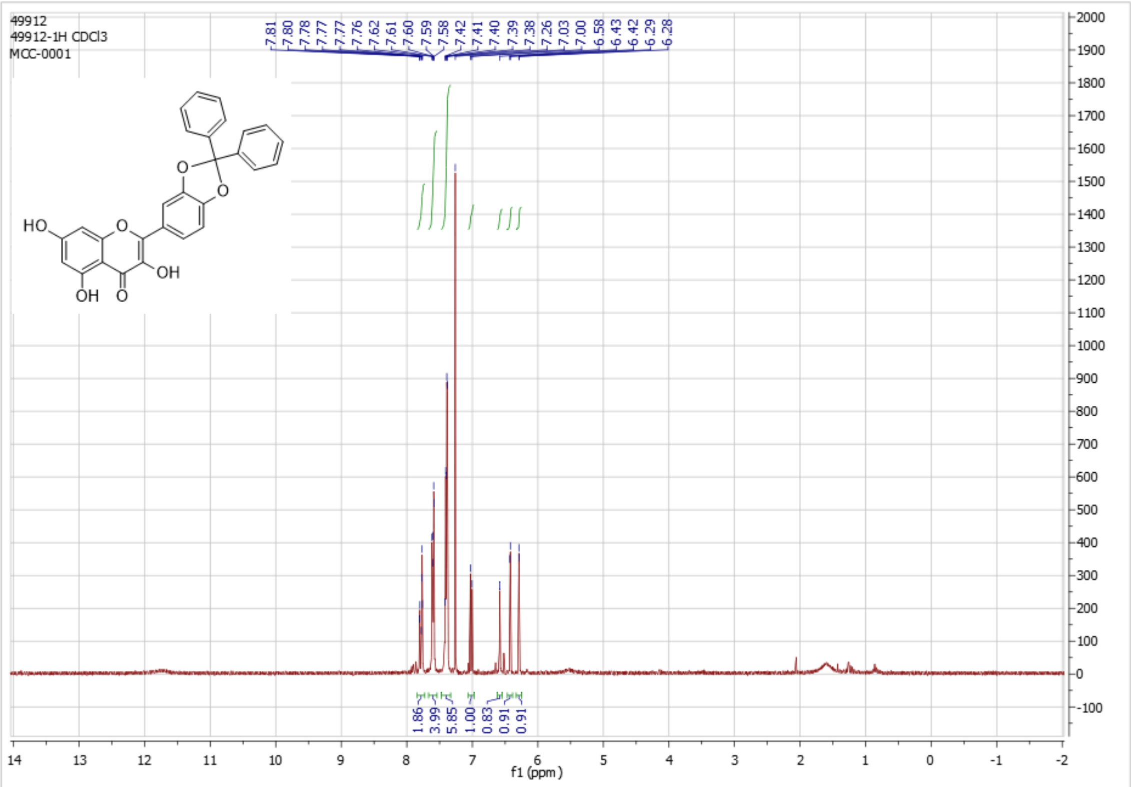

Figure S1. ^{1}H NMR (300 MHz, $CDCl_3$) of 3′,4′-*O*-diphenylmethane quercetin.

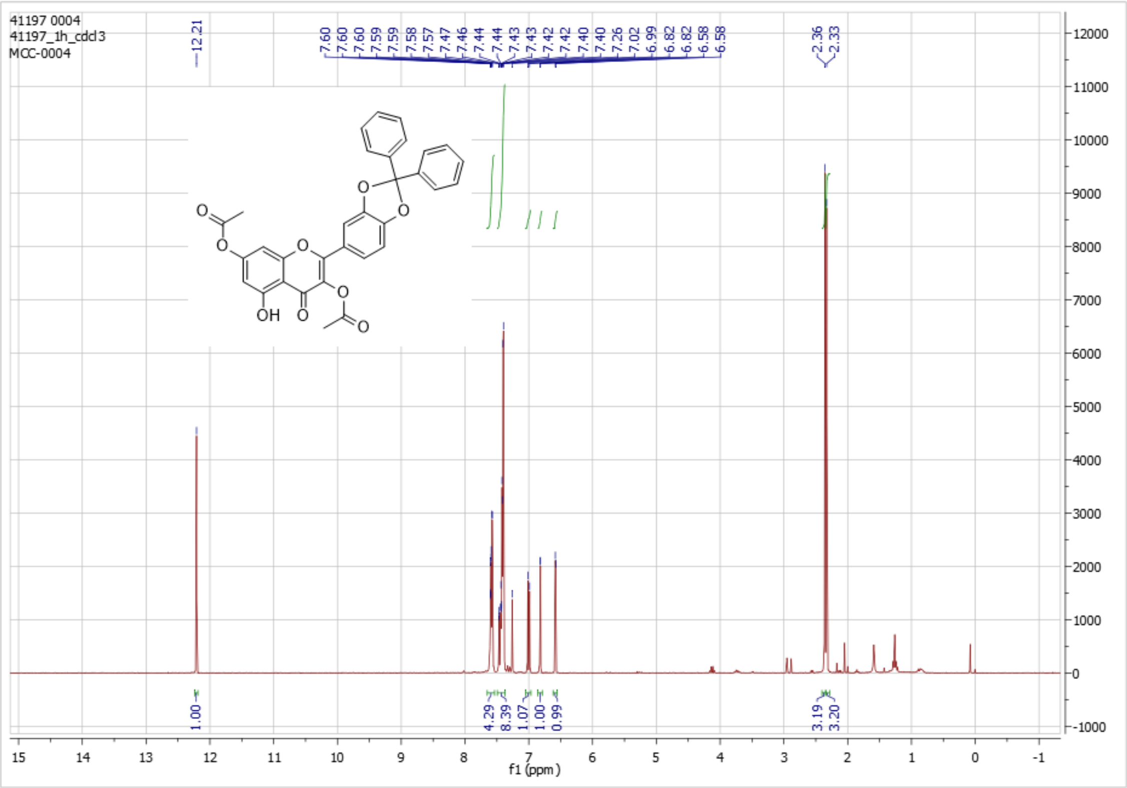

Figure S2. ^{1}H NMR (300 MHz, $CDCl_3$) of 3′,4′-*O*-diphenylmethane-3,7-diacetyl quercetin.

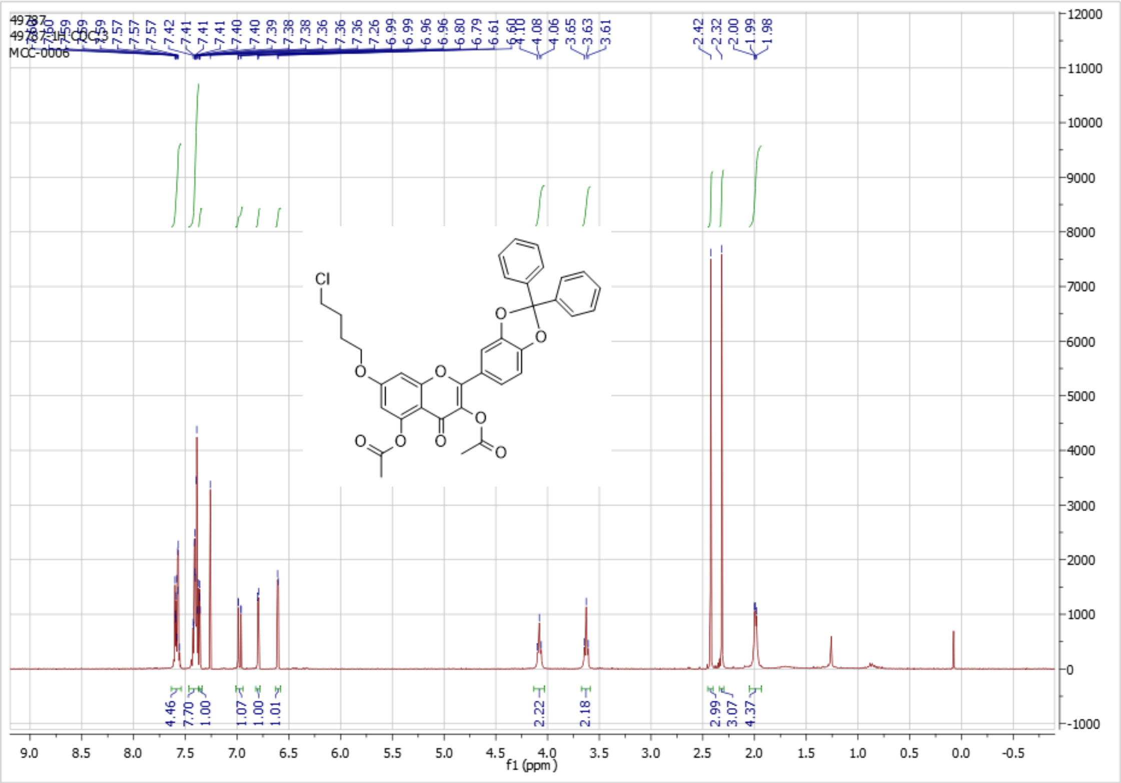

Figure S3. ^{1}H NMR (300 MHz, $CDCl_3$) of 7-(4-*O*-chlorobutyl)-3′,4′-*O*-diphenylmethane-3,5-diacetyl quercetin.

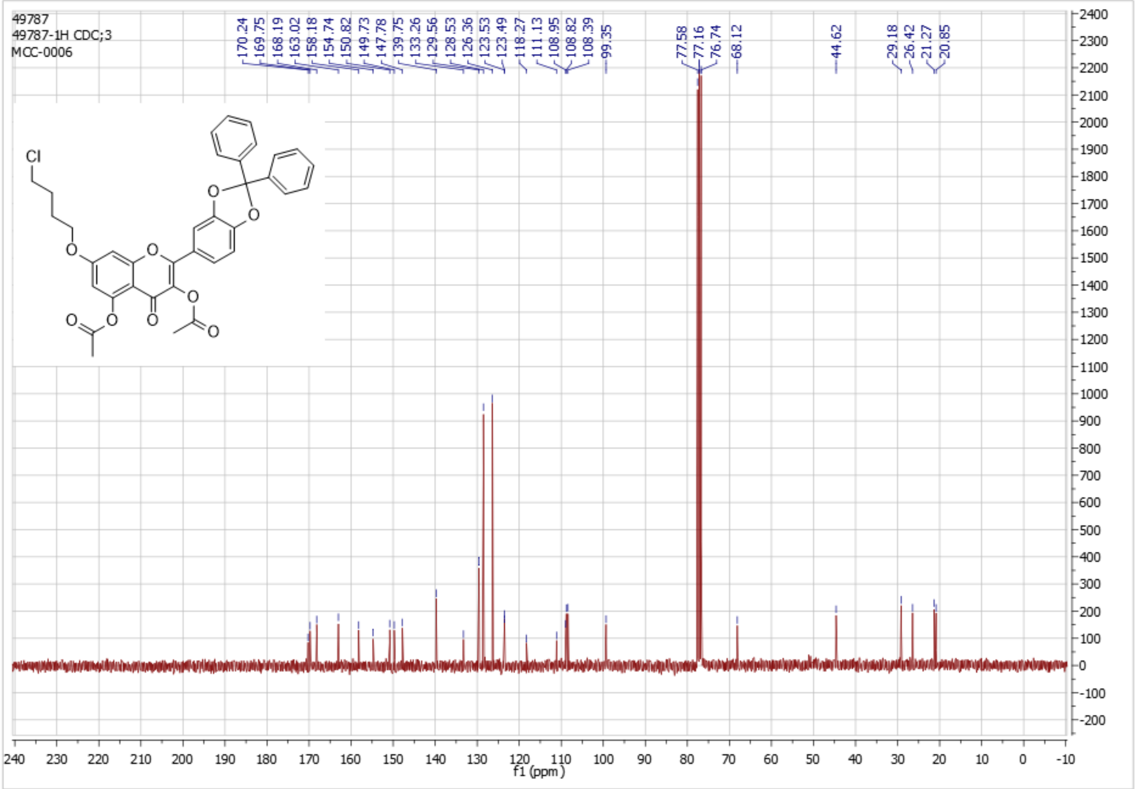

Figure S4. ^{13}C NMR (75 MHz, $CDCl_3$) of 7-(4-*O*-chlorobutyl)-3′,4′-*O*-diphenylmethane-3,5-diacetyl quercetin.

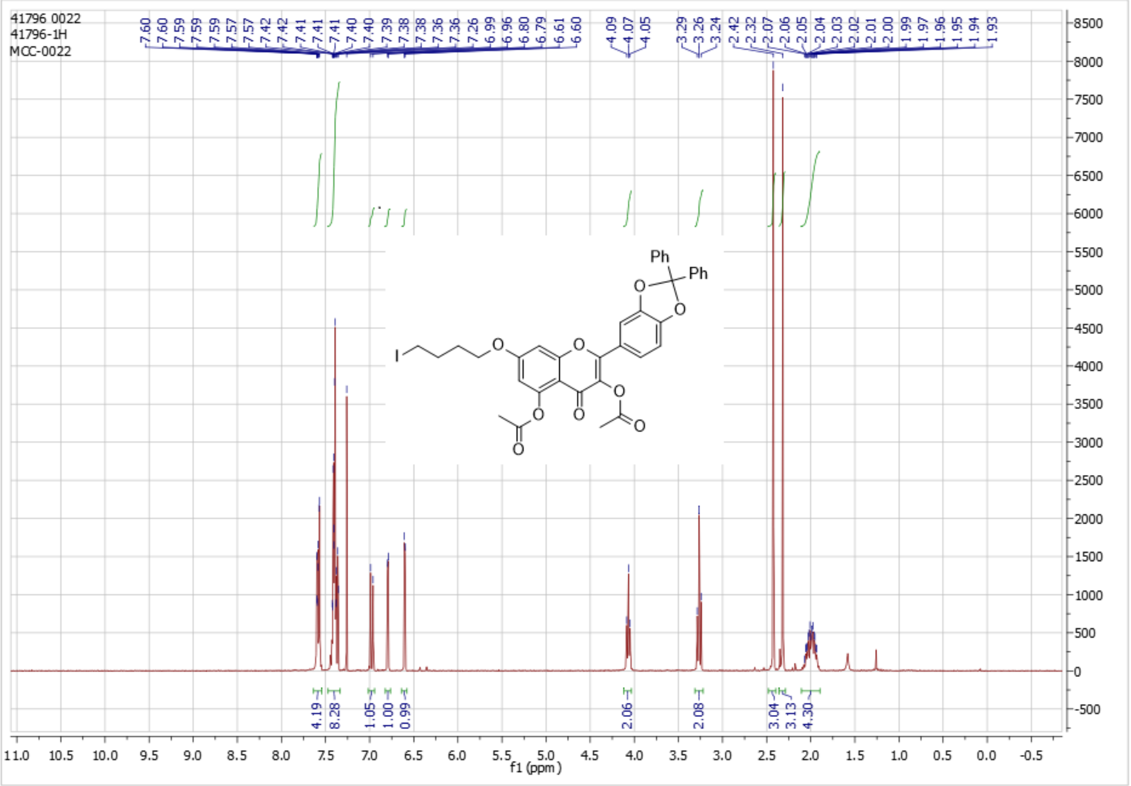

Figure S5. ^{1}H NMR (300 MHz, $CDCl_3$) of 7-(4-*O*-iodobutyl)-3′,4′-*O*-diphenylmethane-3,5-diacetyl quercetin.

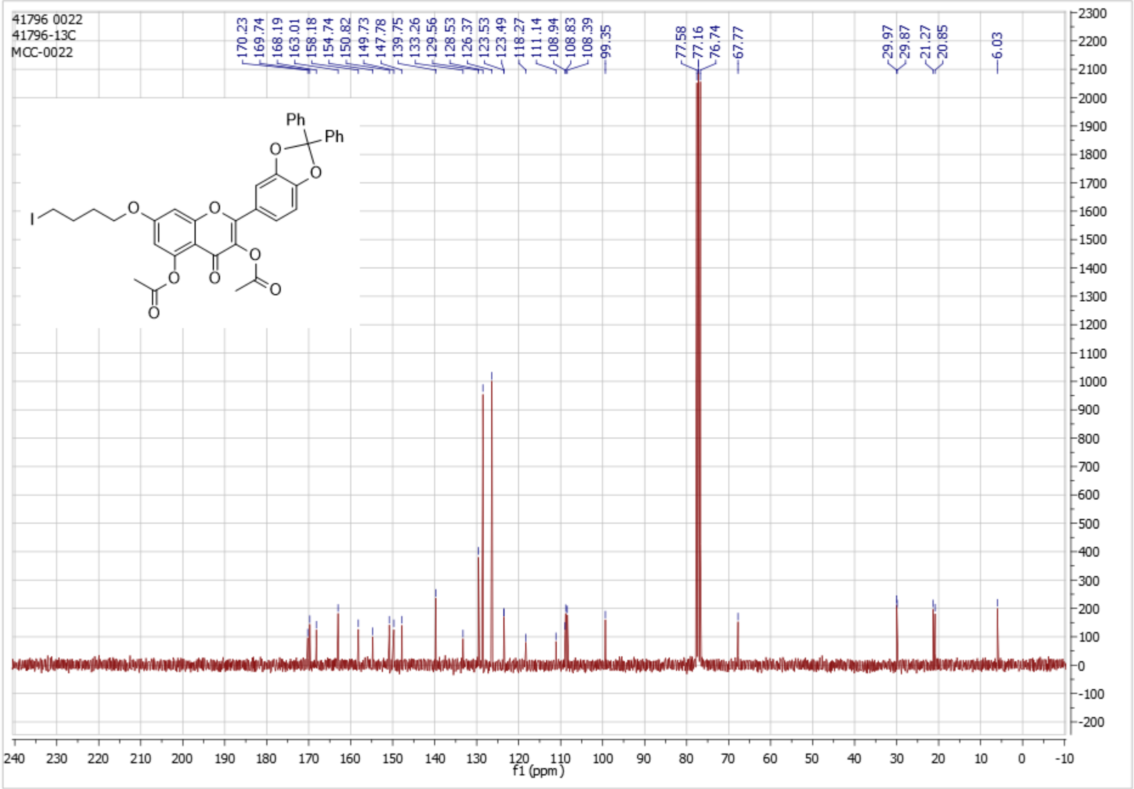

Figure S6. ^{13}C NMR (75 MHz, $CDCl_3$) of 7-(4-*O*-iodobutyl)-3′,4′-*O*-diphenylmethane-3,5-diacetyl quercetin.

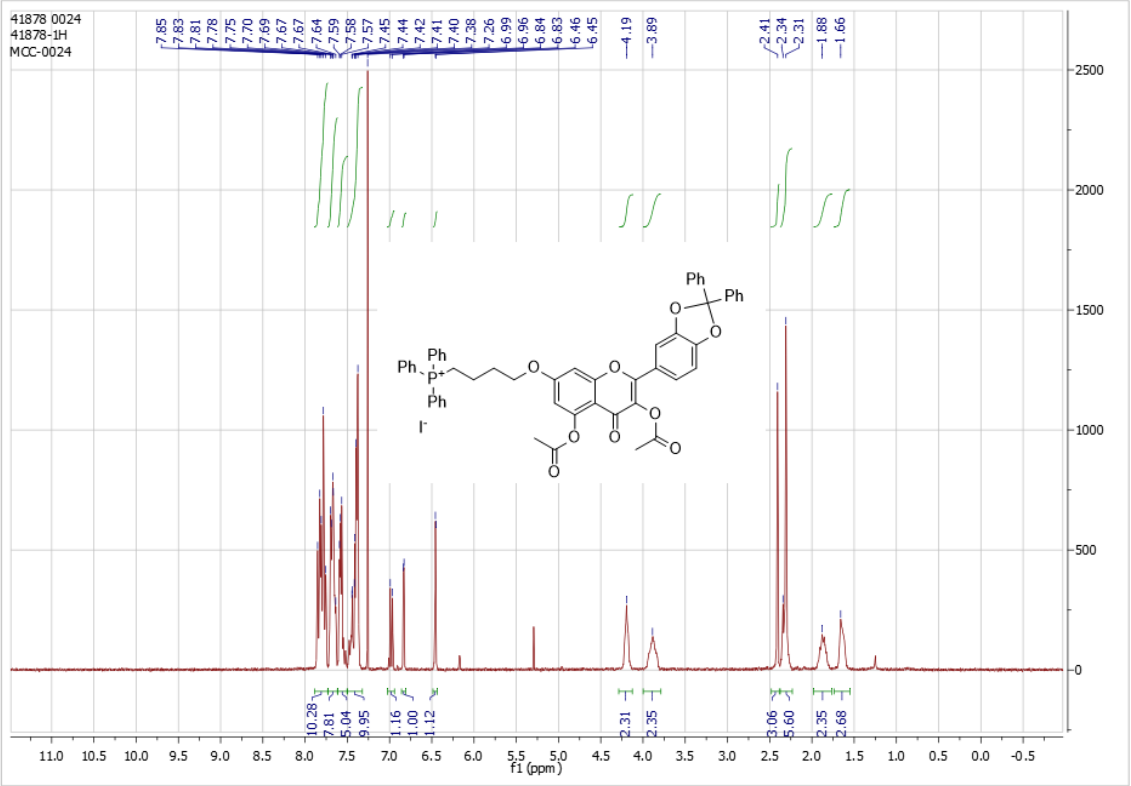

Figure S7. ^{1}H NMR (300 MHz, $CDCl_3$) of 7-(4-*O*-butyl triphenylphosphonium)-3′,4′-*O*-diphenylmethane-3,5-diacetyl quercetin iodide.

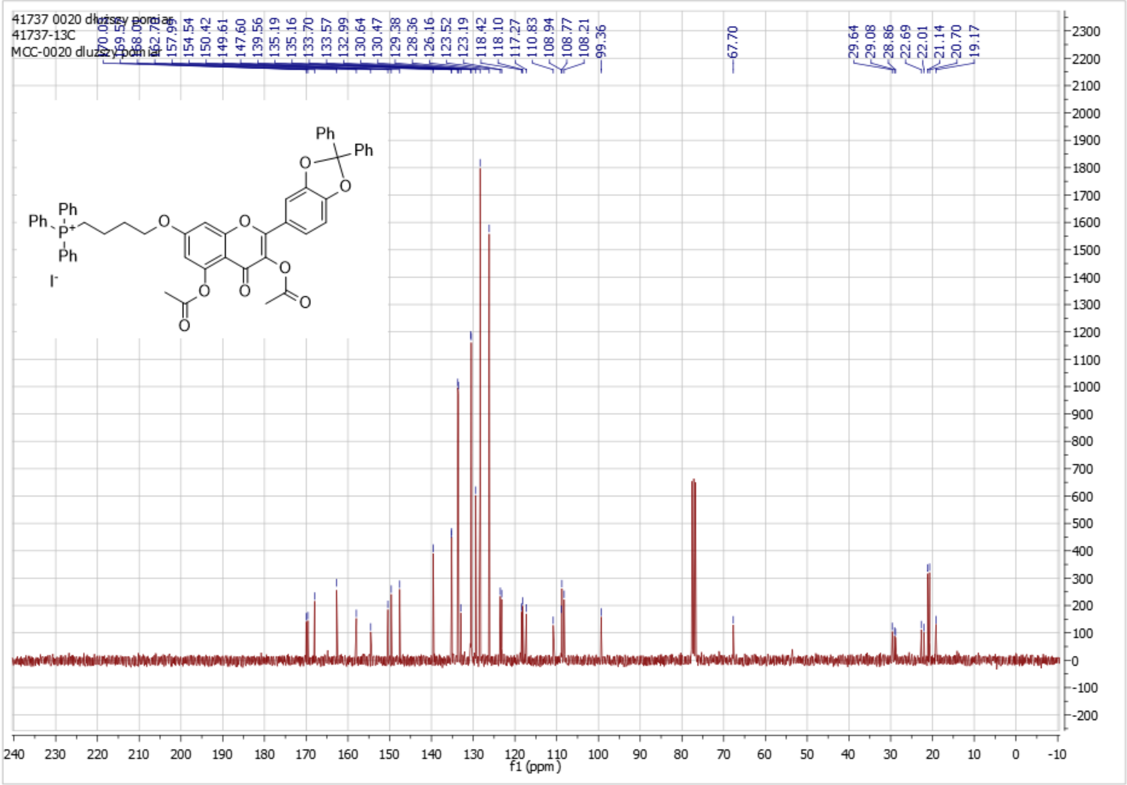

Figure S8. ^{13}C NMR (75 MHz, $CDCl_3$) of 7-(4-*O*-butyl triphenylphosphonium)-3′,4′-*O*-diphenylmethane-3,5-diacetyl quercetin iodide.

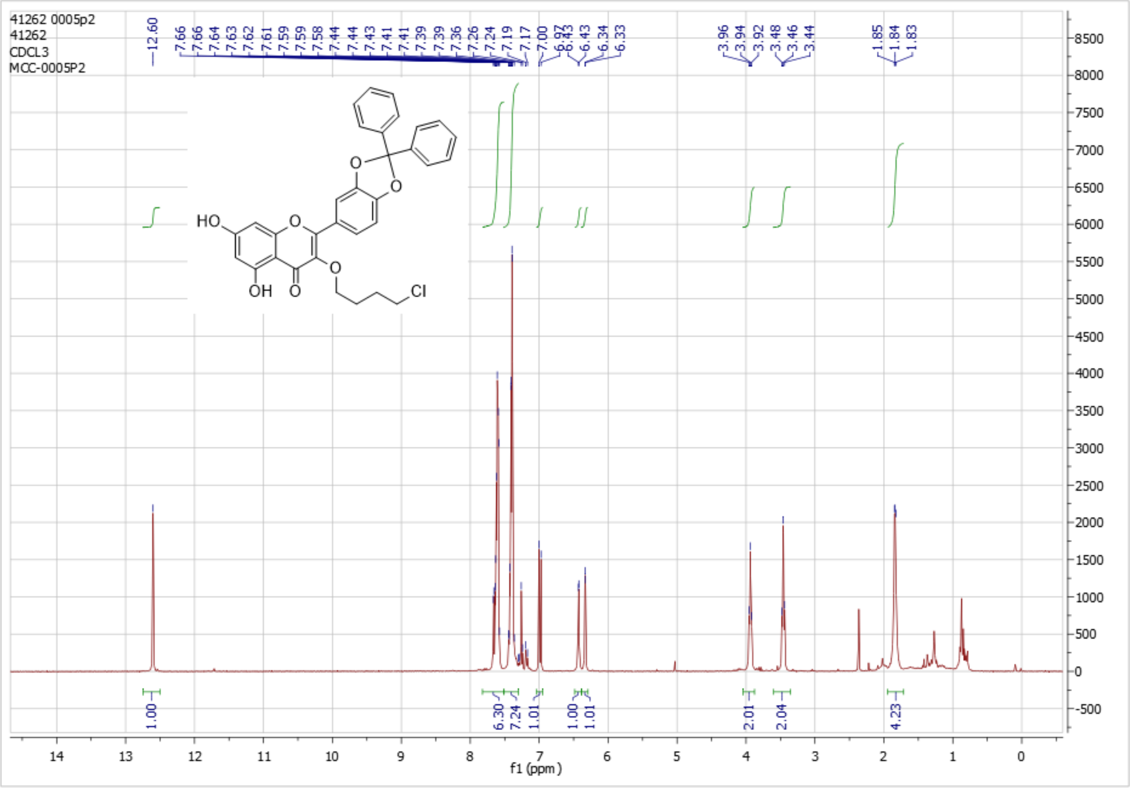

Figure S9. ^{1}H NMR (300 MHz, $CDCl_3$) of 3-(4-*O*-chlorobutyl)-3′,4′-*O*-diphenylmethane quercetin.

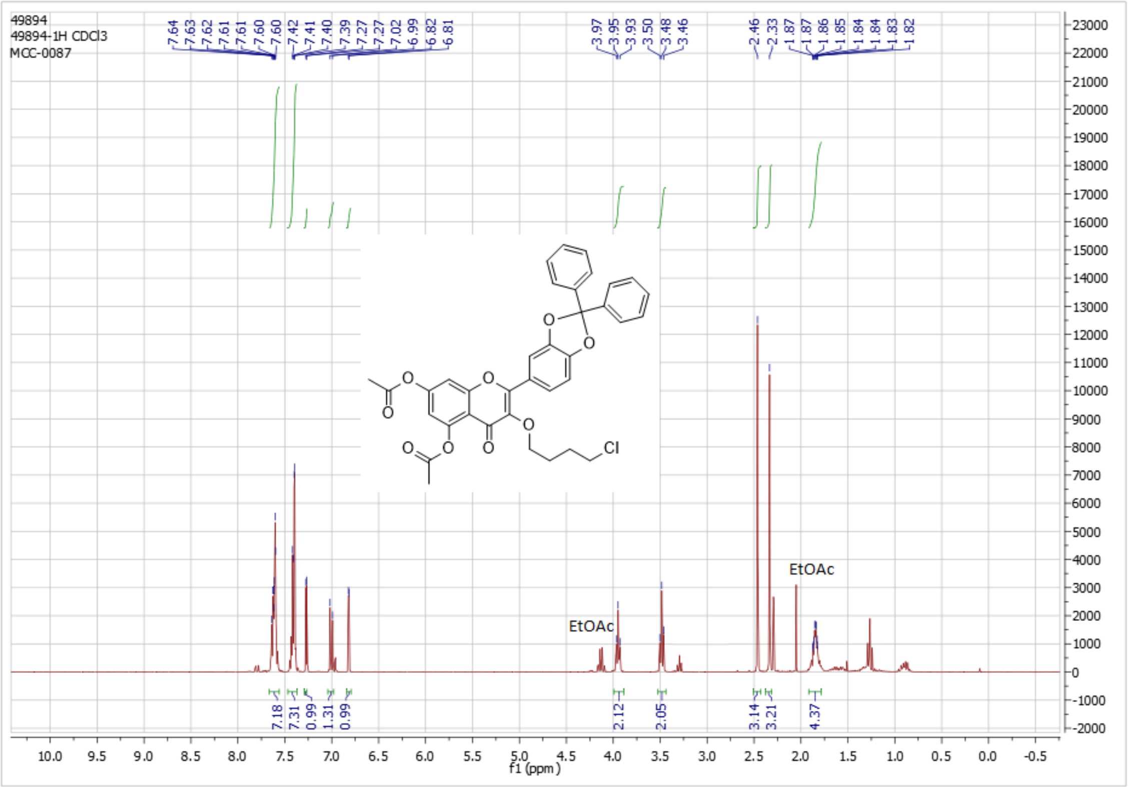

Figure S10. ^{1}H NMR (300 MHz, $CDCl_3$) of 3-(4-*O*-chlorobutyl)-3′,4′-*O*-diphenylmethane-5,7-diacetyl quercetin.

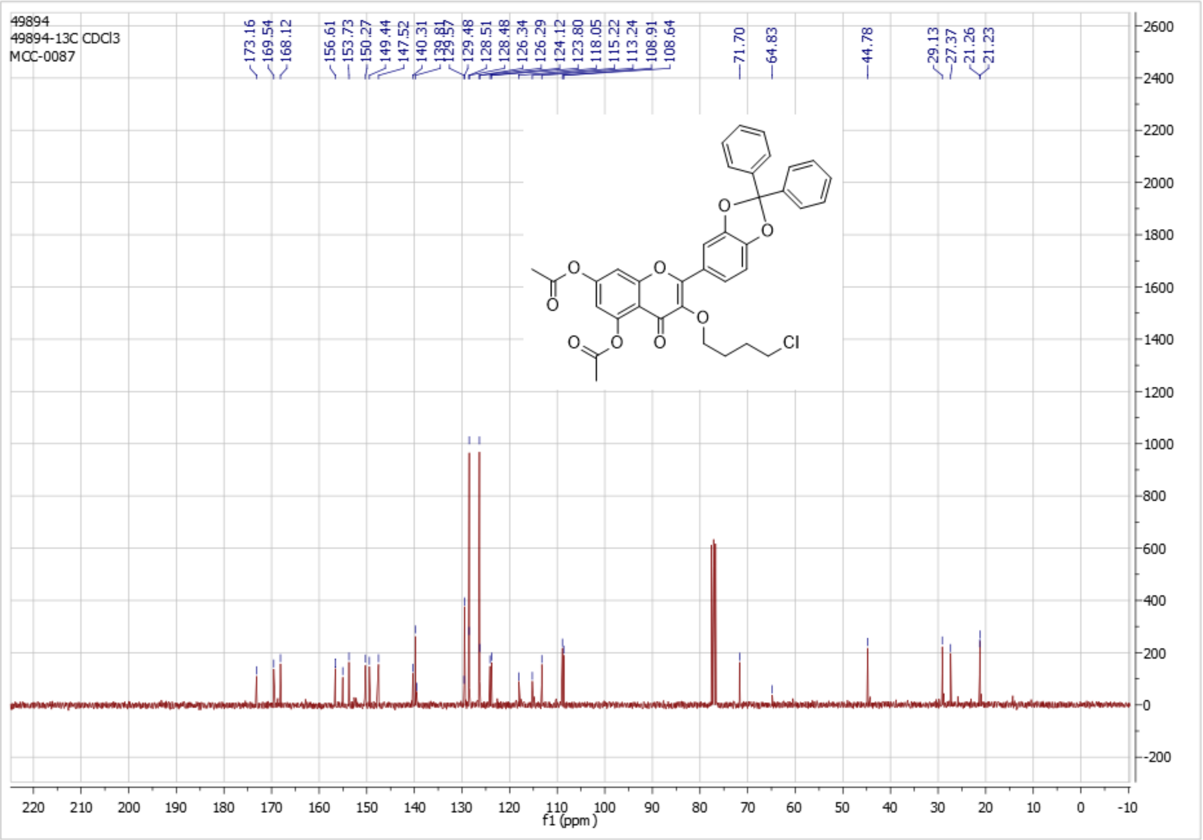

Figure S11. ^{13}C NMR (75 MHz, $CDCl_3$) of 3-(4-*O*-chlorobutyl)-3′,4′-*O*-diphenylmethane-5,7-diacetyl quercetin.

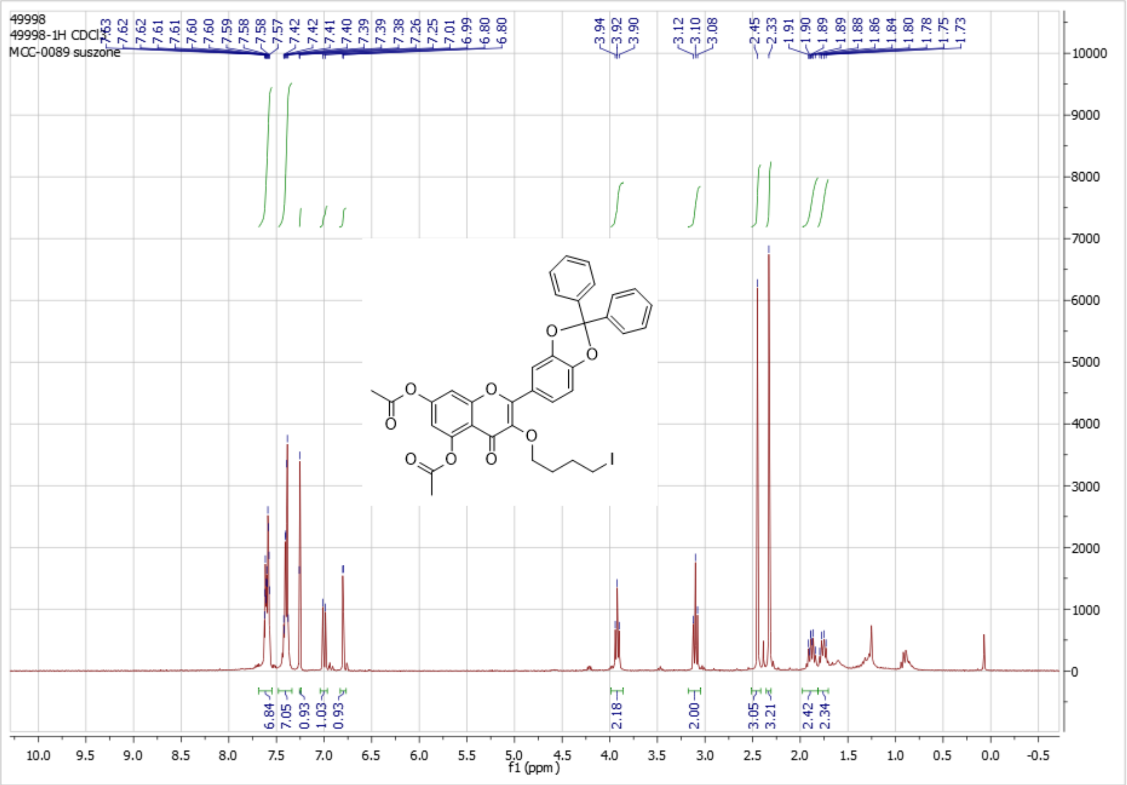

Figure S12. ^{1}H NMR (300 MHz, $CDCl_3$) of 3-(4-*O*-iodobutyl)-3′,4′-*O*-diphenylmethane-5,7-diacetyl quercetin.

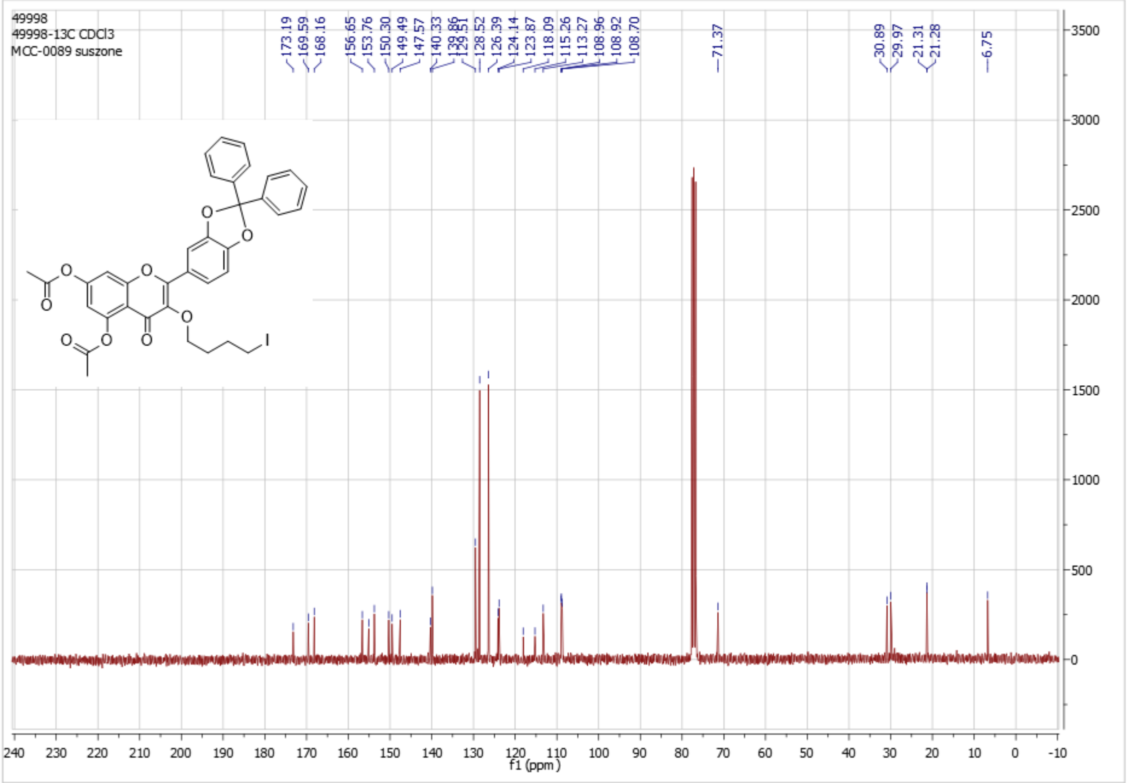

Figure S13. ^{13}C NMR (300 MHz, $CDCl_3$) of 3-(4-*O*-iodobutyl)-3′,4′-*O*-diphenylmethane-5,7-diacetyl quercetin.

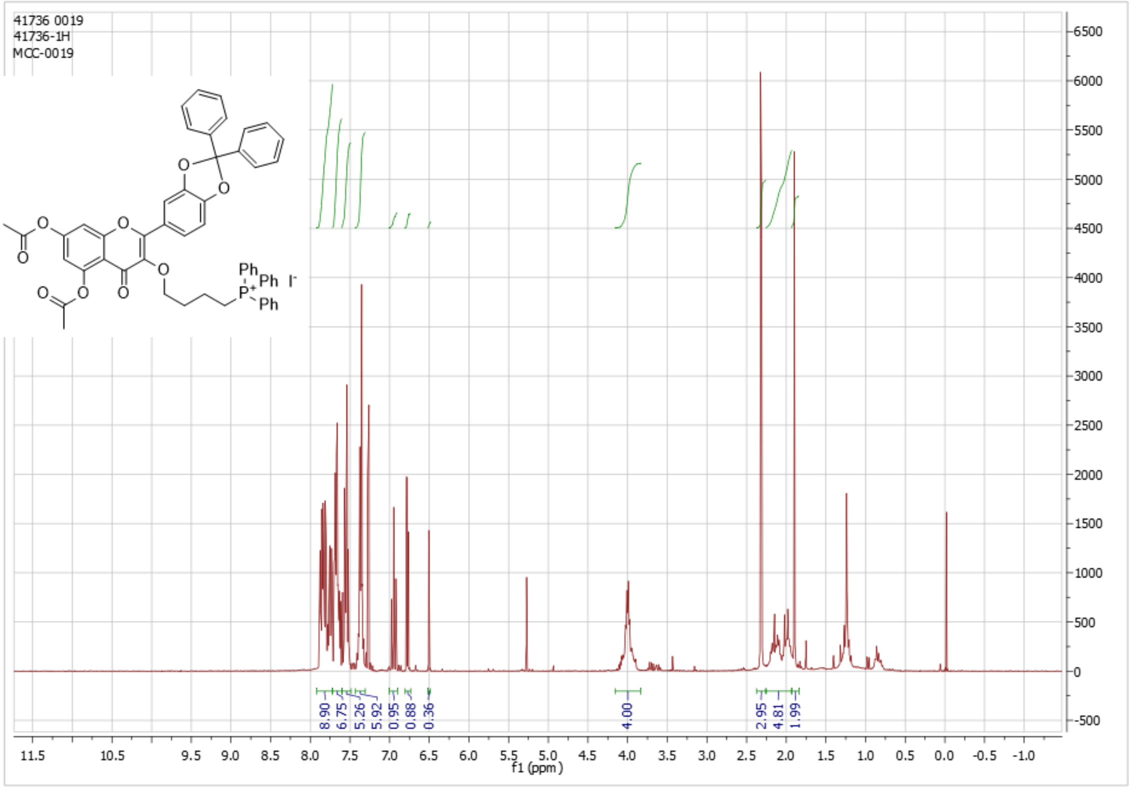

Figure S14. ^{1}H NMR (300 MHz, $CDCl_3$) of 3-(4-*O*-butyl triphenylphosphonium)-3′,4′-*O*-diphenylmethane-5,7-diacetyl quercetin iodide.

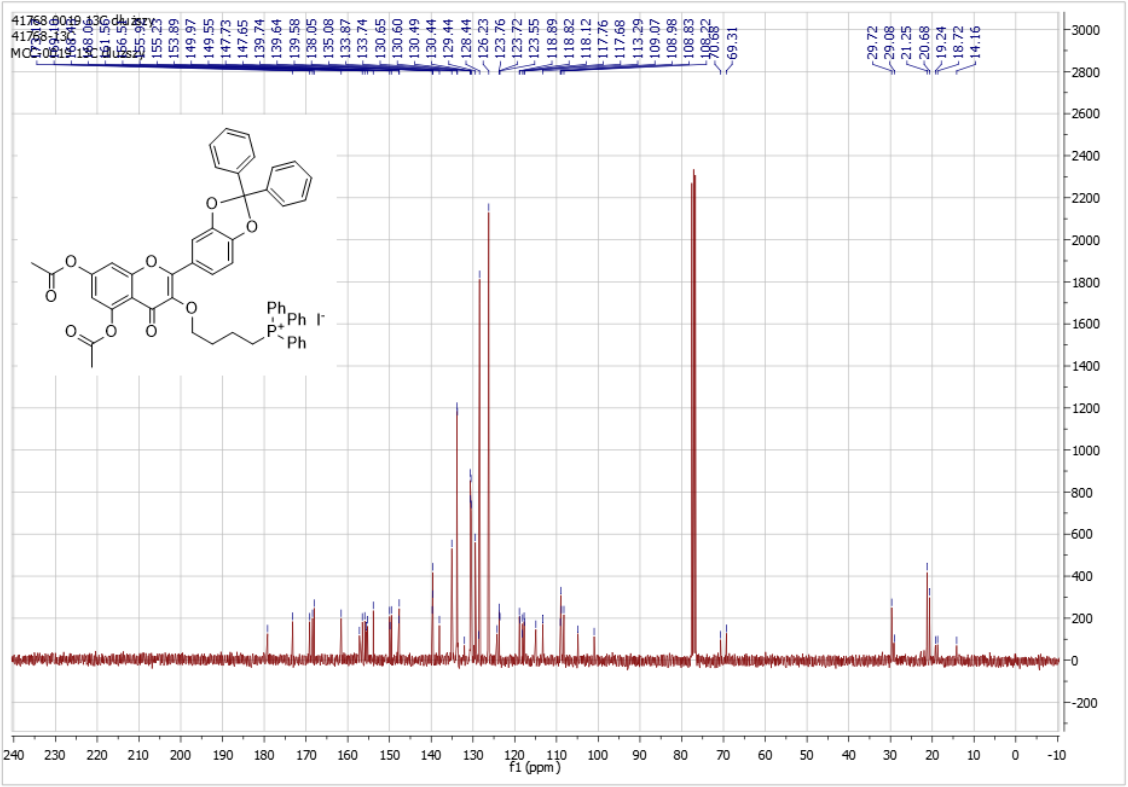

Figure S15. ^{13}C NMR (75 MHz, $CDCl_3$) of 3-(4-*O*-butyl triphenylphosphonium)-3′,4′-*O*-diphenylmethane-5,7-diacetyl quercetin iodide.

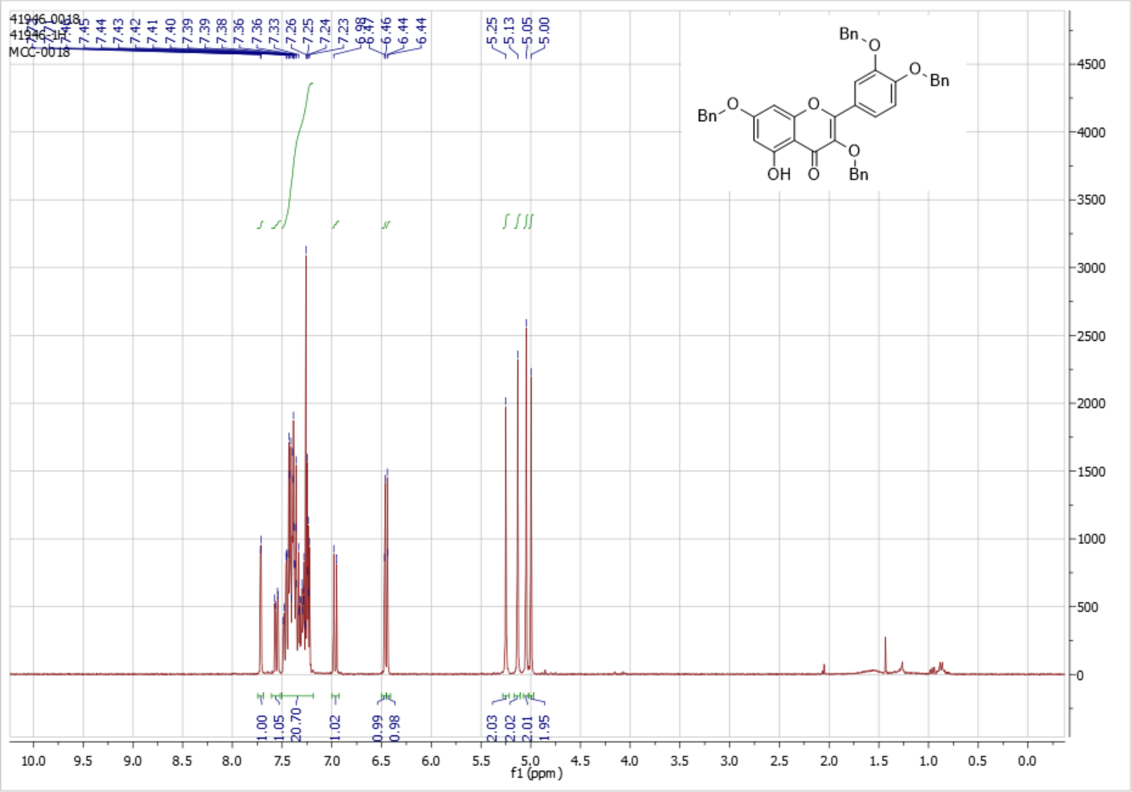

Figure S16. ^{1}H NMR (300 MHz, $CDCl_3$) of 3,3′,4′,7-tetrabenzyl quercetin.

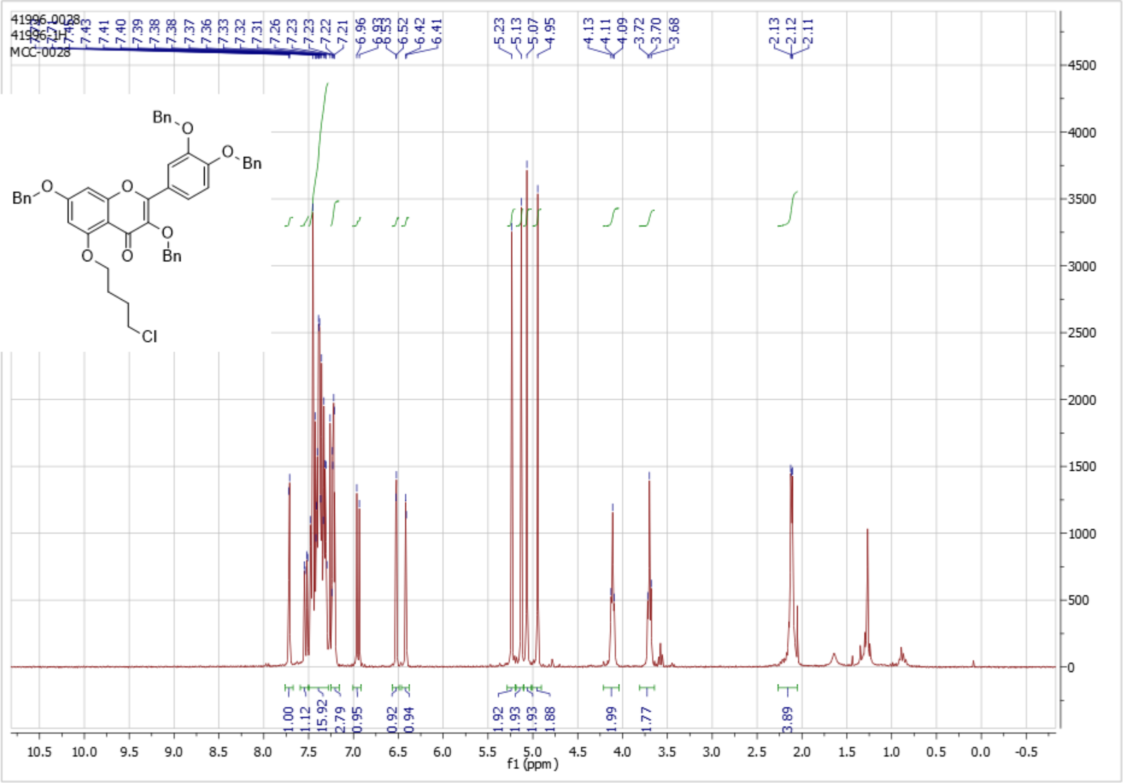

Figure S17. ^{1}H NMR (300 MHz, $CDCl_3$) of 3,3′,4′,7-tetrabenzyl-5-(4-*O*-chlorobutyl) quercetin.

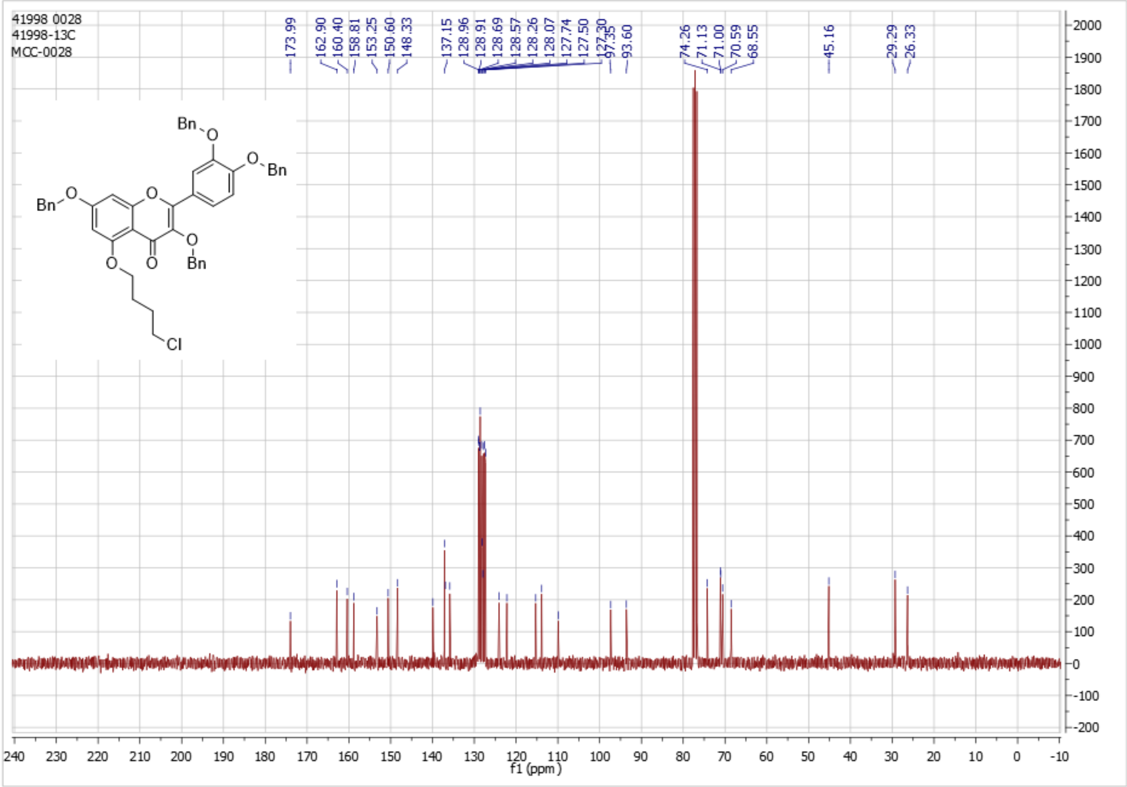

Figure S18. ^{13}C NMR (75 MHz, $CDCl_3$) of 3,3′,4′,7-tetrabenzyl-5-(4-*O*-chlorobutyl) quercetin.

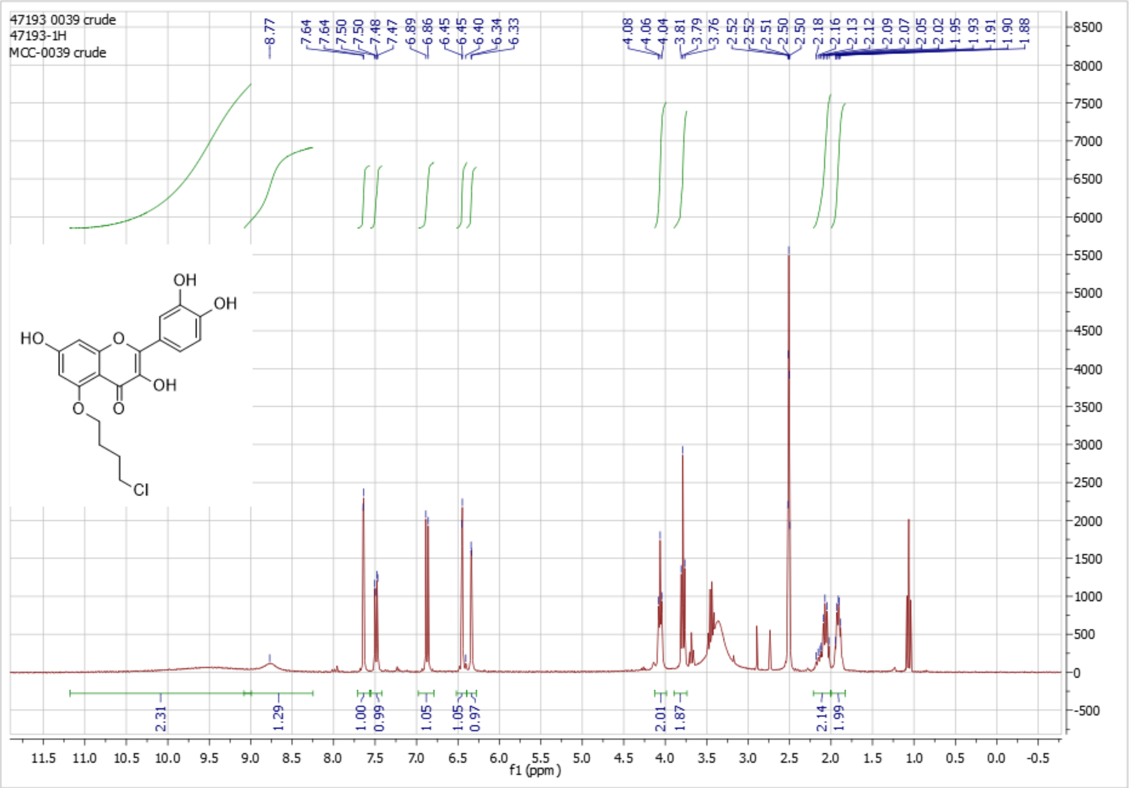

Figure S19. ^{1}H NMR (300 MHz, $CDCl_3$) of 5-(4-*O*-chlorobutyl) quercetin.

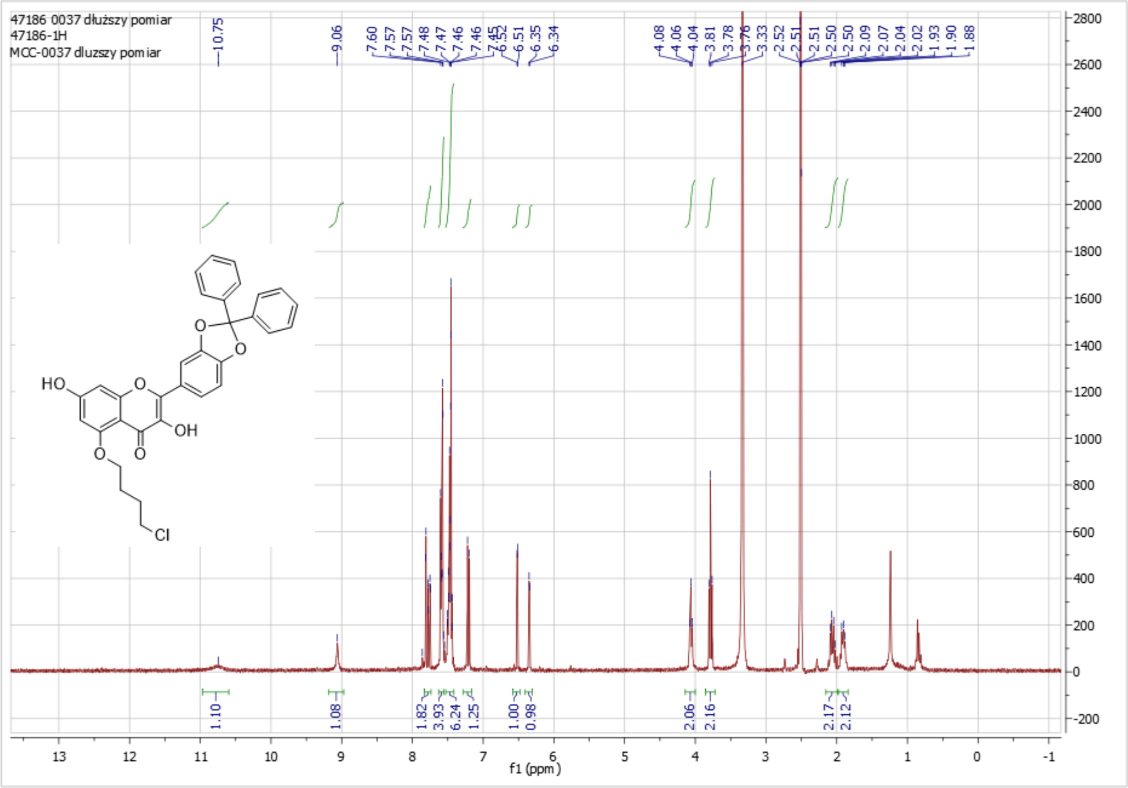

Figure S20. ^{1}H NMR (300 MHz, $CDCl_3$) of 5-(4-*O*-chlorobutyl)-3′,4′-*O*-diphenylmethane quercetin.

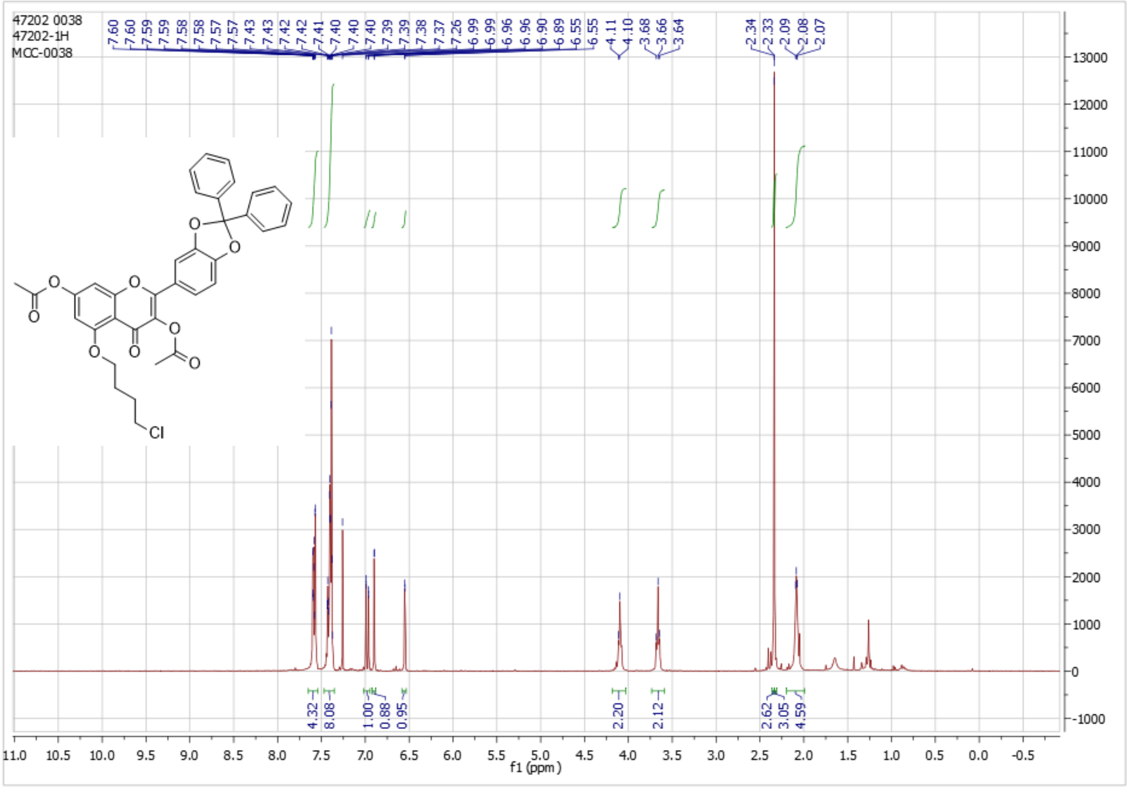

Figure S21. ^{1}H NMR (300 MHz, $CDCl_3$) of 5-(4-*O*-chlorobutyl)-3,7-diacetyl-3′,4′-*O*-diphenylmethane quercetin.

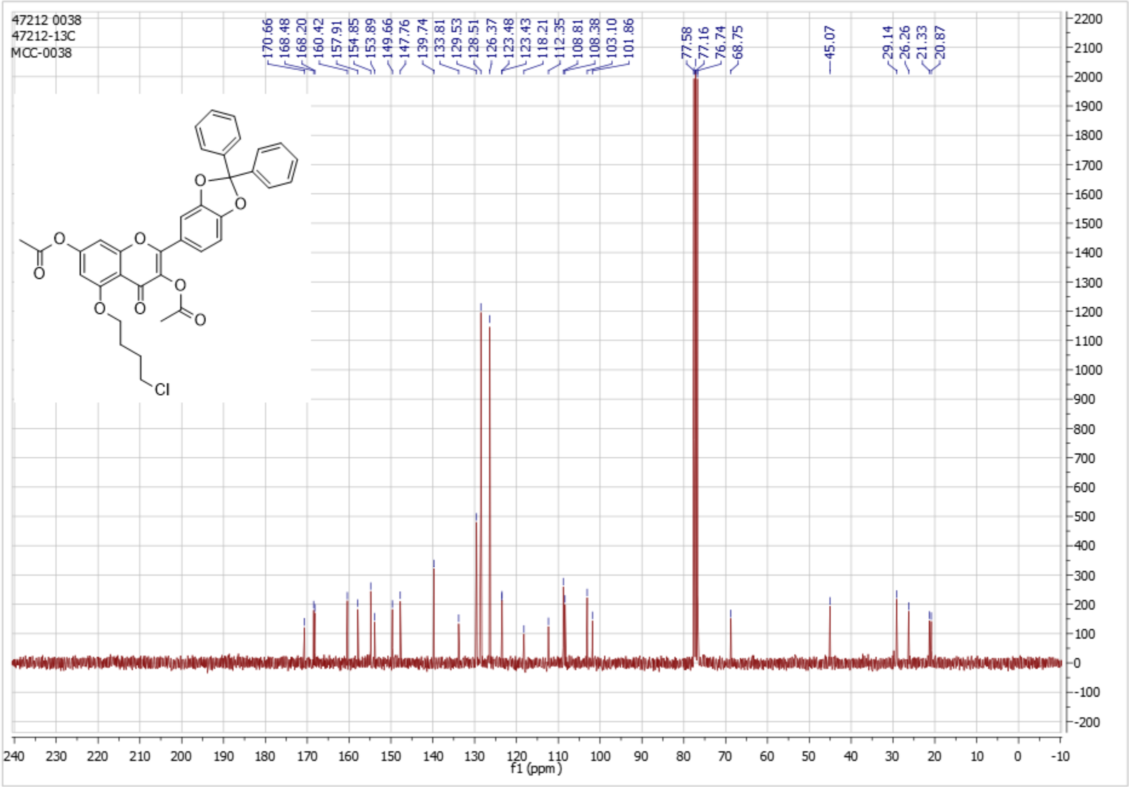

Figure S22. ^{13}C NMR (75 MHz, $CDCl_3$) of 5-(4-*O*-chlorobutyl)-3,7-diacetyl-3′,4′-*O*-diphenylmethane quercetin.

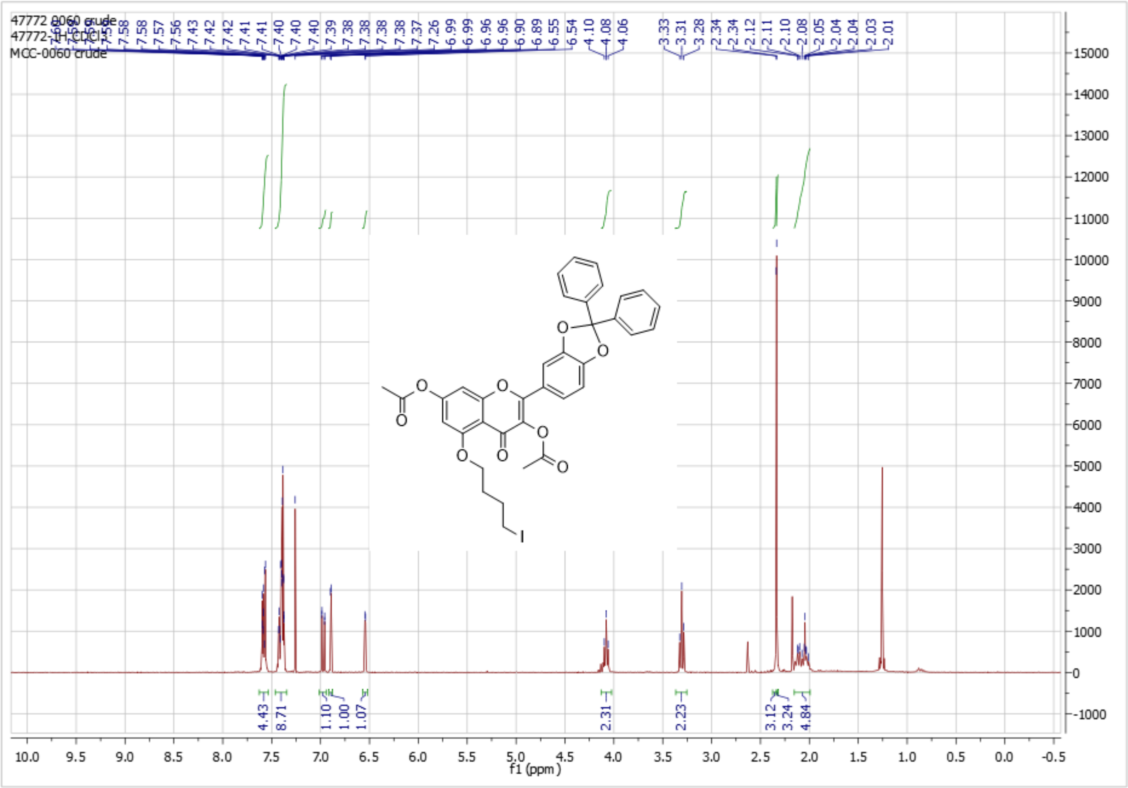

Figure S23. ^{1}H NMR (300 MHz, $CDCl_3$) of 5-(4-*O*-iodobutyl)-3,7-diacetyl-3′,4′-*O*-diphenylmethane quercetin.

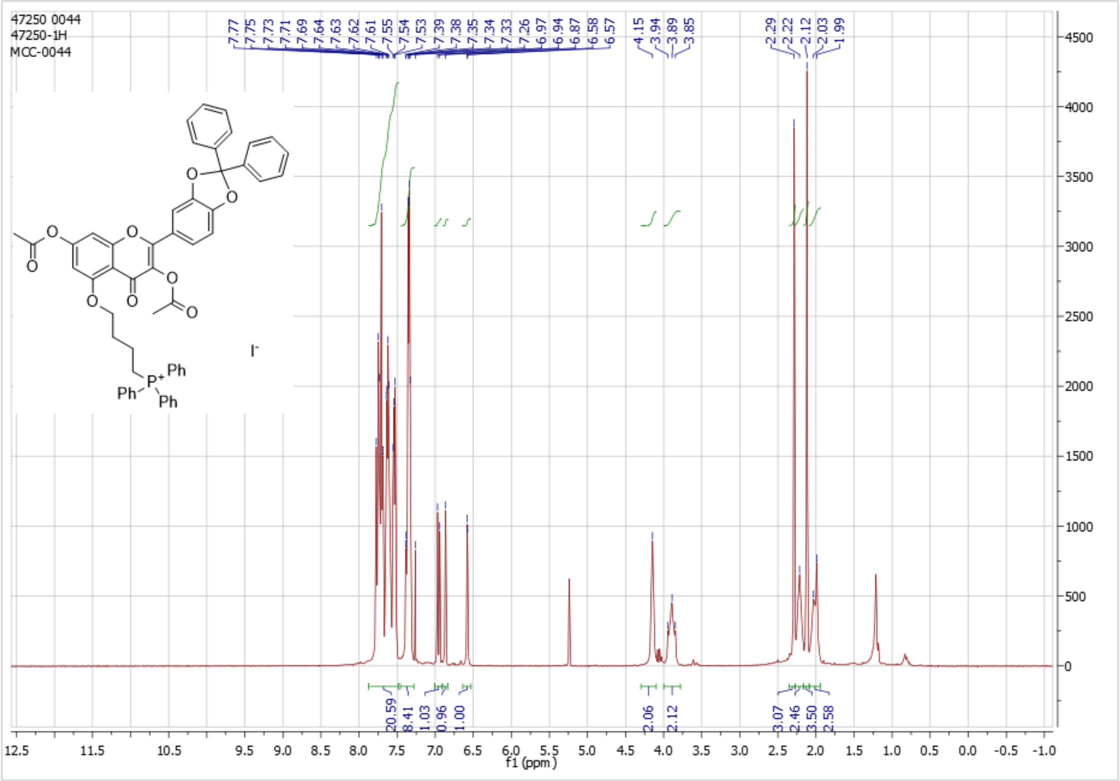

Figure S24. ^{1}H NMR (300 MHz, $CDCl_3$) of 5-(4-*O*-butyl triphenylphosphonium)-3′,4′-*O*-diphenylmethane-3,7-diacetyl quercetin iodide.

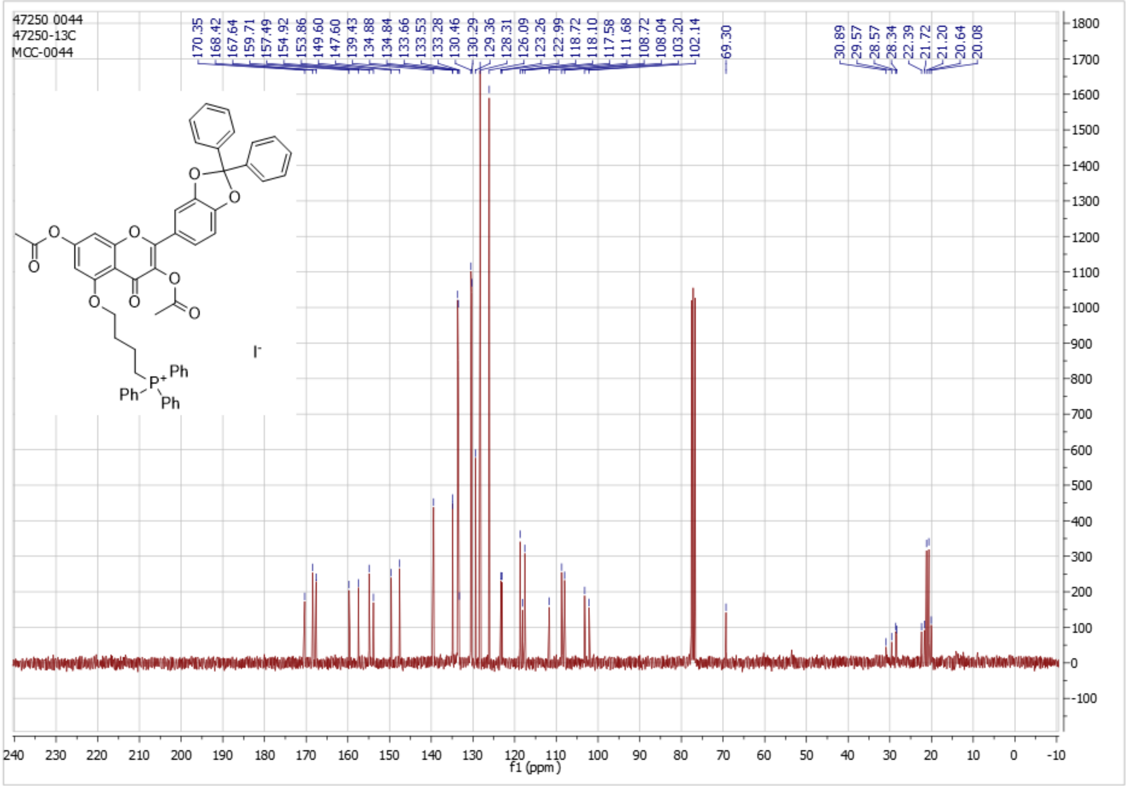

Figure S25. ^{13}C NMR (75 MHz, $CDCl_3$) of 5-(4-*O*-butyl triphenylphosphonium)-3′,4′-*O*-diphenylmethane-3,7-diacetyl quercetin iodide.

3. Kinetic measurements for quercetin

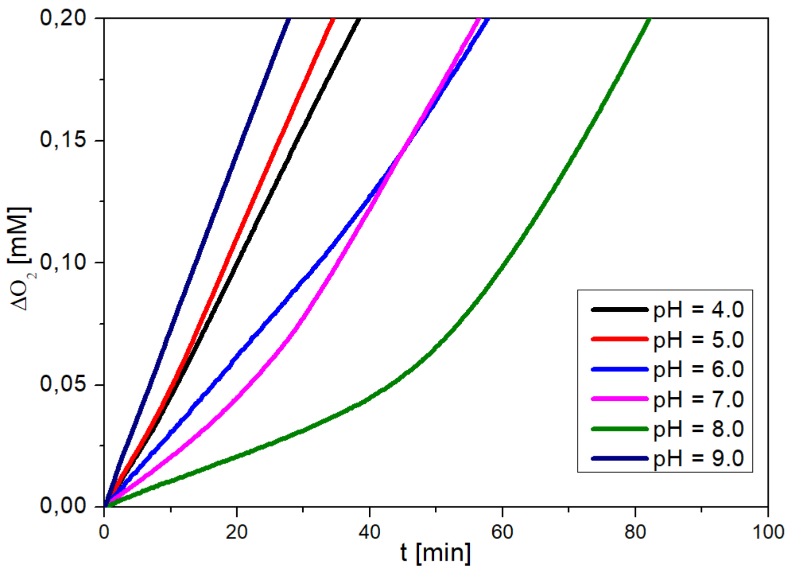

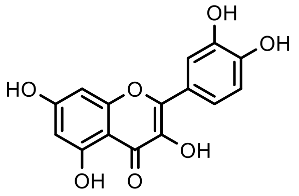

QUERCETIN

Figure S26. Kinetic traces of oxygen uptake for peroxidation in the presence of quercetin.

Table S1. Kinetic parameters: induction time τ_{ind}, rate of autoxidation, R_{ox1}, initiation rate R_i, rate of inhibited autoxidation R_{inh}, inhibition rate constant k_{inh} determined for autoxidation of 2.7 mM methyl linoleate in 10 mM DMPC liposomes in the presence of 1µM quercetin at pH range from 4 to 9.

pH	τ_{ind} [min]	$R_{ox1} \cdot 10^8$ [$M^{-1}s^{-1}$]	$R_i \cdot 10^8$ [$M^{-1}s^{-1}$]	$R_{inh} \cdot 10^8$ [$M^{-1}s^{-1}$]	$k_{inh} \cdot 10^4$ [$M^{-1}s^{-1}$]
4.0	-	16.6 ± 1.1	-	-	-
5.0	-	13.0 ± 0.1	-	-	-
6.0	-	15.6 ± 0.6	-	-	-
7.0	16.9 ± 1.2	15.8 ± 1.0	0.13 ± 0.02	4.4 ± 0.3	0.8 ± 0.3
8.0	46.8 ± 3.2	14.5 ± 1.5	0.04 ± 0.01	2.8 ± 0.1	0.4 ± 0.1
9.0	-	13.8 ± 0.6	-	-	-

Table S2. Thermodynamic parameters determined from DSC experiments: molar enthalpy (Δ*H* in kJ/mol) and the main transition temperature[a] (T_m in kelvins) of DMPC containing quercetin and its derivatives mitQ3/5/7 at the concentrations of 0, 50, 100, and 150 µM.

C [µM]	quercetin		mitQ3		mitQ5		mitQ7	
	Δ*H*	T_m	Δ*H*	T_m	Δ*H*	T_m	Δ*H*	T_m
0	20.5	297.1	20.5	297.1	20.5	297.1	20.5	297.1
50	nd[b]	nd[b]	16.6	296.7	18.8	296.7	19.9	296.7
100	nd[b]	nd[b]	16.0	296.4	17.6	296.3	15.1	296.4
150	14.2	296.9	12.6	296.0	13.9	296.4	13.5	296.1

[a] Temperature at which 50% of heat was exchanged. [b] nd = not determined.

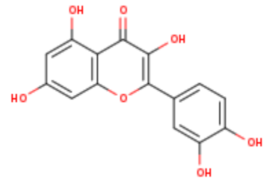

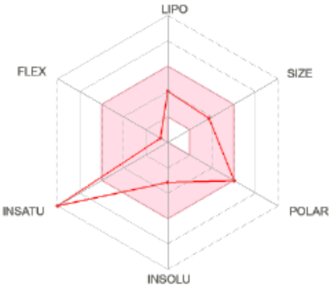

SMILES Oc1cc(O)c2c(c1)oc(c(c2=O)O)c1ccc(c(c1)O)O

Physicochemical Properties	
Formula	C15H10O7
Molecular weight	302.24 g/mol
Num. heavy atoms	22
Num. arom. heavy atoms	16
Fraction Csp3	0.00
Num. rotatable bonds	1
Num. H-bond acceptors	7
Num. H-bond donors	5
Molar Refractivity	78.03
TPSA	131.36 Å²

Lipophilicity	
Log $P_{o/w}$ (iLOGP)	1.63
Log $P_{o/w}$ (XLOGP3)	1.54
Log $P_{o/w}$ (WLOGP)	1.99
Log $P_{o/w}$ (MLOGP)	-0.56
Log $P_{o/w}$ (SILICOS-IT)	1.54
Consensus Log $P_{o/w}$	1.23

Water Solubility	
Log *S* (ESOL)	-3.16
Solubility	2.11e-01 mg/ml ; 6.98e-04 mol/l
Class	Soluble
Log *S* (Ali)	-3.91
Solubility	3.74e-02 mg/ml ; 1.24e-04 mol/l
Class	Soluble
Log *S* (SILICOS-IT)	-3.24
Solubility	1.73e-01 mg/ml ; 5.73e-04 mol/l
Class	Soluble

Pharmacokinetics	
GI absorption	High
BBB permeant	No
P-gp substrate	No
CYP1A2 inhibitor	Yes
CYP2C19 inhibitor	No
CYP2C9 inhibitor	No
CYP2D6 inhibitor	Yes
CYP3A4 inhibitor	Yes
Log K_p (skin permeation)	-7.05 cm/s

Druglikeness	
Lipinski	Yes; 0 violation
Ghose	Yes
Veber	Yes
Egan	Yes
Muegge	Yes
Bioavailability Score	0.55

Medicinal Chemistry	
PAINS	1 alert: catechol_A
Brenk	1 alert: catechol
Leadlikeness	Yes
Synthetic accessibility	3.23

Figure S27. ADME parameters calculated for quercetin.

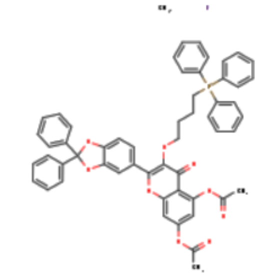

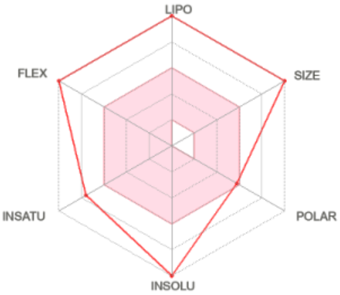

SMILES	CC(=O)Oc1cc(OC(=O)C)c2c(c1)oc(c(c2=O)OCCCC[P+](c1ccccc1)(c1ccccc1)c1ccccc1)c1ccc2c(c1)OC(O2)(c1ccccc1)c1ccccc1.C.[I-]
**Physicochemical Properties**	
Formula	C55H48IO9P
Molecular weight	1010.84 g/mol
Num. heavy atoms	66
Num. arom. heavy atoms	46
Fraction Csp3	0.15
Num. rotatable bonds	16
Num. H-bond acceptors	9
Num. H-bond donors	0
Molar Refractivity	271.78
TPSA	124.09 Å²
**Lipophilicity**	
Log $P_{o/w}$ (iLOGP)	0.00
Log $P_{o/w}$ (XLOGP3)	12.31
Log $P_{o/w}$ (WLOGP)	7.67
Log $P_{o/w}$ (MLOGP)	6.54
Log $P_{o/w}$ (SILICOS-IT)	11.45
Consensus Log $P_{o/w}$	7.59

**Water Solubility**	
Log *S* (ESOL)	-13.32
Solubility	4.81e-11 mg/ml ; 4.76e-14 mol/l
Class	Insoluble
Log *S* (Ali)	-14.93
Solubility	1.18e-12 mg/ml ; 1.17e-15 mol/l
Class	Insoluble
Log *S* (SILICOS-IT)	-19.37
Solubility	4.33e-17 mg/ml ; 4.29e-20 mol/l
Class	Insoluble
**Pharmacokinetics**	
GI absorption	Low
BBB permeant	No
P-gp substrate	Yes
CYP1A2 inhibitor	No
CYP2C19 inhibitor	No
CYP2C9 inhibitor	No
CYP2D6 inhibitor	No
CYP3A4 inhibitor	Yes
Log K_p (skin permeation)	-3.73 cm/s
**Druglikeness**	
Lipinski	No; 2 violations: MW>500, MLOGP>4.15
Ghose	No; 4 violations: MW>480, WLOGP>5.6, MR>130, #atoms>70
Veber	No; 1 violation: Rotors>10
Egan	No; 1 violation: WLOGP>5.88
Muegge	No; 4 violations: MW>600, XLOGP3>5, #rings>7, Rotors>15
Bioavailability Score	0.17
**Medicinal Chemistry**	
PAINS	0 alert
Brenk	3 alerts: iodine, phenol_ester, phosphor
Leadlikeness	No; 3 violations: MW>350, Rotors>7, XLOGP3>3.5
Synthetic accessibility	6.82

Figure S28. ADME parameters calculated for mitQ3.

Please note that consensus log$P_{o/w}$ for mitQ3 was erroneously calculated as an average from 5 values, while one of them (ilogP=0.00) should be omitted, thus, consensus log$P_{o/w}$ is 9.49.

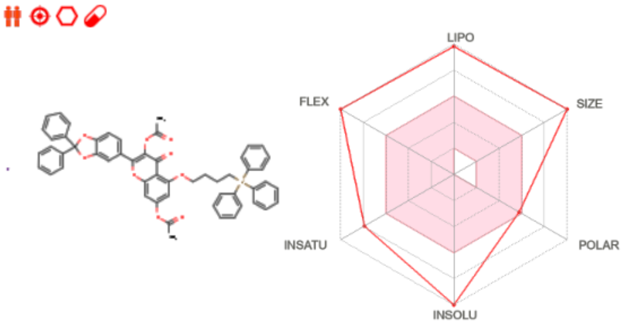

SMILES	CC(=O)Oc1cc(OCCCC[P+](c2ccccc2)(c2ccccc2)c2ccccc2)c2c(c1)oc(c(c2=O)OC(=O)C)c1ccc2c(c1)OC(O2)(c1ccccc1)c1ccccc1.[I-]
**Physicochemical Properties**	
Formula	C54H44IO9P
Molecular weight	994.80 g/mol
Num. heavy atoms	65
Num. arom. heavy atoms	46
Fraction Csp3	0.13
Num. rotatable bonds	16
Num. H-bond acceptors	9
Num. H-bond donors	0
Molar Refractivity	264.86
TPSA	124.09 Å²
**Lipophilicity**	
Log $P_{o/w}$ (iLOGP)	8.58
Log $P_{o/w}$ (XLOGP3)	11.66
Log $P_{o/w}$ (WLOGP)	7.03
Log $P_{o/w}$ (MLOGP)	6.40
Log $P_{o/w}$ (SILICOS-IT)	11.45
Consensus Log $P_{o/w}$	9.02

**Water Solubility**	
Log *S* (ESOL)	-12.82
Solubility	1.50e-10 mg/ml ; 1.51e-13 mol/l
Class	Insoluble
Log *S* (Ali)	-14.26
Solubility	5.51e-12 mg/ml ; 5.54e-15 mol/l
Class	Insoluble
Log *S* (SILICOS-IT)	-19.37
Solubility	4.26e-17 mg/ml ; 4.29e-20 mol/l
Class	Insoluble
**Pharmacokinetics**	
GI absorption	Low
BBB permeant	No
P-gp substrate	Yes
CYP1A2 inhibitor	No
CYP2C19 inhibitor	No
CYP2C9 inhibitor	No
CYP2D6 inhibitor	No
CYP3A4 inhibitor	Yes
Log K_p (skin permeation)	-4.09 cm/s
**Druglikeness**	
Lipinski	No; 2 violations: MW>500, MLOGP>4.15
Ghose	No; 4 violations: MW>480, WLOGP>5.6, MR>130, #atoms>70
Veber	No; 1 violation: Rotors>10
Egan	No; 1 violation: WLOGP>5.88
Muegge	No; 4 violations: MW>600, XLOGP3>5, #rings>7, Rotors>15
Bioavailability Score	0.17
**Medicinal Chemistry**	
PAINS	0 alert
Brenk	3 alerts: iodine, phenol_ester, phosphor
Leadlikeness	No; 3 violations: MW>350, Rotors>7, XLOGP3>3.5
Synthetic accessibility	6.57

Figure S29. ADME parameters calculated for mitQ5.

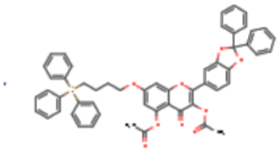

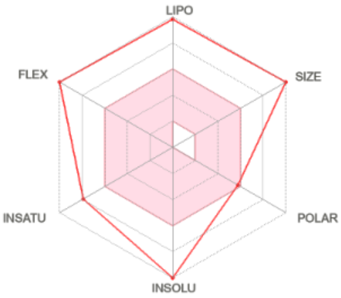

SMILES	CC(=O)Oc1c(oc2c(c1=O)c(OC(=O)C)cc(c2)OCCCC[P+](c1ccccc1)(c1ccccc1)c1ccccc1)c1ccc2c(c1)OC(O2)(c1ccccc1)c1ccccc1.[I-]

Physicochemical Properties	
Formula	C54H44IO9P
Molecular weight	994.80 g/mol
Num. heavy atoms	65
Num. arom. heavy atoms	46
Fraction Csp3	0.13
Num. rotatable bonds	16
Num. H-bond acceptors	9
Num. H-bond donors	0
Molar Refractivity	264.86
TPSA	124.09 Å²

Lipophilicity	
Log $P_{o/w}$ (iLOGP)	8.62
Log $P_{o/w}$ (XLOGP3)	11.66
Log $P_{o/w}$ (WLOGP)	7.03
Log $P_{o/w}$ (MLOGP)	6.40
Log $P_{o/w}$ (SILICOS-IT)	11.45
Consensus Log $P_{o/w}$	9.03

Water Solubility	
Log *S* (ESOL)	-12.82
Solubility	1.50e-10 mg/ml ; 1.51e-13 mol/l
Class	Insoluble
Log *S* (Ali)	-14.26
Solubility	5.51e-12 mg/ml ; 5.54e-15 mol/l
Class	Insoluble
Log *S* (SILICOS-IT)	-19.37
Solubility	4.26e-17 mg/ml ; 4.29e-20 mol/l
Class	Insoluble

Pharmacokinetics	
GI absorption	Low
BBB permeant	No
P-gp substrate	Yes
CYP1A2 inhibitor	No
CYP2C19 inhibitor	No
CYP2C9 inhibitor	No
CYP2D6 inhibitor	No
CYP3A4 inhibitor	Yes
Log K_p (skin permeation)	-4.09 cm/s

Druglikeness	
Lipinski	No; 2 violations: MW>500, MLOGP>4.15
Ghose	No; 4 violations: MW>480, WLOGP>5.6, MR>130, #atoms>70
Veber	No; 1 violation: Rotors>10
Egan	No; 1 violation: WLOGP>5.88
Muegge	No; 4 violations: MW>600, XLOGP3>5, #rings>7, Rotors>15
Bioavailability Score	0.17

Medicinal Chemistry	
PAINS	0 alert
Brenk	3 alerts: iodine, phenol_ester, phosphor
Leadlikeness	No; 3 violations: MW>350, Rotors>7, XLOGP3>3.5
Synthetic accessibility	6.56

Figure S30. ADME parameters calculated for mitQ7.

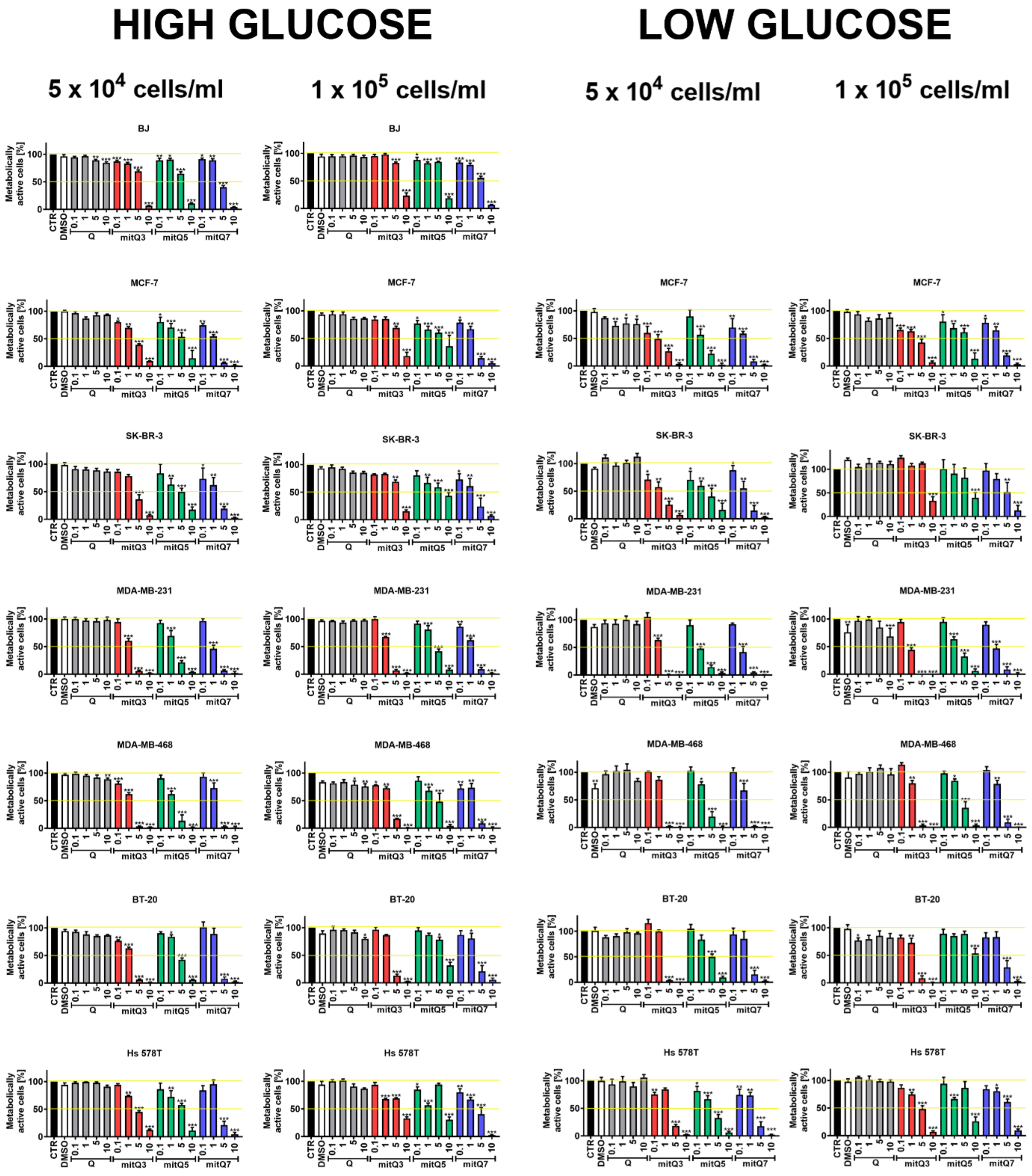

Figure S31. The effects of quercetin (Q) and mito-quercetin derivatives (mitQ3, 5, and 7) on metabolic activity of six breast cancer cell lines, namely, ER-positive MCF-7, HER2-positive SK-BR-3 and four triple negative (TNBC) MDA-MB-231, MDA-MB-468, BT-20, and Hs 578T cells. Normal human fibroblasts (BJ cells) were used for comparison. Cells were treated with Q, mitQ3, 5, and 7 at the concentrations of 0.1, 1, 5 and 10 µM for 24 h. Two glucose concentrations in cell culture medium (DMEM), namely 4.5 g/l (high glucose, HG DMEM) and 1 g/l (low glucose, LG DMEM) and two cell concentrations, namely 5×10^4 cells/ml and 1×10^5 cells/ml were considered. Metabolic activity was assayed using MTT test. Metabolic activity at

untreated conditions (HG or LG conditions, CTR) is considered as 100%. As Q and mitQ3, 5 and 7 were dissolved in DMSO, the effects of DMSO were also studied. Yellow horizontal lines were used to emphasize the effects similar to the effects of untreated control (100% of metabolic activity, HG or LG conditions, CTR) and a decrease in metabolic activity of 50% compared to CTR. Bars indicate SD, n = 3, $^{***}p < 0.001$, $^{**}p < 0.01$, $^{*}p < 0.05$ compared to HG or LG untreated control (CTR) (ANOVA and Dunnett's a posteriori test).

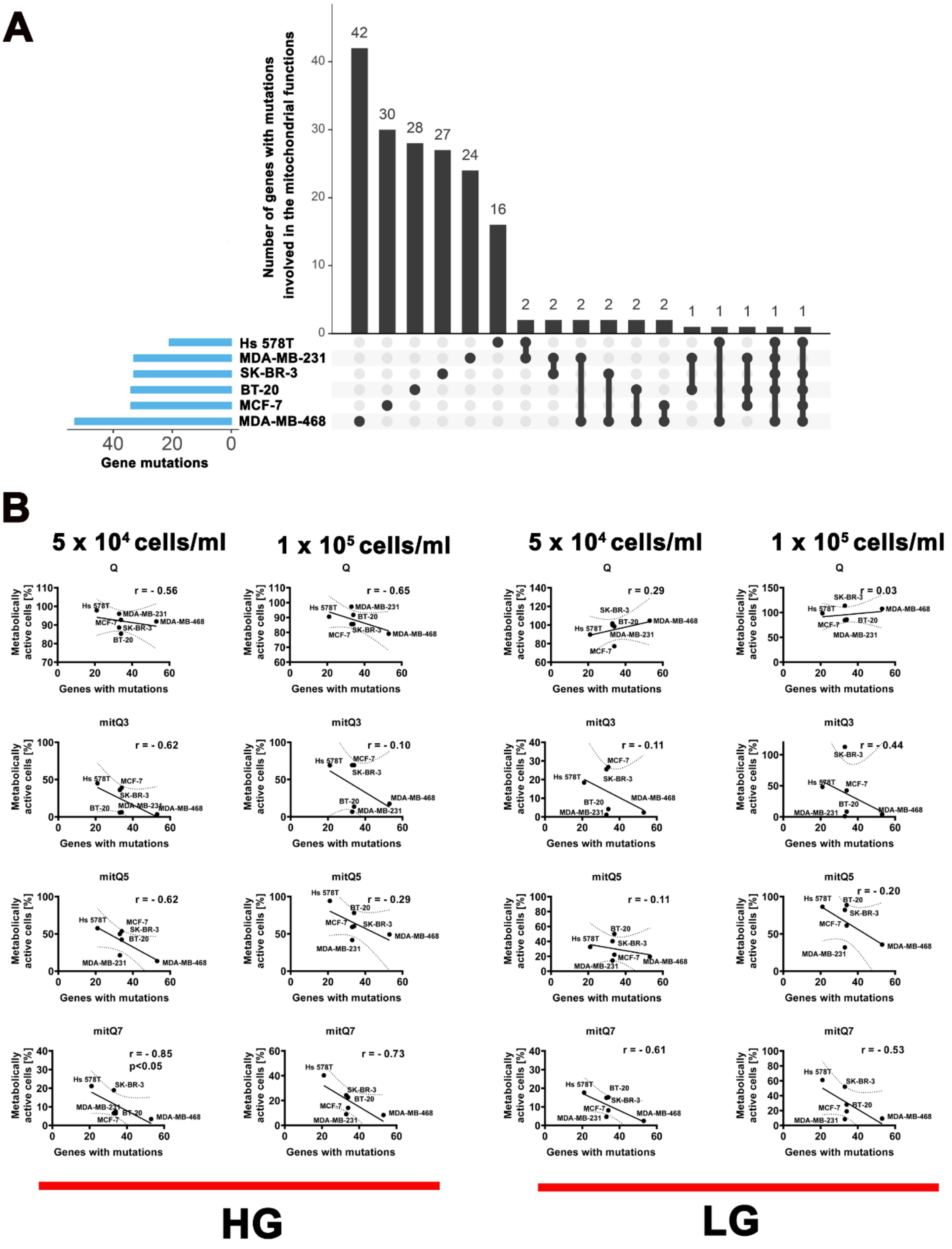
A
Number of genes with mutations involved in the mitochondrial functions
42
30
28
27
24
16
2
1
Hs 578T
MDA-MB-231
SK-BR-3
BT-20
MCF-7
MDA-MB-468
Gene mutations
B
5 x 10^4 cells/ml
1 x 10^5 cells/ml
Q
mitQ3
mitQ5
mitQ7
Metabolically active cells [%]
Genes with mutations
r = - 0.56
r = - 0.65
r = 0.29
r = 0.03
r = - 0.62
r = - 0.10
r = - 0.11
r = - 0.44
r = - 0.62
r = - 0.29
r = - 0.11
r = - 0.20
r = - 0.85
p<0.05
r = - 0.73
r = - 0.61
r = - 0.53
HG
LG

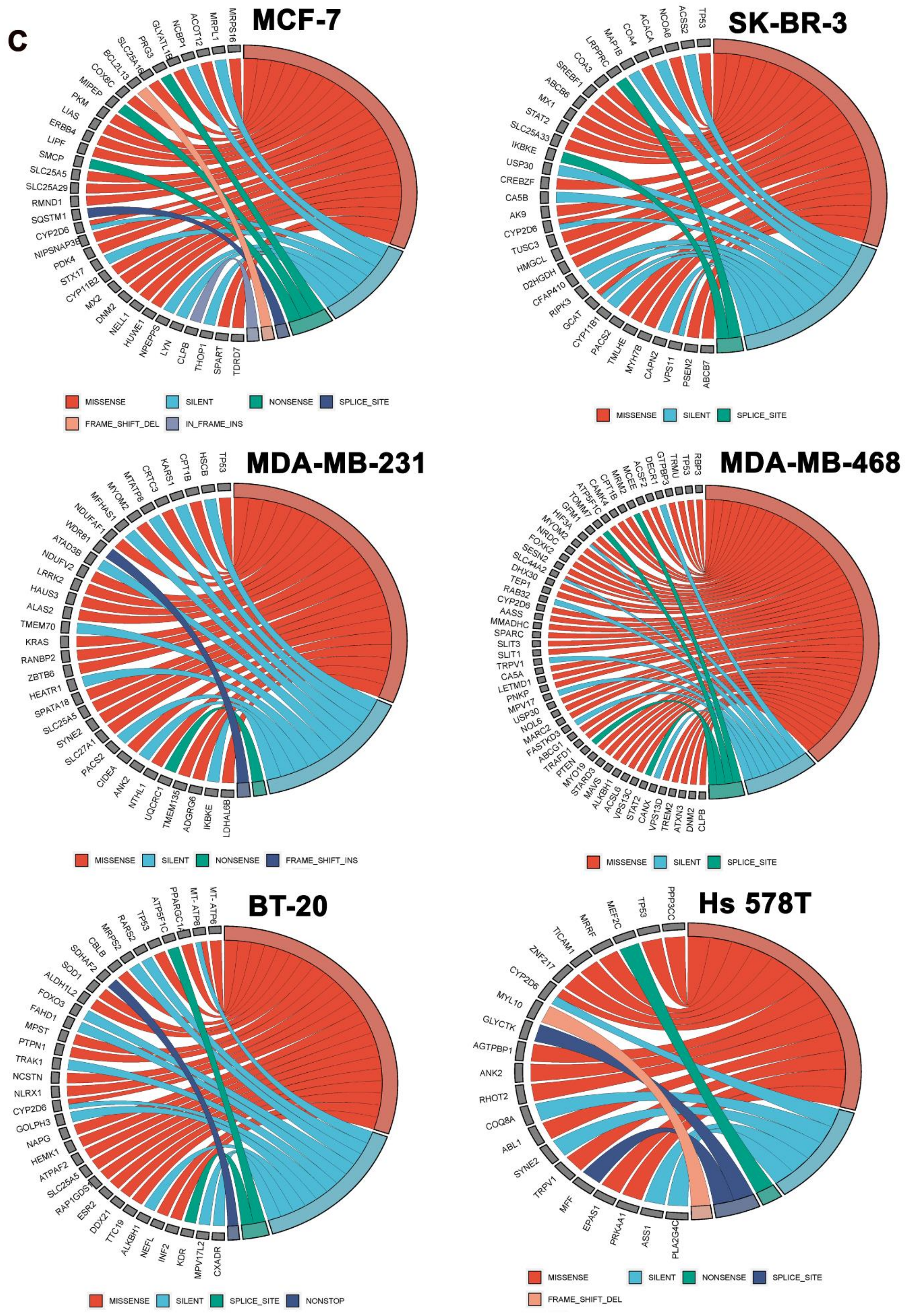

Figure S32. Mitochondria-related gene mutation status (A), correlation analysis between the metabolic activity (MTT-based data) and the number of gene mutations in genes involved in

mitochondrial functions (B), and mutation types (C) in six breast cancer cell lines used in the study. (A) Gene mutation raw data were acquired form DepMap portal (https://depmap.org/portal/). Set intersections in a matrix layout were visualized using the UpSet plot. Total, shared and unique gene mutations in genes involved in mitochondrial functions across six breast cancer cell lines are shown. Blue bars in the *y*-axis represent the number of gene mutations in genes involved in mitochondrial functions in each cell line. Black bars in the *x*-axis represent the number of mutations shared across cell lines connected by the black dots in the body of the plot. (B) Correlation analysis of the data was performed using a linear correlation (Spearman's r) test. The 95% confidence interval, *r* and *p* values are shown. (C) Gene mutation types are presented as a chord diagram (https://www.bioinformatics.com.cn). HG, high glucose DMEM (4.5 g/l); LG, low glucose DMEM (1 g/l).

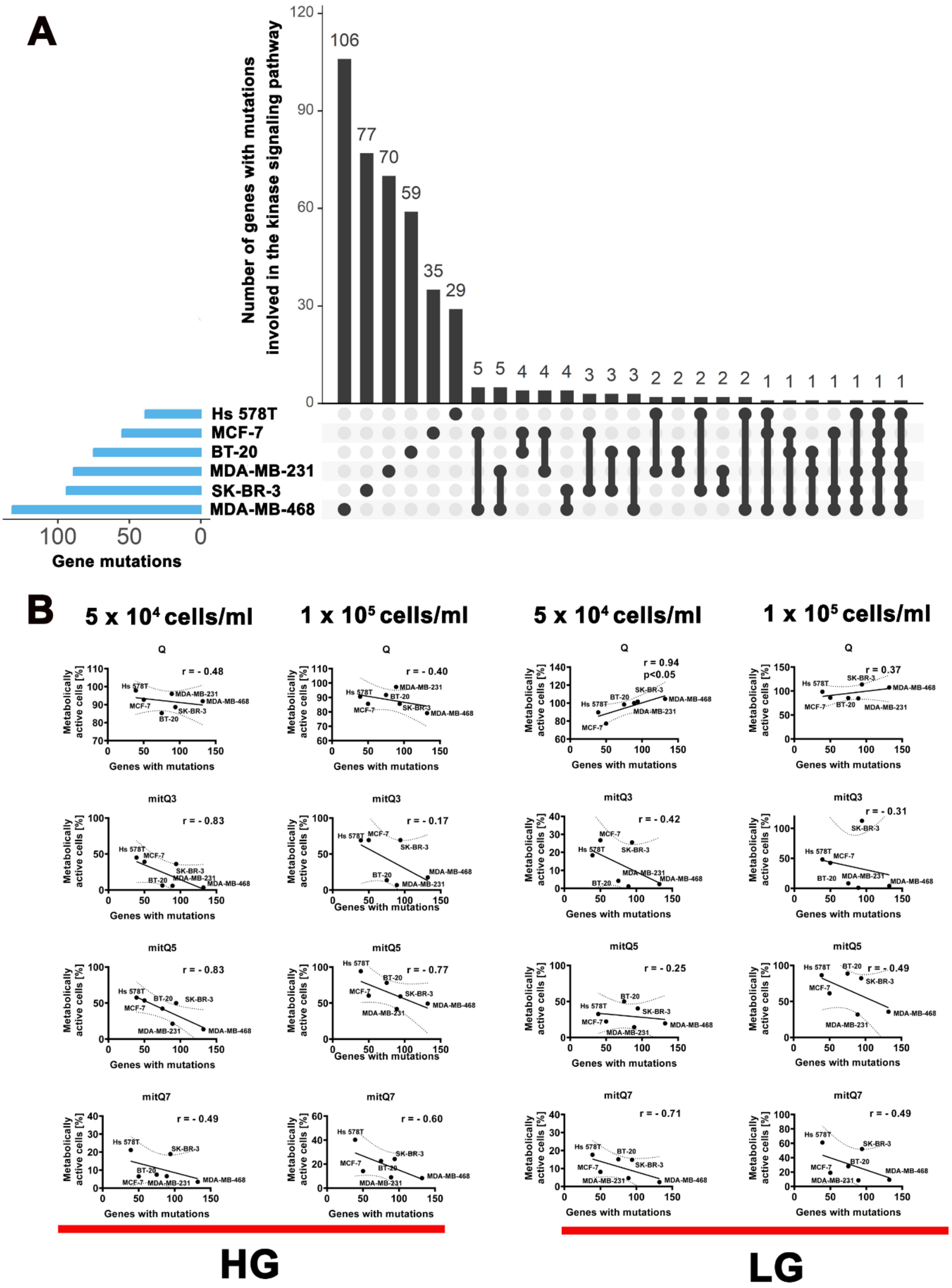
A
Number of genes with mutations involved in the kinase signaling pathway
106
77
70
59
35
29
5 5 4 4 4 3 3 3 2 2 2 2 2 1 1 1 1 1 1 1
Hs 578T
MCF-7
BT-20
MDA-MB-231
SK-BR-3
MDA-MB-468
Gene mutations
B
5 x 10⁴ cells/ml
1 x 10⁵ cells/ml
5 x 10⁴ cells/ml
1 x 10⁵ cells/ml
Q
mitQ3
mitQ5
mitQ7
Metabolically active cells [%]
Genes with mutations
r = - 0.48
r = - 0.40
r = 0.94
p<0.05
r = 0.37
r = - 0.83
r = - 0.17
r = - 0.42
r = - 0.31
r = - 0.83
r = - 0.77
r = - 0.25
r = - 0.49
r = - 0.49
r = - 0.60
r = - 0.71
r = - 0.49
HG
LG

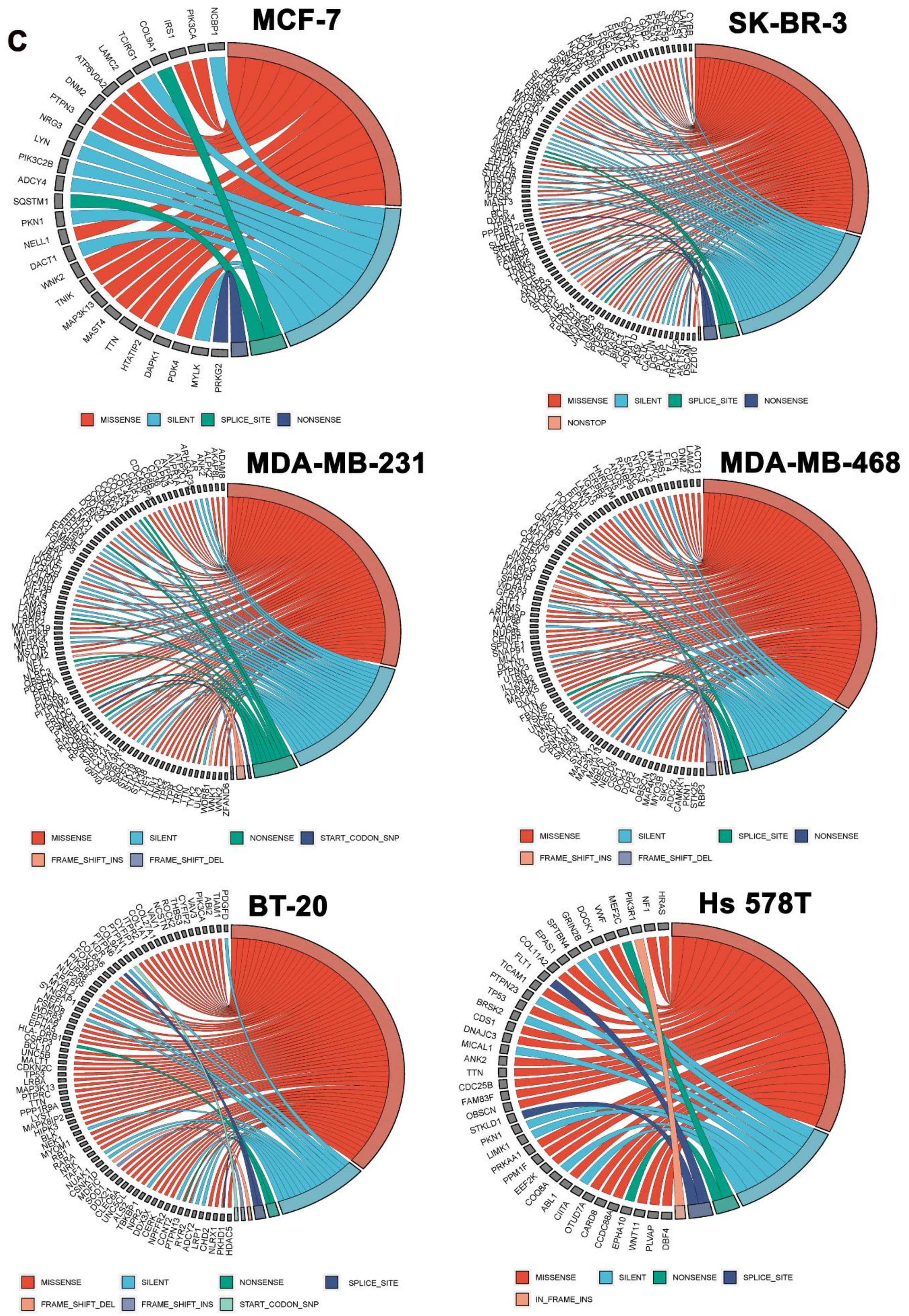

Figure S33. Kinase gene mutation status (A), correlation analysis between the metabolic activity (MTT-based data) and the number of kinase gene mutations (B) and mutation types (C) in six breast cancer cell lines used in the study. (A) Gene mutation raw data were acquired form

DepMap portal (https://depmap.org/portal/). Set intersections in a matrix layout were visualized using the UpSet plot. Total, shared and unique kinase gene mutations across six breast cancer cell lines are shown. Blue bars in the *y*-axis represent the number of kinase gene mutations in each cell line. Black bars in the *x*-axis represent the number of mutations shared across cell lines connected by the black dots in the body of the plot. (B) Correlation analysis of the data was performed using a linear correlation (Spearman's r) test. The 95% confidence interval, *r* and *p* values are shown. (C) Gene mutation types are presented as a chord diagram (https://www.bioinformatics.com.cn). HG, high glucose DMEM (4.5 g/l); LG, low glucose DMEM (1 g/l).

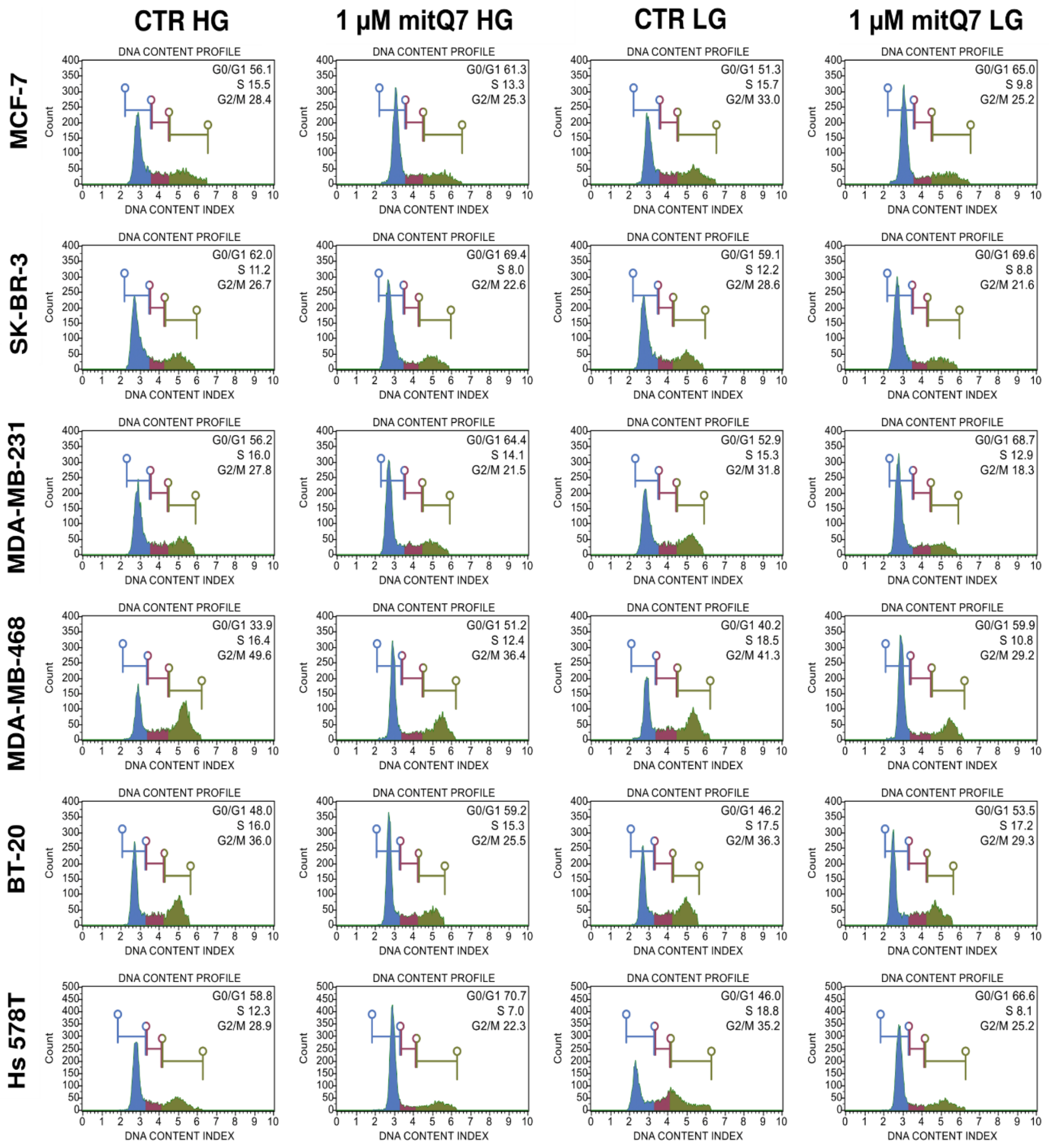

Figure S34. MitQ7-mediated changes in the phases of cell cycle in six breast cancer cell lines, namely, ER-positive MCF-7, HER2-positive SK-BR-3 and four triple negative (TNBC) MDA-MB-231, MDA-MB-468, BT-20, and Hs 578T cells. Breast cancer cells were treated with 1 µM mitQ7 for 24 h. DNA content-based analysis of cell cycle using flow cytometry and dedicated DNA staining. Representative histograms are shown. HG, high glucose DMEM (4.5 g/l); LG, low glucose DMEM (1 g/l); CTR, untreated control.

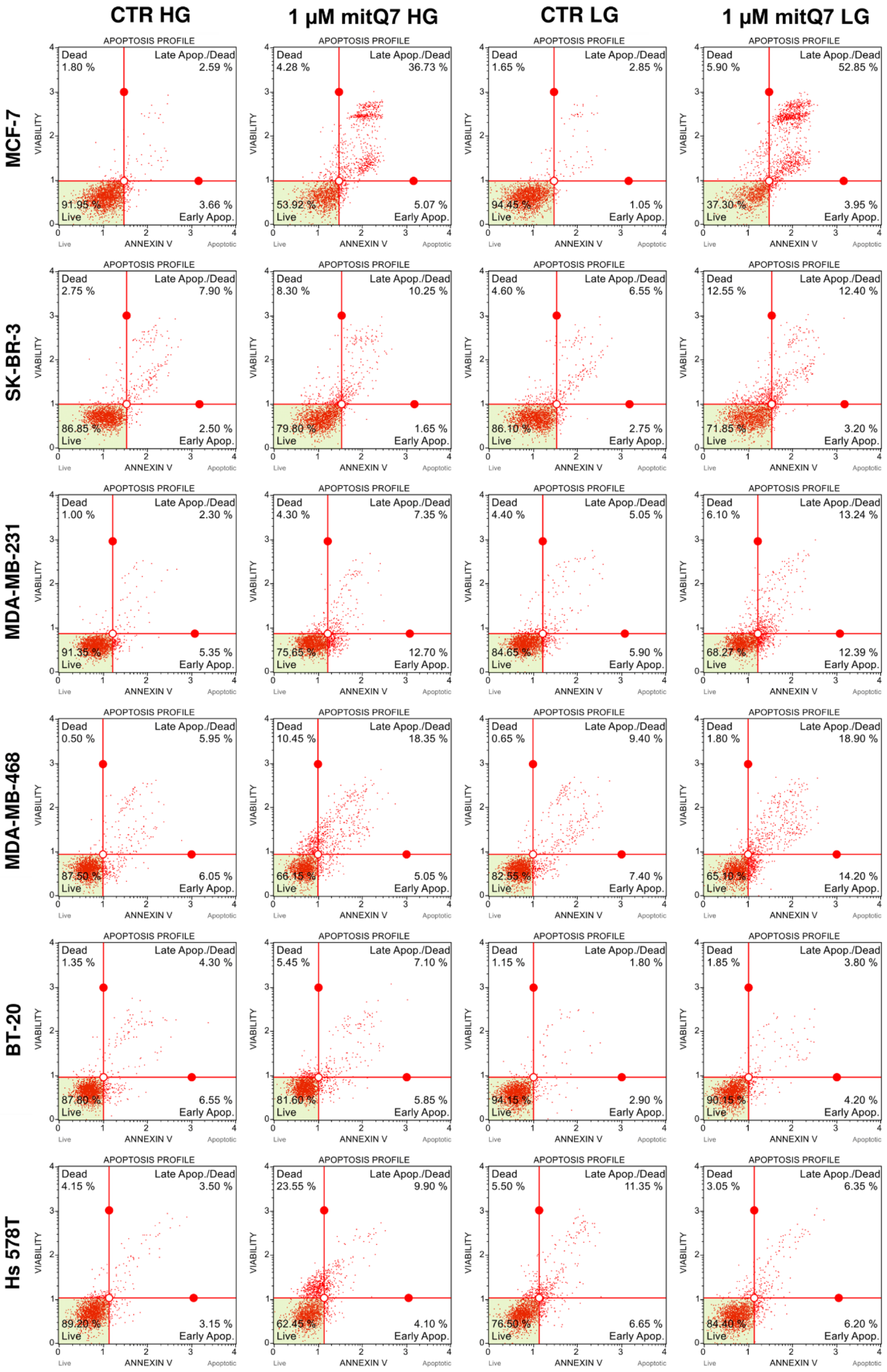

Figure S35. MitQ7-induced apoptosis in six breast cancer cell lines, namely, ER-positive MCF-7, HER2-positive SK-BR-3 and four triple negative (TNBC) MDA-MB-231, MDA-MB-468, BT-20, and Hs 578T cells. Breast cancer cells were treated with 1 μM mitQ7 for 24 h. Apoptosis

was assayed using flow cytometry and Annexin V staining (apoptotic marker) and 7-AAD staining (necrotic marker). Four subpopulations were revealed, namely live cells (dual staining-negative), early apoptotic cells (Annexin V-positive), late apoptotic cells (dual staining-positive) and necrotic cells (7-AAD-positive). Representative dot plots are shown. HG, high glucose DMEM (4.5 g/l); LG, low glucose DMEM (1 g/l); CTR, untreated control.

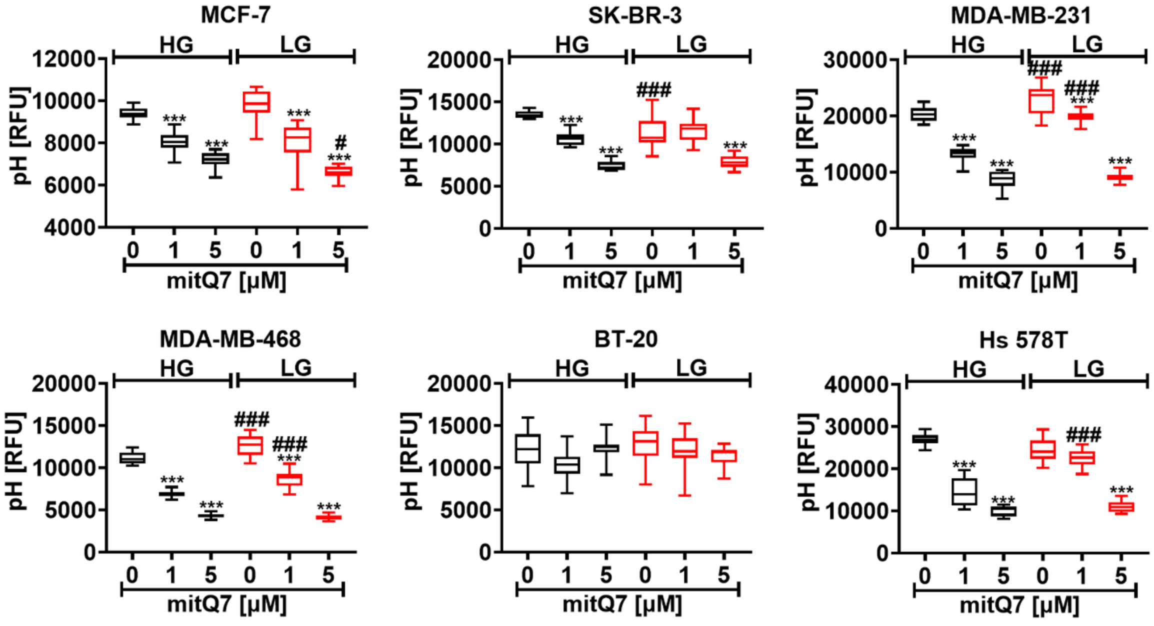

Figure S36. MitQ7-mediated changes in intracellular pH in six breast cancer cell lines, namely, ER-positive MCF-7, HER2-positive SK-BR-3 and four triple negative (TNBC) MDA-MB-231, MDA-MB-468, BT-20, and Hs 578T cells. Breast cancer cells were treated with 1 µM or 5 µM mitQ7 for 24 h. Imaging cytometry and dedicated protocol was considered. Intracellular alkalization was monitored as a decrease in fluorescent signals and is presented as relative fluorescence units (RFU). Box and whisker plots are shown, n = 3, $^{***}p < 0.001$ compared to HG or LG untreated control (ANOVA and Dunnett's a posteriori test), $^{\#\#\#}p < 0.001$, $^{\#}p < 0.05$ compared to HG corresponding conditions (ANOVA and Dunnett's a posteriori test). HG, high glucose DMEM (4.5 g/l); LG, low glucose DMEM (1 g/l).

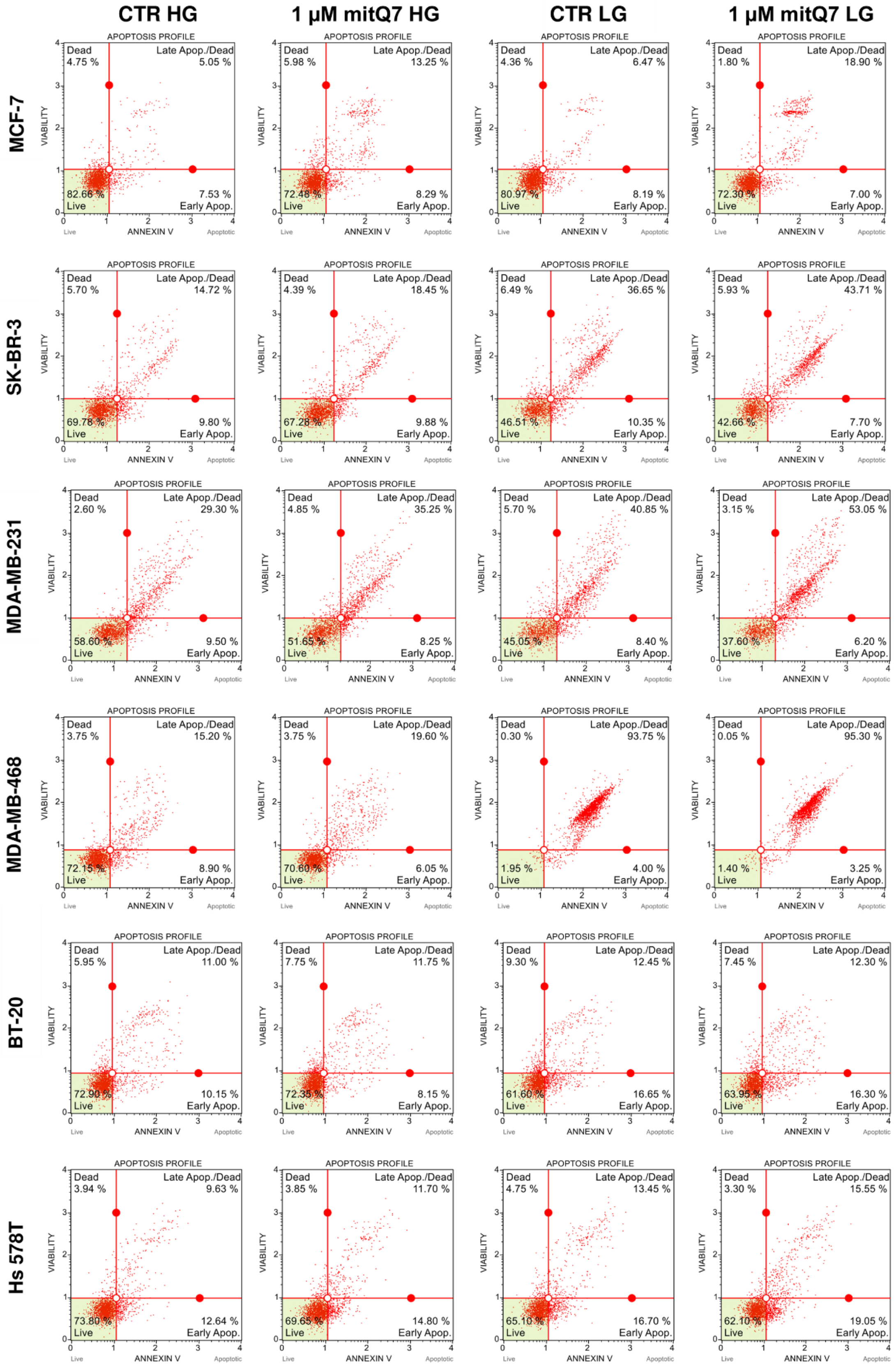

Figure S37. MitQ7-mediated senolytic activity in doxorubicin-induced senescent breast cancer cells. MCF-7, SK-BR-3, MDA-MB-231, MDA-MB-468, BT-20, and Hs 578T cells were treated with 35 nM doxorubicin for 24 h and left for growth for 7 days after drug removal to

induce senescence program. Doxorubicin-induced senescent breast cancer cells were then treated with 1 µM mitQ7 for 24 h and apoptosis was assayed using flow cytometry and Annexin V staining. Representative dot plots are shown. HG, high glucose DMEM (4.5 g/l); LG, low glucose DMEM (1 g/l); CTR, untreated control.

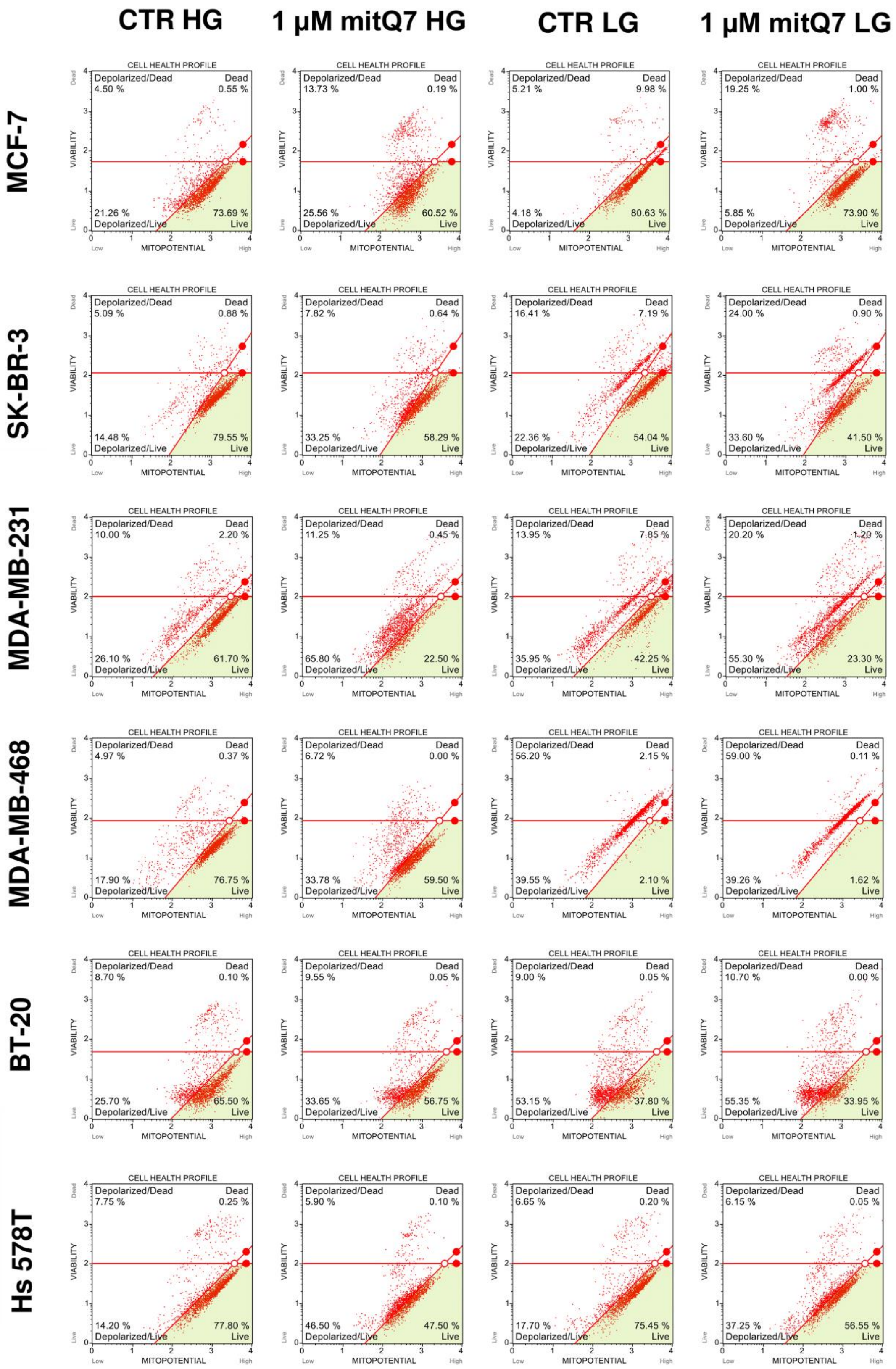

Figure S38. MitQ7-mediated changes in mitochondrial transmembrane potential in doxorubicin-induced senescent breast cancer cells. MCF-7, SK-BR-3, MDA-MB-231, MDA-MB-468, BT-20, and Hs 578T cells were treated with 35 nM doxorubicin for 24 h and left for

growth for 7 days after drug removal to induce senescence program. Doxorubicin-induced senescent breast cancer cells were then treated with 1 µM mitQ7 for 24 h and changes in mitochondrial transmembrane potential were revealed using flow cytometry and dedicated mitopotential probe. Four cell subpopulations were distinguished, namely cells with intact mitochondrial membrane and 7-AAD-negative (live), cells with depolarized mitochondrial membrane and 7-AAD-negative (depolarized/live), cells with depolarized mitochondrial membrane and 7-AAD-positive (depolarized/dead), and cells with intact mitochondrial membrane and 7-AAD-positive (dead). Representative dot plots are shown. HG, high glucose DMEM (4.5 g/l); LG, low glucose DMEM (1 g/l); CTR, untreated control.
